# Cotranscriptional demethylation induces global loss of H3K4me2 from active genes in *Arabidopsis*

**DOI:** 10.15252/embj.2023113798

**Published:** 2023-10-18

**Authors:** Shusei Mori, Satoyo Oya, Mayumi Takahashi, Kazuya Takashima, Soichi Inagaki, Tetsuji Kakutani

**Affiliations:** ^1^ Department of Biological Sciences, Graduate School of Science The University of Tokyo Tokyo Japan; ^2^ National Institute of Genetics Shizuoka Japan

**Keywords:** histone demethylase, histone methylation, RNA polymerase II, transcription, Chromatin, Transcription & Genomics, Plant Biology

## Abstract

Based on studies of animals and yeasts, methylation of histone H3 lysine 4 (H3K4me1/2/3, for mono‐, di‐, and tri‐methylation, respectively) is regarded as the key epigenetic modification of transcriptionally active genes. In plants, however, H3K4me2 correlates negatively with transcription, and the regulatory mechanisms of this counterintuitive H3K4me2 distribution in plants remain largely unexplored. A previous genetic screen for factors regulating plant regeneration identified *Arabidopsis* LYSINE‐SPECIFIC DEMETHYLASE 1‐LIKE 3 (LDL3), which is a major H3K4me2 demethylase. Here, we show that LDL3‐mediated H3K4me2 demethylation depends on the transcription elongation factor Paf1C and phosphorylation of the C‐terminal domain (CTD) of RNA polymerase II (RNAPII). In addition, LDL3 binds to phosphorylated RNAPII. These results suggest that LDL3 is recruited to transcribed genes by binding to elongating RNAPII and demethylates H3K4me2 cotranscriptionally. Importantly, the negative correlation between H3K4me2 and transcription is significantly attenuated in the *ldl3* mutant, demonstrating the genome‐wide impacts of the transcription‐driven LDL3 pathway to control H3K4me2 in plants. Our findings implicate H3K4me2 demethylation in plants as chromatin records of transcriptional activity, which ensures robust gene control.

## Graphical abstract

The histone demethylase LDL3 travels with RNA polymerase II, leaving a record of gene transcription.

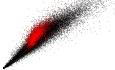

## Introduction

In eukaryotes, transcription and histone modifications influence each other, generating a basis of epigenetic memory (Ng *et al*, 2003; Krogan *et al*, 2003a, 2003b; Inagaki *et al*, 2010; Soares *et al*, 2017; Holoch *et al*, 2021). Methylation of histone H3 lysine 4 (H3K4me) is one of the highly conserved histone modifications found in actively transcribed genes. H3K4me occurs in three different forms: mono‐, di‐, and tri‐methylation (H3K4me1/2/3, respectively). H3K4me3 and H3K4me2 accumulate predominantly in regions near the transcription start site (TSS), whereas H3K4me1 is distributed within transcribed regions. This pattern is conserved among eukaryotes, including yeast (Pokholok *et al*, 2005), animals (Barski *et al*, 2007), and plants (Zhang *et al*, 2009; Oya *et al*, 2022a). H3K4me is widely considered an active mark even though this concept is still under debate (Henikoff & Shilatifard, 2011; Howe *et al*, 2017; Oya *et al*, 2022a). Indeed, while H3K4me3 is strongly correlated with the transcription level in many species of fungi, animals, and plants (Barski *et al*, 2007; Zhang *et al*, 2009; Howe *et al*, 2017; Oya *et al*, 2022a), correlations with transcription levels vary for H3K4me2 and H3K4me1. Intriguingly, H3K4me2 is negatively correlated with transcription levels in plants (Liu *et al*, 2019). This counterintuitive observation is consistent with other studies that suggest that H3K4me2 negatively affects transcription (Kim *et al*, 2012; Ishihara *et al*, 2019a; Liu *et al*, 2019; Oya *et al*, 2022a; Wang *et al*, 2022). An important question is how the negative correlation between H3K4me2 and transcription is established, presumably through bidirectional interactions between transcription machinery and regulators of H3K4me2.

The distributions of H3K4me1/2/3 should be determined by H3K4 methyltransferases, as well as demethylases, most likely through interaction with other chromatin components, including the transcription machinery. An extensively studied example is the interaction between the transcription machinery and the yeast H3K4 methyltransferase Set1. Set1 binds transcription machinery (Ng *et al*, 2003; Bae *et al*, 2020), and recruitment of the methyltransferase to RNA polymerase II is required for histone H3 methylation (Wood *et al*, 2003; Krogan *et al*, 2003a, 2003b). However, many eukaryotes have multiple methyltransferases in addition to Set1 orthologues, and demethylases also contribute to the generation of the H3K4me1/2/3 pattern (Zhou & Ma, 2008; Shilatifard, 2012; Cenik & Shilatifard, 2021; Oya *et al*, 2022a). Notably, the targeting mechanisms and significance of H3K4 demethylases for the generation of H3K4me1/2/3 distribution patterns remain largely unexplored.

Genetic screens for factors mediating silencing by H3K9me have identified LYSINE‐SPECIFIC DEMETHYLASE 1‐LIKE 2 (LDL2) in *Arabidopsis* (Inagaki *et al*, 2017). LDL2 is a member of four *Arabidopsis* homologs of human LSD1, a FAD‐dependent lysine‐specific histone H3K4 demethylase (Shi *et al*, 2004; Jiang *et al*, 2007; Rudolph *et al*, 2007). LDL2 mediates the removal of H3K4me1 from the gene body that accumulates H3K9me2, which in turn directs transcriptional repression. FLOWERING LOCUS D (FLD), another member of the LSD1‐like proteins in *Arabidopsis*, controls transcriptional elongation by removing H3K4me1 from bodies of convergently or bidirectionally transcribed genes (Inagaki *et al*, 2021). These results suggest that H3K4me1 positively controls transcription and that H3K4me1 demethylases contribute to proper epigenome patterning.

Another LSD1‐like protein, LDL3, demethylates H3K4me2 in thousands of protein‐coding genes (Ishihara *et al*, 2019a). The *ldl3* mutants compromise shoot regeneration from callus, likely because H3K4me2 demethylation by LDL3 is required for the activation of genes involved in shoot regeneration (Ishihara *et al*, 2019a). In the *ldl3* mutant, the expression level of the flowering repressor gene *FLOWERING LOCUS C* (*FLC*) is decreased, and flowering is accelerated compared to wild type (WT) plants (Martignago *et al*, 2019). Although these results demonstrate the importance of LDL3‐mediated H3K4me2 removal for gene activation, it remains unknown how LDL3 is recruited to its target genes.

To elucidate the chromatin‐targeting mechanism of LDL3, we used a machine learning algorithm to identify candidate chromatin features that are targeted by LDL3 for its recruitment. Subsequent genetic and biochemical analyses on the identified candidate features (histone modifications and RNAPII phosphorylation) revealed that LDL3 interacts with CTD‐phosphorylated RNAPII and demethylates H3K4me2 cotranscriptionally. As genetic manipulations of H3K4me2 in plants suggest its negative effect on transcription (van Dijk *et al*, 2010; Ishihara *et al*, 2019a; Liu *et al*, 2019; Oya *et al*, 2022a; Wang *et al*, 2022), we propose that H3K4me2 and transcription machinery interact bidirectionally, providing an epigenetic mechanism for robust gene control.

## Results

### Factors associated with LDL3‐dependent H3K4me2 removal

**Figure 1:**
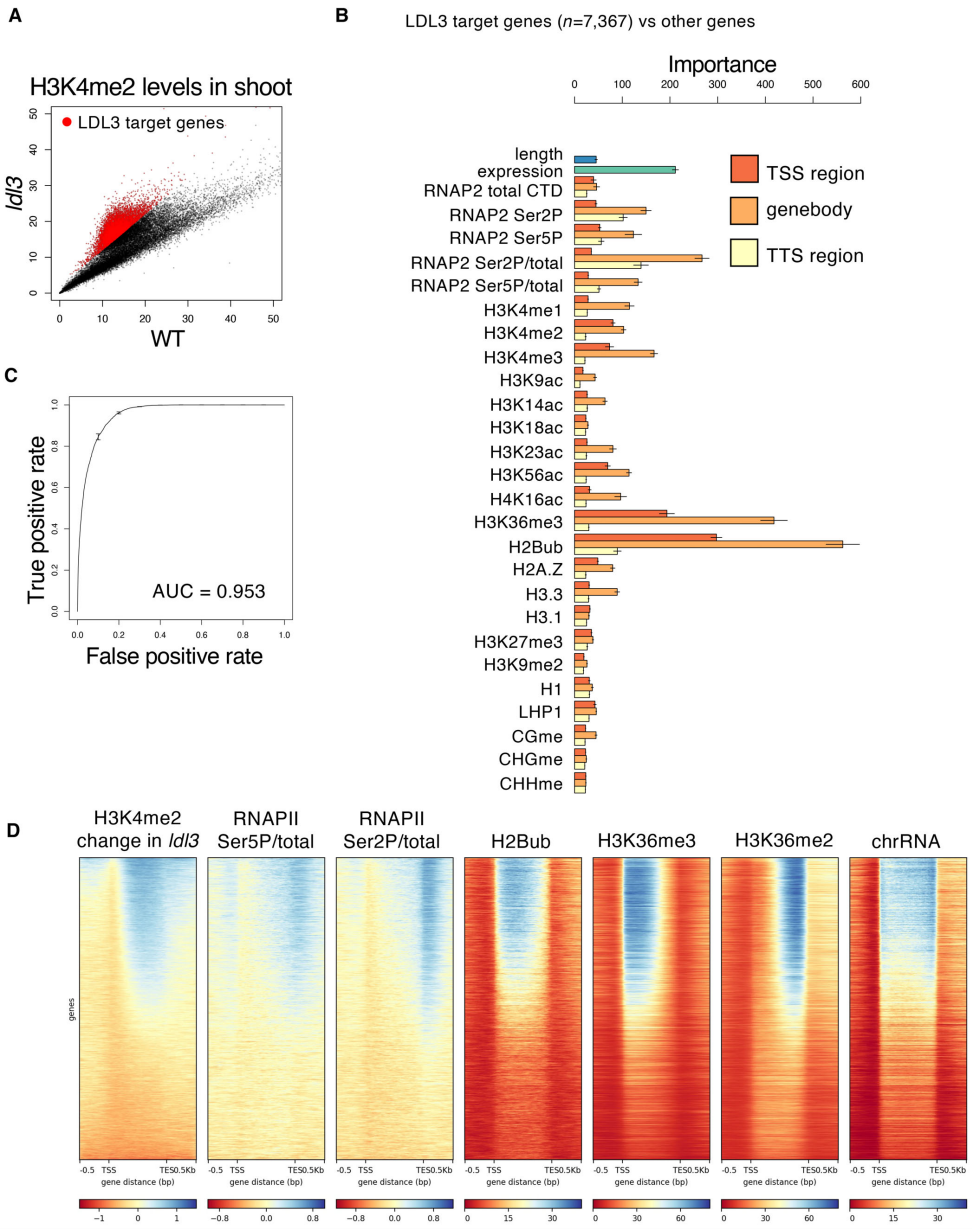
Factors associated with LDL3‐dependent H3K4me2 removal H3K4me2 levels in shoot tissues of the *ldl3* mutants compared with that of WT. Each dot represents RPKM within each protein‐coding gene. Red dots, protein‐coding genes with hyper‐H3K4me2 in *ldl3* (H3K4me2 levels in *ldl3* (RPKM) ‐ H3K4me2 levels in WT (RPKM) > 2; 7,367 genes).Chromatin features predictive of the genes that gain H3K4me2 in the *ldl3* mutant in the random forest models. Error bars represent the standard deviation of the five technical repeats of training.ROC plot showing the prediction accuracy of the random forest models.Enrichment heatmap depicting ChIP–seq normalized reads. The protein‐coding genes were sorted based on increased gene‐body H3K4me2 levels in *ldl3*.

To determine genes from which LDL3 removes H3K4me2, we first investigated H3K4me2 levels in shoot tissues in the WT and the *ldl3* mutants by chromatin immunoprecipitation sequencing (ChIP‐seq). A total of 7,367 genes with increased H3K4me2 in *ldl3* compared with the WT were defined as LDL3 target genes (Fig 1A). These LDL3 target genes also hyperaccumulate H3K4me2 in the roots and calli of *ldl3* mutants (Data ref: Ishihara *et al*, 2019b) (Appendix Fig S1A–C), although the extent of hyperaccumulation varies among tissues. An increase of H3K4me2 in those genes in *ldl3* was associated with reduced mRNA levels, suggesting that H3K4me2 negatively affects transcription (Appendix Fig S2). This observation is consistent with previous studies using the *ldl3* mutant or other conditions affecting H3K4me2 (van Dijk *et al*, 2010; Ishihara *et al*, 2019a; Liu *et al*, 2019; Oya *et al*, 2022a; Wang *et al*, 2022).

We next explored the mechanism by which LDL3 targets specific genes. We reasoned that LDL3 recognizes specific chromatin feature(s), such as epigenome marks and chromatin proteins, and thus, we screened for chromatin feature(s) that distinguish LDL3 demethylation target genes from the other genes using a machine learning algorithm, random forest. The features examined included gene length, expression level, DNA methylation, histone modifications, and RNAPII levels (Fig 1B). The features that could distinguish the LDL3 target genes and others were histone H2B monoubiquitination (H2Bub), H3K36me3, and phosphorylation/total ratio of RNAPII CTD in the gene body (Fig 1B and C); H2Bub, H3K36me3, and phosphorylated RNAPII highly accumulated in the LDL3 target genes compared to the other genes (Fig 1D). We also determined LDL3 target genes (6,085 genes) by a differential peak calling of H3K4me2 between the WT and the *ldl3* mutants and performed the same analysis as above, with almost identical results (Appendix Fig S3A–C).

### Defects in RNAPII phosphorylation and transcriptional elongation mimic loss of LDL3 function

We next genetically tested whether each of the identified candidate factors functions upstream of H3K4me2 demethylation by LDL3. In the *hub1/2* mutants that lack H2Bub (Cao *et al*, 2008) (Appendix Fig S4A) and the *sdg8* mutant that largely loses H3K36me3 (Dong *et al*, 2008; Xu *et al*, 2008; Oya *et al*, 2022a), H3K4me2 patterns were largely unaffected (Appendix Fig S4B and C). These results indicate that H2Bub and H3K36me3 are dispensable for LDL3‐mediated H3K4me2 removal, although the possibility remains that those features work redundantly in LDL3 recruitment.

**Figure 2:**
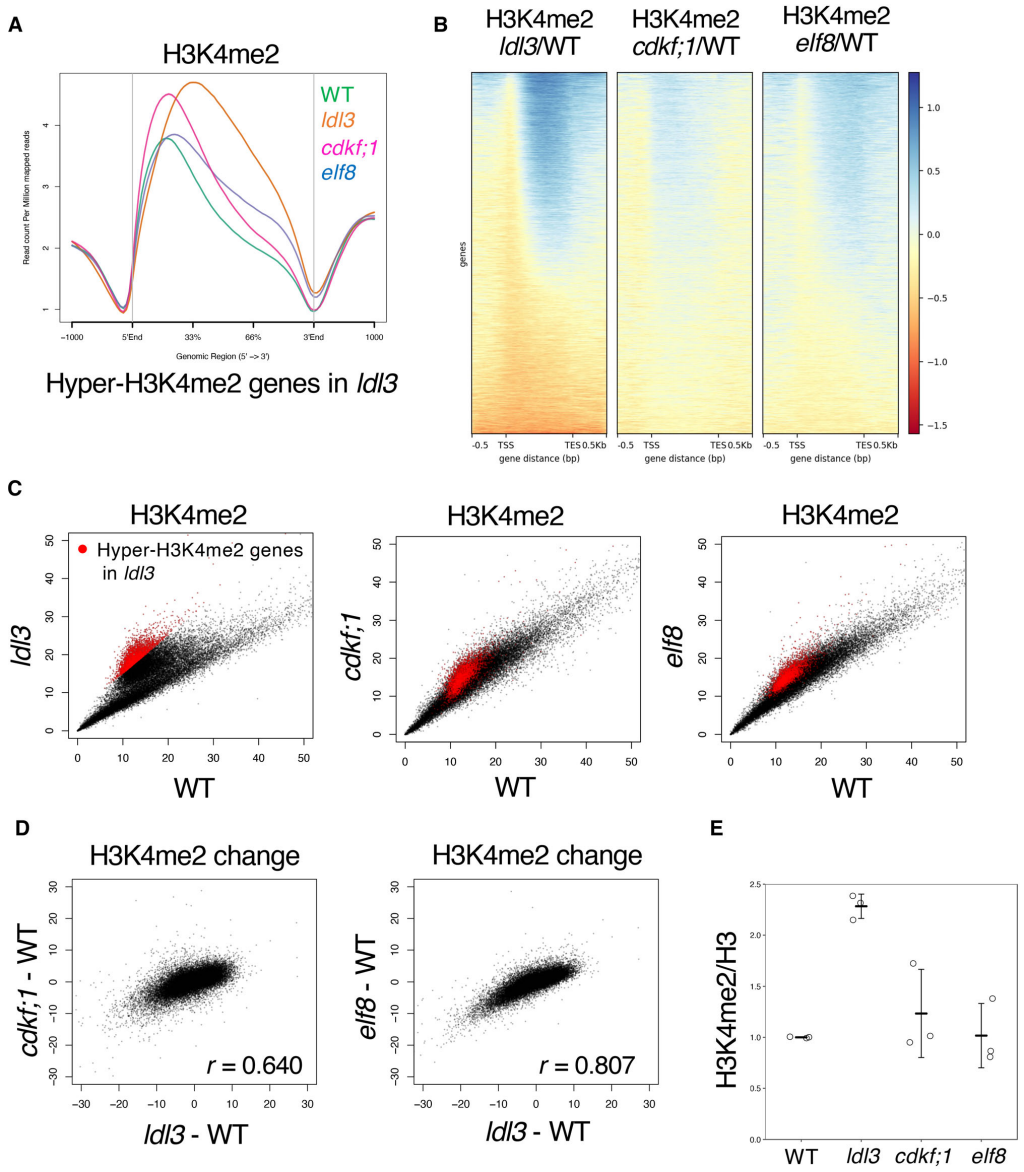
Defects in RNAPII phosphorylation and transcriptional elongation mimic loss of LDL3 function Averaged profiles of H3K4me2 around the LDL3 target genes in each genotype (WT, *ldl3*, *elf8*, *cdkf;1*). The numbers of genes analyzed are 7,367. The ribbons indicate s.e.m.The *elf8* and *cdkf;1* mutant plants show an increase in H3K4me2 for genes affected by *ldl3*. Heatmap of changes in H3K4me2 levels is shown in the three mutants. The protein‐coding genes were sorted based on the increase in gene‐body H3K4me2 levels in *ldl3*.H3K4me2 levels in each of the mutants compared with WT. Each dot represents RPKM within each protein‐coding gene. Red dots, protein‐coding genes with hyper‐H3K4me2 in *ldl3* (H3K4me2 levels in *ldl3* (RPKM) – H3K4me2 levels in WT (RPKM) > 5; 2,809 genes).Relationships between changes in H3K4me2 levels (RPKM) in *elf8* and *cdkf;1*, and in *ldl3* within each of protein‐coding genes compared to WT.Western blotting of H3K4me2 on bulk histone extracted from the mutants. The ratios (H3K4me2/H3) of quantified Western blotting signals are shown as the relative value to WT. Data represents the mean and standard deviation (*n* = 3; technical replicates). Source data are available online for this figure.

To test whether RNAPII phosphorylation acts upstream of H3K4me2 demethylation by LDL3, we examined mutants of an RNAPII CTD kinase, CDKF;1. The *cdkf;1* mutation decreases the levels of both Ser5 and Ser2 phosphorylation (Hajheidari *et al*, 2012). If RNAPII phosphorylation functions upstream of H3K4me2 demethylation by LDL3, the *cdkf;1* mutation will affect LDL3‐induced H3K4me2 demethylation. Indeed, the *cdkf;1* mutant plants showed elevated H3K4me2 levels specifically in genes affected by LDL3 (Fig 2A–D, Appendix Fig S5A–E).

Ser5 and Ser2 phosphorylation of the RNAPII CTD are implicated in transcriptional elongation (Harlen & Churchman, 2017). We therefore explored the relationship between transcriptional elongation and LDL3‐mediated H3K4me2 demethylation using mutants of the transcriptional elongation factor Paf1 complex (Paf1C). Paf1C is known to be involved in RNAPII CTD phosphorylation in yeast and animals (Dronamraju & Strahl, 2014; Yu *et al*, 2015). Paf1C has also been reported to colocalize with phosphorylated RNAPII in plants (Antosz *et al*, 2017). *Arabidopsis* Paf1C consists of VERNALIZATION INDEPENDENCE2 (VIP2)/EARLY FLOWERING7 (ELF7), VIP4, VIP5, VIP6/ELF8, and CDC73/PLANT HOMOLOGOUS TO PARAFIBROMIN (PHP) (Oh *et al*, 2004; Park *et al*, 2010; Yu & Michaels, 2010). The mutants of these Paf1C component genes showed elevated H3K4me2 levels compared with WT, especially in the LDL3 target genes (Appendix Fig S6). This result suggests that Paf1C is required for H3K4me2 demethylation in the LDL3 target genes. In subsequent analyses, we further investigated the function of Paf1C on H3K4me2 demethylation using the *elf8* mutant as a representative of the Paf1C components. The H3K4me2 elevation occurred in largely overlapping genes in the *elf8*, *ldl3* and *cdkf;1* mutants. (Fig 2B–D Appendix Fig S5B and D). Previous studies (Oh *et al*, 2004) proposed that the loss of Paf1C does not affect global H3K4me2/me3 based on Western blotting results, which is consistent with our Western blotting results (Fig 2E), but our ChIP‐seq results showed that Paf1C significantly affects H3K4me2 distribution. H2Bub and H3K36me2/3, which random forest analysis also identified, were altered in the *elf8* mutant (Appendix Fig S7A–C) (Oh *et al*, 2008; Liu *et al*, 2019), but these modifications were not altered in the *ldl3* mutant, suggesting that they may be regulated by ELF8/Paf1C, independently of LDL3. In fact, Paf1C has been reported to genetically and physically interact with the H3K36 methyltransferases, yeast SET2 and *Arabidopsis* SDG8 (Krogan *et al*, 2003a, 2003b; Yang *et al*, 2016).

In the *ldl3* mutant, increased H3K4me2 was associated with a decrease in H3K4me1, but not in H3K4me3 (Appendix Fig S8A, B, and D–F), likely because H3K4me1 is the product of H3K4me2 demethylation by LDL3 (Ishihara *et al*, 2019a). Western blotting and ChIP‐seq results demonstrate that both the *cdkf;1* and *elf8* mutants showed changes in the H3K4me1 pattern analogous to that of *ldl3*, with no reduction in H3K4me3 (Appendix Fig S8A–F). These results suggest that transcriptional elongation and/or RNAPII phosphorylation functions upstream of H3K4me2 demethylation by LDL3.

LDL3 expression levels were not reduced in the *cdkf;1* or in the *elf8* mutant (Appendix Fig S9), indicating that Paf1C and CDKF;1 likely affect LDL3 function or recruitment, not its transcription. In addition, the *cdkf;1* and *elf8* mutants compromised shoot regeneration from callus similar to the *ldl3* mutant (Ishihara *et al*, 2019a) (Appendix Fig S10A and B). These results suggest a novel pathway in which LDL3‐mediated H3K4me2 removal is controlled through the interaction between LDL3 and elongating RNAPII, and this pathway is indispensable for regeneration.

### LDL3 binds to phosphorylated RNAPII *in vivo*

**Figure 3:**
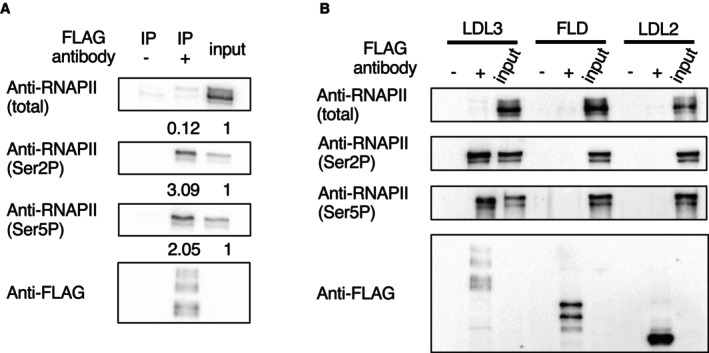
LDL3 is bound to phosphorylated RNAPII *in vivo* LDL3 was immunoprecipitated with anti‐FLAG beads (IP: FLAG) from whole cell lysates and analyzed by immunoblotting using antibodies against Ser5P, Ser2P or total RNAPII CTD. The ratios (IP/input) of quantified Western blotting signals are shown at the bottom of each blot.Co‐IP experiments testing the binding between RNAPII and each of the LSD1 family proteins (LDL3, FLD, LDL2). Source data are available online for this figure.

To test whether the effect of RNAPII on H3K4me2 is mediated by its direct binding to LDL3, we performed coimmunoprecipitation (Co‐IP) analysis using transgenic plants that expressed FLAG‐tagged LDL3. RNAPII was pulled down by IP with an antibody against FLAG (Fig 3A), which showed that LDL3 interacts with RNAPII. LDL3 bound much more strongly to CTD‐phosphorylated RNAPII than to unphosphorylated RNAPII (Fig 3A). Taken together with the colocalization of phosphorylated RNAPII and the LDL3 target genes shown in the heatmap (Fig 1D), it is likely that LDL3 binds to phosphorylated RNAPII and removes H3K4me2 cotranscriptionally. Interestingly, the other LSD1 family H3K4 demethylases, FLD and LDL2 did not show binding to phosphorylated RNAPII comparable to LDL3 (Fig 3B). LDL3, but not LDL2 or FLD, has the SRI domain, which was identified in yeast Set2 (Kizer *et al*, 2005) as a domain specifically binding to the phosphorylated CTD of RNAPII (Rebehmed *et al*, 2014; Appendix Fig S11A). An LDL3 mutant with deletion of the SRI domain (ΔSRI) did not interact with phosphorylated RNAPII as the full‐length LDL3 (Appendix Fig S11A and B) suggesting the role of the SRI domain in LDL3 for binding to CTD‐phosphorylated RNAPII.

### RNAPII phosphorylation and transcriptional elongation are differentially altered in *elf8* and *cdkf;1* mutants

**Figure 4:**
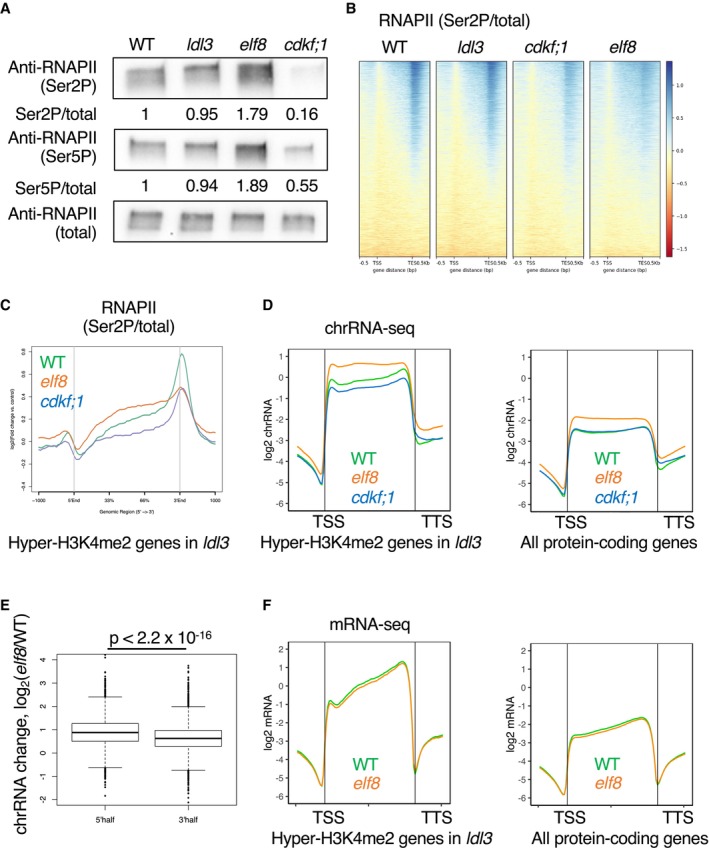
RNAPII phosphorylation and transcriptional elongation are differentially altered in *elf8* and *cdkf;1* mutants Western blotting of protein extracted from seedlings of each genotype. Membranes were probed with antibodies against Ser5P or Ser2P or total RNAPII CTD. The ratios (phosphorylated/total) of quantified Western blotting signal are shown as the relative value to WT.Heatmaps of RNAPII Ser2P levels (Ser2P/total CTD ratio) are shown in WT and the three mutants. The protein‐coding genes were sorted based on increased gene‐body H3K4me2 levels in *ldl3*.Averaged profiles of Ser2P levels around the LDL3 target genes for each genotype (WT, *elf8*, *cdkf;1*). The numbers of genes analyzed are 7,367. The ribbons indicate s.e.m.Averaged profiles of chrRNA around the LDL3 target genes (*n* = 7,367) and all protein‐coding genes (*n* = 27,206) for each genotype (WT, *elf8*, *cdkf;1*). The ribbons indicate s.e.m.Box plots comparing the ratio of chrRNA levels in 5′ half of the gene body (left) and 3′ half of the gene body (right) between the WT and *elf8*, in the LDL3 target genes (7,367 genes). The central bands correspond to the median; the upper and lower limits of the box correspond to the upper and lower quantiles; the whiskers indicate the data range within 1.5× of the interquartile range; the dots indicate the outliers. The *P* values are based on Welch's two‐sample *t*‐test.Averaged profiles of mRNA around the LDL3 target genes (*n* = 7,367) and all protein‐coding genes(*n* = 27,206) for each genotype in WT and *elf8*. The ribbons indicate s.e.m. Source data are available online for this figure.

The results above suggest that phosphorylated RNAPII mediates the function of LDL3 in removing H3K4me2. We therefore examined the levels of phosphorylated RNAPII in the *cdkf;1* and *elf8* mutants by Western blotting. As expected from its coding protein, the *cdkf;1* mutant showed decreases in RNAPII phosphorylation levels (both Ser2P and Ser5P) (Fig 4A), which was consistent with a previous study (Hajheidari *et al*, 2012). Unexpectedly, however, the proportion of phosphorylated RNAPII was increased in *elf8* (Fig 4A). To further clarify the effect of *cdkf;1* and *elf8* on RNAPII phosphorylation, we examined genome‐wide patterns of RNAPII phosphorylation by ChIP‐seq analysis. Consistent with the idea that the loss of phosphorylation in the *cdkf;1* mutant mediates its effect on LDL3 function, the ratio of Ser2P/total RNAPII in the gene body and around the transcription termination site (TTS) was decreased in *cdkf;1* in the LDL3 target genes (Fig 4B and C).

In contrast, the ratio of Ser2P/total RNAPII was increased in the gene body in the *elf8* mutant, and the pattern was skewed towards the upstream region (Fig 4B and C). The ratio of Ser5P/total RNAPII in the gene body and around TTS also increased in *elf8* and decreased in *cdkf;1* (Appendix Fig S12A and B). These results suggest that although phosphorylation of RNAPII is necessary for LDL3 function, *elf8* mutation affects LDL3 function through a different pathway.

To determine how the loss of Paf1C and CDKF;1 affects transcription dynamics and LDL3 function, we conducted chromatin‐bound RNA sequencing (chrRNA‐seq) to examine nascent transcribing RNAs (Nojima *et al*, 2015; Inagaki *et al*, 2021). The *elf8* mutant showed a remarkable change in transcriptional dynamics, with a large amount of nascent RNA detected near the TSS (Fig 4D). This trend was more pronounced in the LDL3 target genes. In contrast, chrRNA levels were slightly reduced in *cdkf;1* (Fig 4D). These chrRNA‐seq results were consistent with the RNAPII ChIP‐seq results. In the *elf8* mutant, RNAPII phosphorylation levels and chrRNA levels were increased in the gene body, especially in the 5′ half of the gene (Fig 4E). Since mRNA levels were not increased (Fig 4F), transcriptional elongation was considered to be retarded. Conversely, in the *cdkf;1* mutant, RNAPII phosphorylation and chrRNA levels were reduced, suggesting an overall reduced transcriptional activity caused by reduced RNAPII phosphorylation. The patterns of Ser2P/total RNAPII and chrRNA were not largely affected in the *ldl3* mutant (Appendix Fig S12C and D), suggesting that the increased H3K4me2 level is a consequence but not a cause of altered RNAPII dynamics and phosphorylation. Taken together, Paf1C and CDKF;1 regulate RNAPII phosphorylation and transcriptional elongation in different ways.

H3K4me2 profiles also differed between *elf8* and *cdkf;1*. In the *elf8* mutant, the H3K4me2 level increased in the 3′ half of the target genes (Fig 2A, Appendix Fig S5A), which was consistent with the stalled transcription in the 5′ half of the gene body (Fig 4D and E). In the *cdkf;1* mutant, H3K4me2 increased while maintaining the WT profile (Fig 2A, Appendix Fig S5A), possibly reflecting reduced overall transcription activity (Fig 4D). Differential changes in RNAPII phosphorylation and transcriptional elongation in *cdkf;1* and *elf8* may generate different patterns of H3K4me2 increase (Appendix Fig S12E).

### Paf1C regulates the localization of LDL3 and affects H3K4me2

**Figure 5:**
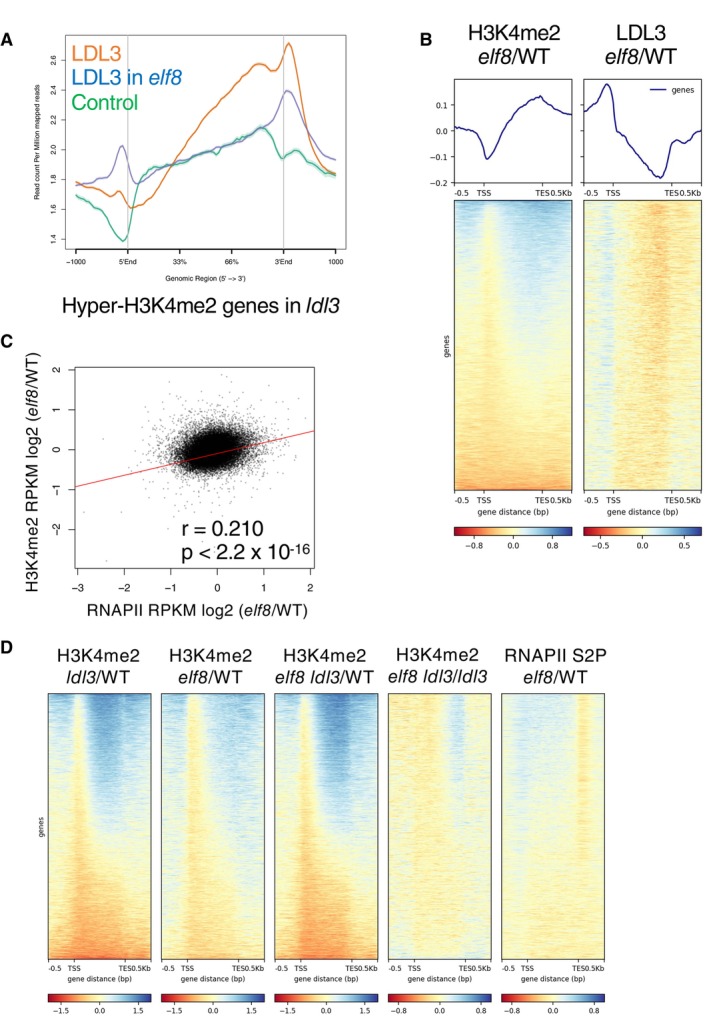
Paf1C regulates the localization of LDL3 and affects H3K4me2 Averaged profile of LDL3 in WT and the *elf8* mutants.Metaplots and heatmaps depicting changes in H3K4me2 (left) or LDL3 (right) levels between WT and *elf8*. The protein‐coding genes were sorted based on increased gene‐body H3K4me2 levels in *elf8*.Relationships between changes in RNAPII levels (RPKM) and changes in H3K4me2 levels (RPKM) in *elf8* within each protein‐coding gene compared to WT.Heatmaps depicting ratios of H3K4me2 or RNAPII S2P levels between denoted genotypes. Developmental phenotypes of the double mutant and each of the single mutants are shown in Appendix Fig S14C. Source data are available online for this figure.

We further explored the mechanism by which Paf1C regulates LDL3‐mediated H3K4me2 demethylation. The direct interaction between LDL3 and phospho‐RNAPII (Fig 3A) supports the hypothesis that Paf1C‐mediated transcriptional elongation directly facilitates the recruitment and/or function of LDL3, and thus, we examined whether the loss of Paf1C affects LDL3 localization. In WT, LDL3 accumulated in the 3′ half of the gene, and its distribution pattern was similar to that of Hyper‐H3K4me2 in *ldl3* and phosphorylated RNAPII‐enrichment in WT (Fig 5A, Appendix Fig S13A and B). In the *elf8* mutant, LDL3 accumulation was decreased in the 3′ half of the genes compared with WT (Fig 5A, Appendix Fig S13A). Hyper‐H3K4me2 region correlated with hypo‐LDL3 region in the *elf8* mutant (Fig 5B). Furthermore, we found a clear positive correlation between estimated transcriptional retardation and H3K4me2 accumulation in the *elf8* mutant (Fig 5C, Appendix Fig S13C). In WT, ELF8 localized to these genes that showed hyperaccumulation of chrRNA, total and phosphorylated RNAPII, and H3K4me2 in *elf8* (Appendix Fig S13D and E). These results suggest that RNAPII‐associated Paf1C ensures transcriptional elongation and recruitment of LDL3 to transcribed regions, and thus regulates H3K4me2 dynamics.

We also genetically tested whether Paf1C and LDL3 work through the same pathway for H3K4me2 regulation by analyzing the *elf8 ldl3* double mutant. The *elf8 ldl3* double mutant showed an increase in H3K4me2 similar to that in the *ldl3* single mutant (Fig 5D, Appendix Fig S14A–C), suggesting that the accumulation of H3K4me2 in the *elf8* mutant is mostly through compromising the function of LDL3.

On the other hand, a subset of genes showed higher H3K4me2 levels (cluster 1: 10,329 genes, Fig 5D, Appendix Fig S14A) in the *elf8 ldl3* double mutant than in the *ldl3* single mutant, suggesting the presence of additional pathway(s) that regulate H3K4me2 cotranscriptionally. Interestingly, in the genes that accumulate H3K4me2 further in the *elf8 ldl3* double mutant than in the *ldl3* single mutant, the level of RNAPII accumulation was increased in *elf8* compared with WT (Fig 5D, Appendix Fig S14A). It is possible that transcription retardation due to the *elf8* mutation increases the time period that H3K4 methyltransferase(s) spend in the gene body, which leads to increased H3K4me2 level (Fong *et al*, 2017; Soares *et al*, 2017).

### LDL3 is responsible for the negative correlation between H3K4me2 and transcription

**Figure 6:**
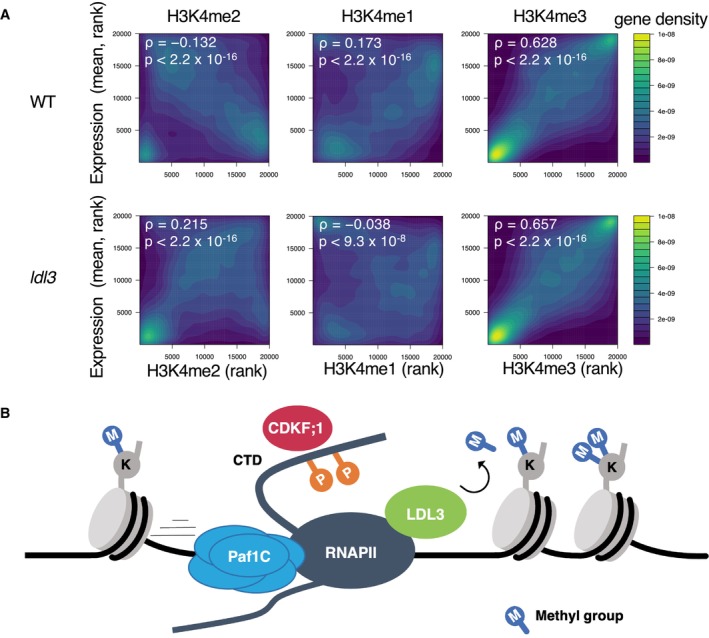
LDL3 is responsible for the negative correlation between H3K4me2 and transcription Correlations between H3K4me and transcription level of protein‐coding genes in wild type (WT) and *ldl3* mutant plants. Protein‐coding genes are ranked by H3K4me1 or me2 or me3 (x‐axis, RPKM) and expression levels (y‐axis, chrRNA‐seq, RPKM). The densities of genes are visualized as heat maps. ρ: Spearman's correlation coefficient. The *P* value was determined based on Spearman's rank correlation test. Genes for which no transcription was detected were excluded.Model for co‐transcriptional H3K4me2 demethylation by LDL3. LDL3 recognizes RNAPII phosphorylation and binds to RNAPII, and thus H3K4me2 is removed co‐transcriptionally. Source data are available online for this figure.

Considering that the LDL3 target genes are highly expressed (Fig 1D) and that LDL3 interacts with RNAPII (Fig 3A and B), we speculated that LDL3 contributes to the negative correlation between H3K4me2 and transcription (Liu *et al*, 2019) by removing H3K4me2 from highly transcribed genes. Indeed, the negative correlation was attenuated and even became positive in the *ldl3* mutant (Fig 6A). The negative correlation was also attenuated in the *cdkf;1* and *elf8* mutants, although the extent was weaker than that in *ldl3* (Appendix Fig S15). This transition from negative to positive correlation between H3K4me2 and transcription in *ldl3* is more pronounced in the gene body region than in the TSS region (Appendix Figs S16 and S17). In the TSS region of transcribed genes, H3K4me3 is highly accumulated and H3K4me2 levels are relatively low because of the mutual exclusivity of these methylation states. These results suggest that LDL3 demethylates H3K4me2 cotranscriptionally in gene bodies, resulting in the establishment of a negative correlation between H3K4me2 and transcription level. We and others have previously identified ATXR3 and ATX3/4/5 as H3K4 methyltransferases which contribute to H3K4me2/me3 (Berr *et al*, 2010; Guo *et al*, 2010; Chen *et al*, 2017; Oya *et al*, 2022a). In the *atxr3* mutants, the correlation between H3K4me2 in the gene body region and transcription is altered towards negative (Appendix Fig S18; Data ref: Oya *et al*, 2022b), and therefore we suggest that ATXR3 deposits H3K4me2 cotranscriptionally in addition to H3K4me3 (Oya *et al*, 2022a). Furthermore, the positive correlation between transcription and H3K4me1 levels, which was seen in WT, was attenuated in the *ldl3*, *cdkf;1*, and *elf8* mutants (Fig 6A, Appendix Fig S15), suggesting that the conversion from H3K4me2 to H3K4me1 by LDL3 contributes to this positive correlation. These results indicate that LDL3 demethylates H3K4me2 cotranscriptionally to contribute to the higher H3K4me1 and lower H3K4me2 levels within actively transcribed genes.

## Discussion

In this study, we searched for candidate factors that determine the functional pathway of LDL3 through an initial screening with random forest analyses and subsequent genetic tests. By this approach, we identified RNAPII phosphorylation and transcriptional elongation as factors functioning upstream of LDL3‐mediated H3K4me2 demethylation.

The involvement of RNAPII phosphorylation in LDL3 function was further substantiated by the biochemical results showing that LDL3 binds to phosphorylated RNAPII. Interestingly, such binding was not detected for two other LSD1‐type *Arabidopsis* demethylases, LDL2 and FLD, which remove H3K4me1. LDL3, but not LDL2 and FLD, has the SRI domain. In yeast, the SRI domain has been shown to specifically bind to the phosphorylated RNAPII (Kizer *et al*, 2005; Rebehmed *et al*, 2014). The SRI domain in LDL3 is necessary for its binding to phosphorylated RNAPII (Appendix Fig S11A and B). While mammalian LSD1 family proteins do not have the SRI domain, the SRI domain is found in LDL3 orthologues in flowering plants such as rice and poplar, the lycophyte *Selaginella moellendorffi*, and the moss *Physcomitrella patens* (Rebehmed *et al*, 2014) (Appendix Fig S19), suggesting that cotranscriptional H3K4 demethylation via the SRI domain may be conserved among land plants.

The *cdkf;1* mutant showed an increase in H3K4me2 in the LDL3 targets, most likely through a decrease in RNAPII phosphorylation. Interestingly, however, the *elf8* mutant showed an increase in RNAPII phosphorylation levels despite an increase in H3K4me2. On the other hand, a delay in transcriptional elongation was prominent in the *elf8* mutant but not in the *cdkf;1* mutant. These results suggest that *elf8* and *cdkf;1* affect LDL3 function through distinct pathways. The *elf8* mutation decreases LDL3 accumulation and affects H3K4me2 in the ELF8‐bound genes which shows retardation of transcriptional elongation in *elf8*. These results suggest that defects in Paf1C lead to transcriptional retardation and affect LDL3‐mediated H3K4me2 demethylation directly. It is also possible that Paf1C is required for the binding of LDL3 to phosphorylated RNAPII, as recruitment of LDL3 to its target is compromised in the *elf8* mutant.

Cotranscriptional H3K4 methylation has been reported in yeast and animals (Woo *et al*, 2017). RNAPII phosphorylation is known to stimulate H3K4 methylation by Set1‐COMPASS, and Paf1C is also important for Set1 recruitment (Ng *et al*, 2003; Krogan *et al*, 2003a, 2003b). The cotranscriptional effect is also observed in the Set1‐type *Arabidopsis* methyltransferases ATXR3 and ATXR7, which deposit H3K4me3 and H3K4me1, respectively (Oya *et al*, 2022a), although Paf1C is not essential in this mechanism in *Arabidopsis* (Oh *et al*, 2004). On the other hand, our study shows that Paf1C and phosphorylation of RNAP II contribute to the LDL3‐mediated removal of H3K4me2 in *Arabidopsis*. LDL3 contributes to the plant‐specific negative correlation between H3K4me2 and transcription by the transcription‐driven demethylation of H3K4.

Thus, transcription induces high H3K4me1/3 states by Set1‐type methyltransferases, but it also induces low H3K4me2 by transcription‐coupled removal of H3K4me2 (Fig 6B). As it has been proposed in previous studies that transcription‐coupled H3K4 regulation may establish a memory of recent transcriptional activity (Ng *et al*, 2003; Muramoto *et al*, 2010; Ding *et al*, 2012), it is possible that H3K4me2 removal also functions as a memory of transcription. This hypothesis is consistent with reports that H3K4me2 negatively affects transcription in plants (*Arabidopsis* and rice) (Liu *et al*, 2019; Wang *et al*, 2022). It is also known that H3K4me2 levels are decreased in transcriptionally upregulated genes under dehydration stress (van Dijk *et al*, 2010). The *ldl3* mutant showed decreased *FLC* expression levels, which affects the developmental transition to environmental stimuli (Martignago *et al*, 2019). Furthermore, LDL3 function is necessary for shoot regeneration from callus by “priming” the expression of key factors (Ishihara *et al*, 2019a). Based on these previous studies and current results, we propose that active demethylation by LDL3, driven by transcriptional elongation, functions as a record of transcriptional activity, which might control developmental plasticity and robust gene control in plants. This concept could be further extended in the future using *Arabidopsis* histone methyltransferase mutants specifically affecting H3K4me2 (Oya *et al*, 2022a). Finally, binding to the elongating transcription machinery has also been reported in the human LDL3 homolog, LSD2, which functions specifically for H3K4me2 (Fang *et al*, 2010). It would be interesting to determine whether a similar strategy has convergently evolved to control diverse functions of H3K4me2 in animals and plants.

## Materials and Methods

### Plant materials and growth condition

*Arabidopsis thaliana* strain Columbia‐0 (Col‐0) was used as wild type (WT). The mutant alleles used in this study were: *ldl3‐1* (GABI_092C03) (Ishihara *et al*, 2019a), *elf7‐2* (SALK_046605) (Tamada *et al*, 2009), *elf7‐3* (SALK_019433) (Cao *et al*, 2008; Li *et al*, 2019), *elf8‐1* (SALK_090130), *elf8‐2* (SALK_065364) (Shiraya *et al*, 2008), *vip3‐1* (SALK_139885) (Fal *et al*, 2017, 2019), *vip4‐4* (SALK_039374) (He *et al*, 2004; Li *et al*, 2019), *vip5‐1* (SALK_062223) (Oh *et al*, 2004), *cdc73‐1* (SALK_150644) (Yu & Michaels, 2010), *cdkf;1–1* (SALK_148336) (Hajheidari *et al*, 2012), *hub1‐4* (SALK_122512) (Cao *et al*, 2008), *hub2‐2* (SALK_071289) (Cao *et al*, 2008), *sdg8* (*ashh2‐1*, SALK065480) (Grini *et al*, 2009), all of which are in the Col‐0 background. Plants were grown on Murashige and Skoog (MS) media supplemented with 1% sucrose and solidified with Bacto agar under long day (16 h light/8 h darkness) photoperiods at 22°C.

To make the pLDL3::LDL3‐3xFLAG fusion construct, an LDL3 genomic DNA fragment (2.5 kb upstream of ATG to just before the stop codon) followed by 3xFLAG sequence was cloned into the pGreenII 0179 vector. The pLDL3::LDL3‐GFP or pLDL3::LDL3ΔSRI‐GFP fusion construct was cloned from an LDL3 genomic DNA fragment (2.5 kb upstream of ATG to just before the stop codon (full length) or to the sequence encoding the 1,538^th^ amino acid) followed by GFP sequence was cloned into the pPLV01 vector. The plasmid was transformed into the *ldl3‐1* mutant via Agrobacterium tumefaciens GV3101::pMP90. In the same way, the pELF8::ELF8‐GFP fusion construct was cloned from an ELF8 genomic DNA fragment (from ~1 kb upstream of the ATG to just before the stop codon) followed by GFP sequence was cloned into the pGreenII 0179 vector. The plasmid was transformed into the *elf8‐2* mutant. FLAG‐tagged LDL2 and FLD plants used in this work were described before (Inagaki *et al*, 2017, 2021).

### ChIP‐seq

Chromatin immunoprecipitation sequencing (ChIP‐seq) was carried out following (Inagaki *et al*, 2017) with modifications. 0.5~1.0 g of aerial parts from 14‐day‐old seedlings were used as each ChIP sample. Samples were ground in liquid nitrogen and crosslinked for 10 min at room temperature in Nuclei isolation buffer (10 mM HEPES pH 7.5, 1 M sucrose, 5 mM KCl, 5 mM MgCl_2_, 5 mM EDTA, 0.1% β‐mercaptoethanol, 0.6% Triton X‐100, supplemented with 1 tablet/50 ml cOmplete proteinase inhibitor and 1 mM Pefabloc SC (Roche)) supplemented with 1% formaldehyde. In the case of the LDL3‐3xFLAG and ELF8‐GFP ChIP shown in Fig 5, the crosslink buffer additionally contains 1.5 mM ethylene glycol bis (succinimidyl succinate) (EGS). In the case of RNAPII ChIP, the formaldehyde concentration was 2%. The crosslinking reaction was stopped with 130 mM glycine. The suspension was filtered through a 40 μm nylon cell strainer and pelleted by centrifugation at 3,000 *g* at 4°C for 10 min. The pellet was resuspended in 300 μl of nuclei isolation buffer and layered on top of 500 μl of nuclei separation buffer (10 mM HEPES pH 7.6, 1 M sucrose, 5 mM KCl, 5 mM MgCl_2_, 5 mM EDTA pH 8.0, 15% Percoll) and pelleted by centrifugation at 16,000 *g* for 5 min at 4°C. The nuclear pellet was resuspended in 950 μl RIPA without Triton buffer (50 mM Tris–HCl pH7.8, 150 mM NaCl, 1 mM EDTA, 0.1% SDS, 0.1% Sodium deoxycholate and cOmplete proteinase inhibitor).

Sonication was conducted using Covaris S2 Focused‐ultrasonicator (Covaris) and milliTUBE 1 ml AFA Fiber (Covaris) with following settings: power mode, frequency sweeping; time, 18–20 min; duty factor, 5%; Cycles per Burst, 200; temperature (water bath), 4–6°C; and Peak Incident Power, 140 for histones and 105 for epitope‐tagged proteins. In the case of RNAPII ChIP, chromatin was sheared using a Picoruptor (Diagenode) (6 cycles of 30 s on and 30 s off).

The sonicated chromatin was then centrifuged at 13,000 *g* for 3 min, and the supernatant was added with 50 μl of 20% Triton X‐100 and aliquoted. The chromatin solution was incubated with 1–2 μg of antibodies overnight at 4°C. Antibodies used are H3K4me1 (ab8895; Abcam), H3K4me2 (ab32356; Abcam), H3K4me3 (ab8580; Abcam), and anti‐H3 (ab1791; Abcam), H3K36me2 (MABI0332; MBL), H3K36me3 (MABI0333; MBL), H2Bub (MM‐0029; Medimabs), RNAP2 total CTD (Diagenode, C15100055), RNAP2 phospho S2 (MABI0602; MBL), RNAP2 phospho S5 (MABI0603; MBL), FLAG (F1804; SIGMA), GFP(ab290; Abcam).

Then the antibody‐chromatin mix was incubated for 2 h at 4°C with magnetic beads; Dynabeads M280 Sheep anti‐mouse IgG in the cases of MBL antibodies, and with Dynabeads Protein G in the cases for the others. Wash, reverse cross‐linking, and DNA extraction were conducted as described previously (Inagaki *et al*, 2021). For epitope‐tagged proteins, instead of LiCl buffer (1% IGEPAL, 1% Sodium deoxycholate), half LiCl buffer (0.5% IGEPAL, 0.5% Sodium deoxycholate) was used. Collected DNA was quantified with the Qubit dsDNA High Sensitivity Assay kit (Thermo Fisher Scientific), and 1–2 ng DNA was used to make libraries for Illumina sequencing. The library was constructed with the KAPA Hyper Prep Kit for Illumina (KAPA Biosystems), and dual size‐selected using SPRI select beads (Beckman Coulter) to enrich 300–500‐bp fragments. The libraries were 50‐bp single‐end sequenced by HiSeq4000 sequencer (Illumina) in Vincent J. Coates Genomics Sequencing Laboratory at UC Berkeley, or 150 bp paired‐end sequenced by the HiSeqX Ten sequencer (Illumina). Biological replicates were conducted on independently grown plants.

### eChIP‐seq

In the experiments shown in Fig 5D and Appendix Fig S6, the eChIP method (Zhao *et al*, 2020), which can be performed with a small amount of plant material, was performed because many mutants that were used in the experiment showed sterility and it was difficult to collect a large amount of material. The results were comparable to those of conventional methods.

0.01~0.1 g of aerial parts from 14‐day‐old seedlings were ground in liquid nitrogen and added to Fixation Buffer (phosphate‐buffered saline (PBS) with 1% formaldehyde, 1 mM Pefabloc SC (Roche) and cOmplete protease inhibitor cocktail (Roche))and incubated at room temperature for 10 min. Glycine was added to a concentration of 0.2 M and further incubated at room temperature for 5 min, after which the supernatant was removed by centrifugation. Pellets were lysed in 180 μl of Buffer S (50 mM HEPES‐KOH (pH 7.5), 150 mM NaCl, 1 mM EDTA, 1% Triton X‐100, 0.1% sodium deoxycholate, 1% SDS) for 10 min at 4°C. The homogenate was mixed with 720 μl of Buffer F (50 mM HEPES‐KOH (pH 7.5), 150 mM NaCl, 1 mM EDTA, 1% Triton X‐100, 0.1% sodium deoxycholate). The chromatin was fragmented into 200~600 bp by sonication using Picoruptor (diagenode). The rest of the procedure was the same as in Zhao *et al* (2020). The antibodies used were H3: ab1791 (Abcam) and H3K4me2: ab7766 (Abcam).

### mRNA‐seq

Total RNA was isolated from the aerial part of one 14‐day‐old seedling grown on MS media, using the RNeasy Plant Mini Kit (Qiagen), and treated with DNase I (Takara). Libraries for mRNA‐seq were constructed using the KAPA Stranded RNA‐seq Library Preparation Kit according to the manufacturer's instructions. First, poly(A) RNA was purified and then fragmented by heating purified RNA at 94°C for 7 min in the fragment, prime, and elute buffer. Then, double‐strand cDNA was synthesized, and adapter‐ligated libraries were constructed. Two independent biological replicates were analyzed for each genotype.

### chrRNA‐seq

First, nuclei were extracted from approximately 0.5 g of aerial parts from 14‐day‐old seedlings grown on MS media following the protocol as described previously (Inagaki *et al*, 2021) with the following modifications. The isolated nuclei were resuspended in 500 μl of NUN1 buffer (20 mM Tris–HCl, pH 8.0, 75 mM NaCl, 0.5 mM EDTA, 50% 7glycerol, and 1× cOmplete EDTA‐free Protease Inhibitor Cocktail) followed by 500 μl of NUN2 buffer (20 mM HEPES‐KOH pH 7.6, 7.5 mM MgCl_2_, 0.2 mM EDTA, 300 mM NaCl, 1 M urea, 1% NP40, cOmplete EDTA‐free Protease Inhibitor Cocktail). The solution was incubated at 4°C for 15 min, with vortexing every 3 min. The chromatin pellet was precipitated by performing 15,000 *g* centrifuge at 4°C for 3 min. The chromatin pellet was resuspended in 100 μl of lysis buffer (10 mM Tris–HCl, pH 7.8, 10 mM EDTA, 0.5% SDS). These samples were used for chrRNA‐seq and RNAPII Western blotting. chrRNA was extracted using miRNeasy kit (Qiagen). Ribosomal RNA was depleted and the remaining DNA was depleted using the KAPA RiboErase kit. The chrRNA‐seq libraries were constructed from 50 ng of purified RNA using the KAPA Stranded mRNA‐seq Kit, skipping the poly‐A selection step and performing the RNA fragmentation step at 94°C for 5 min. Two biological replicates were analyzed. 150‐bp paired‐read sequences were obtained using the HiSeq X Ten sequencer (Illumina) at Macrogen Japan Corp.

### Data analysis

The mRNA‐seq, ChIP–seq, and chrRNA‐seq data were processed as described in (Inagaki *et al*, 2021). Heatmaps of enrichment were generated using the computeMatrix and plotHeatmap utilities in deepTools version 3.5.1 (Ramírez *et al*, 2016).

For the random forest analysis, each gene was divided into the TSS region, the TTS region and the gene body. The TSS (or TTS) region here was defined as 200 bp upstream and downstream from the TSS (or TTS) combined, and the gene body was defined as the region spanning the TSS to the TTS. Short genes (< 400 bp) were omitted. The objective variable was defined as follows: Hyper‐H3K4me2 genes in the *ldl3* mutant are genes with [RPKM of H3K4me2 in *ldl3*] − [RPKM of H3K4me2 in WT] > 2, which are defined as ‘LDL3 target genes’ (*n* = 7,367), and the others are ‘non‐target’. The numbers of LDL3 target and non‐target genes were balanced by randomly choosing 7,367 genes from non‐target genes. The data frame for the predictors was previously described (Inagaki *et al*, 2021) (https://github.com/soinagak/FLD2021). The RNAPII Ser2P ratio and Ser5P ratio were added to the predictor variables of this data frame.

The random forest was trained with genes on chromosomes 1–4. Cross‐validation was performed with genes on chromosome 5. R package randomForest (https://www.rdocumentation.org/packages/randomForest/versions/4.6‐14/topics/randomForest) was used with option ntree = 1,000. The ROC curve was plotted using R package ROCR (Sing *et al*, 2005).

### Co‐immunoprecipitation (Co‐IP)

The Co‐IP experiments were performed as described in Pfab *et al* (2017) with minor modifications. Two grams of 14‐day‐old seedlings were used for a Co‐IP. The sample was ground into fine powder and suspended in 10 ml of Extraction buffer (25 mM HEPES‐KOH pH 7.4, 100 mM NaCl, 2 mM MgCl_2_, 10% glycerol, 0.05% IGEPAL CA‐630, 1 mM DTT, 5 mM EGTA, 1× cOmplete proteinase inhibitor). MgCl_2_ was added to a final concentration of 5 mM and 50 U/ml benzonase was added to the mixture. The mixture was incubated for 30 min at 4°C on a rotating wheel and pelleted by centrifugation at 4,000 *g* for 10 min at 4°C. The supernatant was filtered through a 40 μm nylon cell strainer on ice. IP was performed from the supernatant using FLAG antibody (or GFP antibody)‐conjugated beads or only beads (mock) for 1 h. The beads were washed with 1 ml of ice‐cold Extraction buffer three times.

### Western blotting

Bulk histones for Western blotting were performed as described before (Oya *et al*, 2022a). RNAPII samples for Western blotting were prepared as described in the method for chrRNA‐seq. 50 μl of chromatin suspension were mixed with an equal volume of 2× SDS buffer and boiled for 5 min at 95°C.

Proteins were resolved on SDS–PAGE and transferred to a PVDF membrane using the Trans‐Blot Turbo and Trans‐Blot transfer pack (Bio‐Rad). The primary and secondary antibody reactions were performed using an iBind Flex Western System (Thermo Fisher Scientific). Primary antibody (H3K4me1 (#710795; Invitrogen), H3K4me2 (ab7766; Abcam), H3K4me3 (ab8580; Abcam), H3 (ab1791; Abcam), RNAPII total CTD (Diagenode, C15100055), RNAPII phospho S2 (MABI0602; MBL), RNAPII phospho S5 (MABI0603; MBL), FLAG (F1804; SIGMA)) and secondary antibody (Anti‐Rabbit IgG HRP (NA934; Cytiva), Anti‐mouse IgG HRP (NA931, Cytiva)) reactions were performed at room temperature for more than 2.5 h according to the manufacturer's protocol. The blots were developed with ECL prime solution (Cytiva), and the signal was quantified by iBright Imager (Thermo Fisher Scientific).

### RT–qPCR

Total RNA was isolated from aerial parts of three 14‐day‐old seedlings grown on MS media, using the RNeasy Plant Mini Kit (Qiagen) and cDNA was synthesized using ReverTra Ace qPCR RT Master Mix with gDNA Remover (TOYOBO). Real‐time PCR was performed using LightCycler 96 (Roche) and THUNDERBIRD SYBR qPCR mix (Toyobo) with 50 cycles of denaturation at 95°C for 15 s and extension at 60 °C for 1 min using specific primers for LDL3 (forward: TCCTAGTTGGAAGCATGACCAACGAGCG reverse: GAGTCGAGGAACTGTGTAACAGCCATAGC), and for 60S ribosomal protein gene AT3G49010 (forward: TCGCAAGAACCGATCTTTGGAGG, reverse: AACTCTTCTGGTGTAGAGTCACC) as a reference. The plant tissues for gene expression analysis were produced in three biological replicates and three technical replicates of each repetition were carried out.

## Author contributions

**Tetsuji Kakutani:** Conceptualization; supervision; funding acquisition; writing – review and editing. **Shusei Mori:** Conceptualization; formal analysis; investigation; writing – original draft; writing – review and editing. **Satoyo Oya:** Conceptualization; resources; investigation; writing – review and editing. **Mayumi Takahashi:** Investigation. **Kazuya Takashima:** Investigation. **Soichi Inagaki:** Conceptualization; resources; supervision; funding acquisition; writing – review and editing.

## Disclosure and competing interests statement

The authors declare that they have no conflict of interest.

## Supporting information



Appendix

Source Data for Figure 2

Source Data for Figure 3

Source Data for Figure 4

Source Data for Figure 5

Source Data for Figure 6

## Data Availability

The high‐throughput sequencing data generated in this study is available in the NCBI database under the accession number PRJNA934737 (https://www.ncbi.nlm.nih.gov/bioproject/PRJNA934737).
