# Supplementary material for: Cotranscriptional demethylation induces global loss of H3K4me2 from active genes in Arabidopsis
Source: EMBO J. 2023 Oct 18;42(23):e113798. doi: 10.15252/embj.2023113798 (PMC10690457; doi:10.15252/embj.2023113798)
Supplement: Supplementary file 1 — Appendix [file EMBJ-42-e113798-s005.pdf]

Cotranscriptional demethylation induces global loss of H3K4me2 from active genes in *Arabidopsis*

Shusei Mori[1*], Satoyo Oya[1], Mayumi Takahashi[2], Kazuya Takashima[2], Soichi Inagaki[1*], Tetsuji Kakutani[1,2*]

Appendix Table of Content

Appendix Fig. S1……………………………………2
Appendix Fig. S2……………………………………3
Appendix Fig. S3……………………………………4
Appendix Fig. S4……………………………………5
Appendix Fig. S5……………………………………6
Appendix Fig. S6……………………………………8
Appendix Fig. S7……………………………………10
Appendix Fig. S8……………………………………11
Appendix Fig. S9……………………………………14
Appendix Fig. S10…………………………………...15
Appendix Fig. S11…………………………………...16
Appendix Fig. S12…………………………………...17
Appendix Fig. S13…………………………………...19
Appendix Fig. S14…………………………………...21
Appendix Fig. S15…………………………………...22
Appendix Fig. S16…………………………………...23
Appendix Fig. S17…………………………………...25
Appendix Fig. S18…………………………………...27
Appendix Fig. S19…………………………………...28

Appendix Figure S1

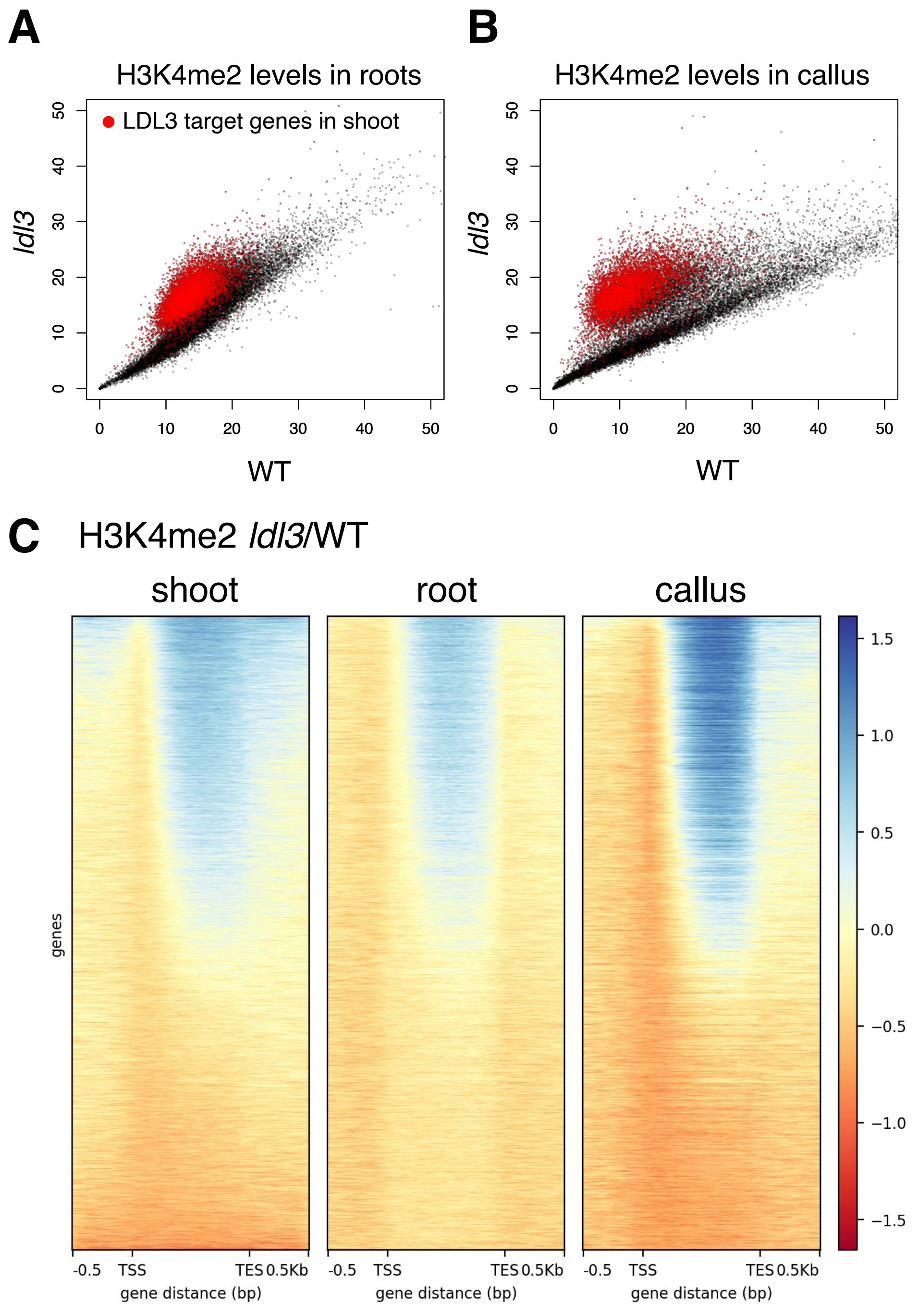

Appendix Figure S1. LDL3 has the same demethylation target genes across several tissues.
(A, B) H3K4me2 levels in roots (A) and callus (B) of the *ldl3* mutants compared with WT (Data ref: Ishihara et al, 2019). Each dot represents RPKM within each protein-coding gene. Red dots, protein-coding genes with hyper-H3K4me2 in shoot tissues of *ldl3* (7,367 genes; Fig. 1A). (C) Heatmaps depicting changes in H3K4me2 levels in the *ldl3* mutants compared with WT in each tissue. The protein-coding genes were sorted based on increased gene-body H3K4me2 levels in *ldl3* in shoot.

Appendix Figure S2

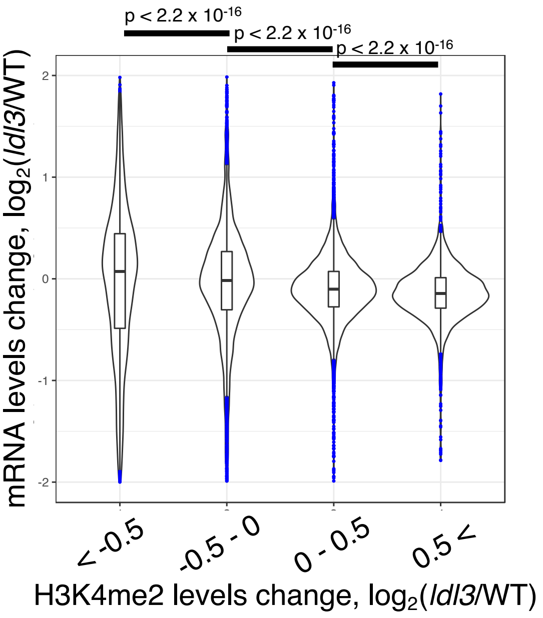

Appendix Figure S2. H3K4me2 negatively affects transcription.
Violin plots and box plots comparing the expression changes (mRNA RPKM) of protein-coding genes. Genes are divided according to the H3K4me2 levels (RPKM) changes. Genes for which no transcription was detected were excluded. The central line corresponds to the median; the lower and upper bounds of the box correspond to the first and third quartiles, respectively; the whiskers indicate the data range within the 1.5 × interquartile range (IQR); and the blue dots indicate the outliers. The P value is based on a Mann-whitney-wilcoxon test.

Appendix Figure S3

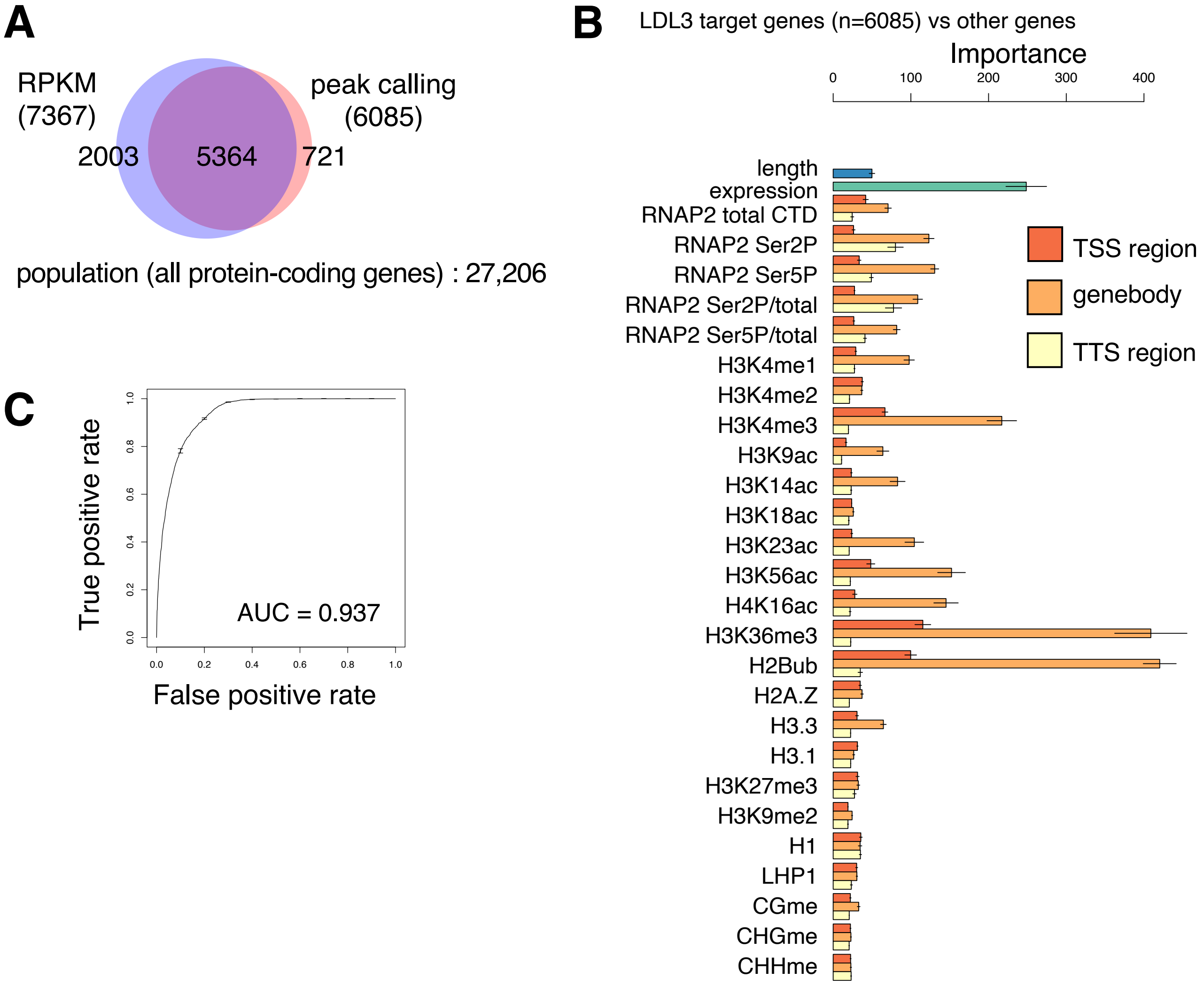

Appendix Figure S3. Identification of LDL3 target genes by peak calling.

(A) Venn diagram of the genes with hyper-H3K4me2 in *ldl3* identified by two methods (RPKM: H3K4me2 levels in *ldl3* (RPKM) - H3K4me2 levels in WT (RPKM) > 2; 7,367 genes) (peak calling: identified by comparison of ChIP profiles for H3K4me2 in *ldl3* and WT (see methods; q < 0.1); 6,085 genes). (B) Chromatin features predictive of the genes that gain H3K4me2 in the *ldl3* mutant identified by peak calling method in the random forest models. Error bars represent the standard deviation of the five repeats of training. (C) ROC plot showing the prediction accuracy of the random forest models.

Appendix Figure S4

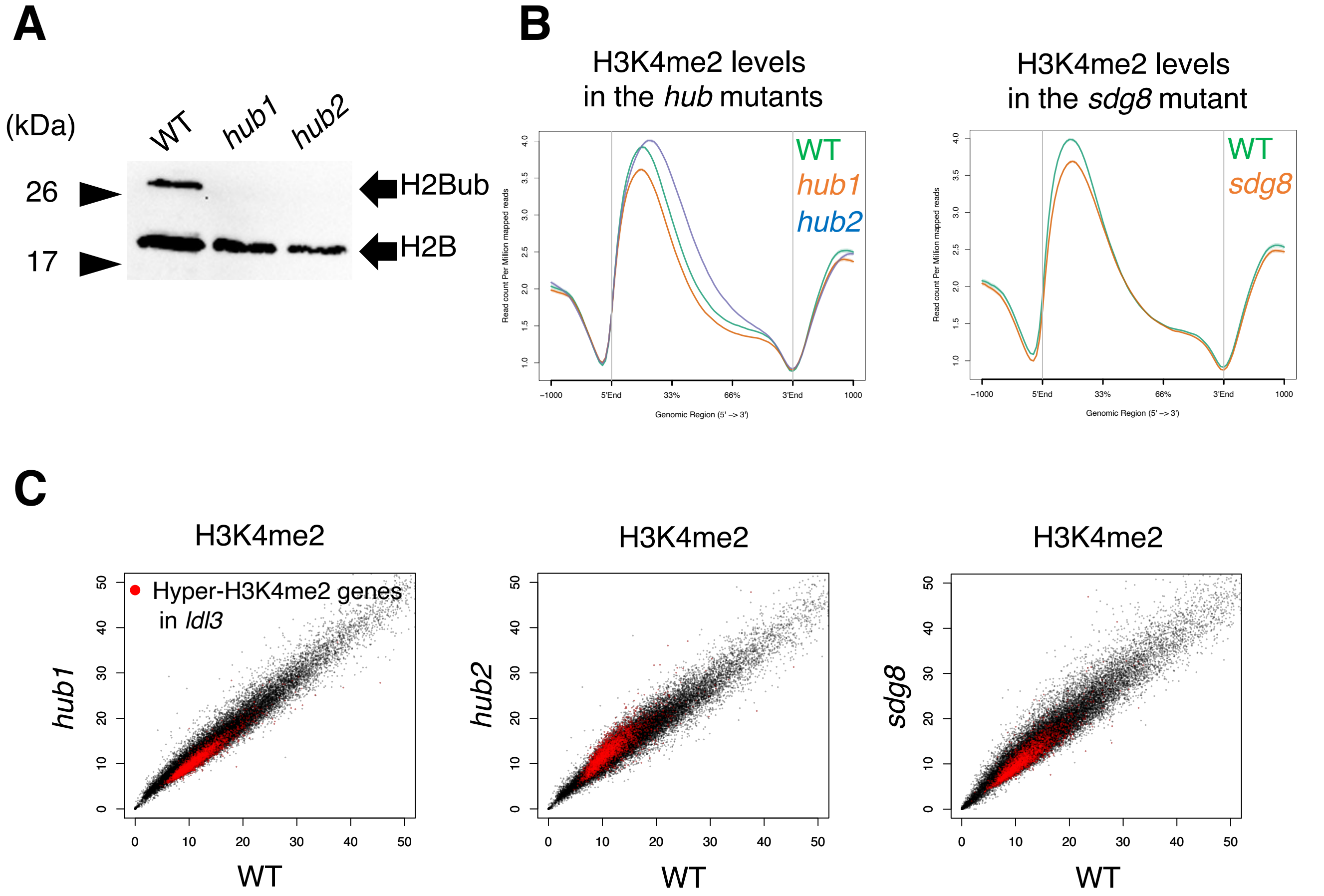

Appendix Figure S4. H2Bub and H3K36me3 have little effect on the H3K4me2 pattern.

(A) Western blotting of H2B and H2Bub on bulk histone extracted from the *hub* mutants. (B) Averaged profiles of H3K4me2 around LDL3 target genes in each genotype (WT, *hub1*, *hub2*, *sdg8*). The numbers of genes analyzed are 7,367 as in Fig. 2A. The ribbons indicate s.e.m. (C) H3K4me2 levels in each of the mutants compared with WT. Each dot represents RPKM within each protein-coding gene. Red dots, protein-coding genes with hyper-H3K4me2 in *ldl3* (2,809 genes; Fig. 2C). Although the no obvious effect of loss of H2Bub on H3K4me2 is striking contrast to the complete dependency of H3K4me on H2Bub in yeast and animals (Shilatifard et al., 2006), previous reports that performed western blotting for H3K4me2/3 in *hub1/2* mutants of *Arabidopsis* also showed overall levels of H3K4me2/3 were unaffected (Dhawan et al. 2009; Zhao et al., 2019), which corroborate our ChIP-seq results.

Appendix Figure S5

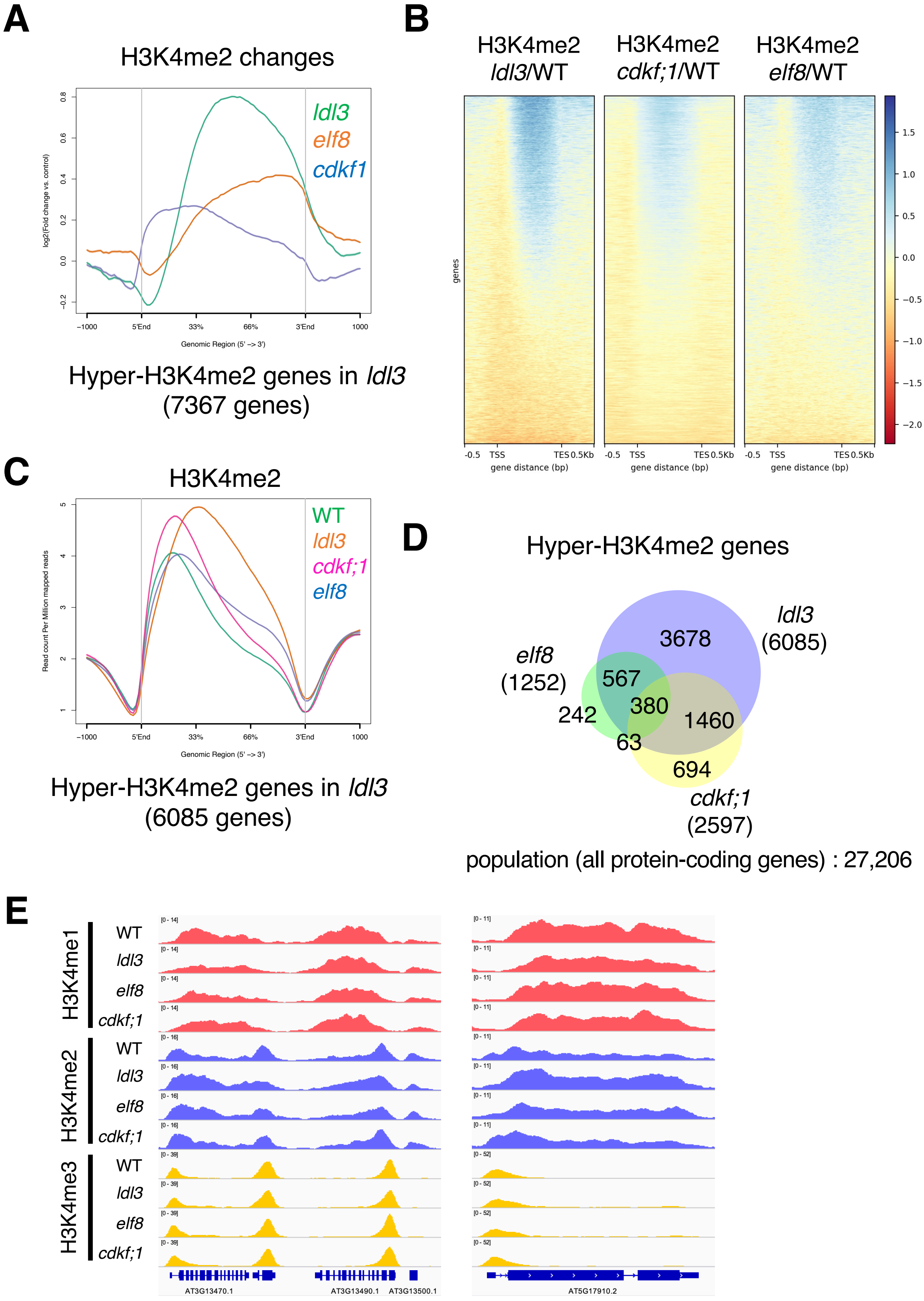

Appendix Figure S5. Defects in RNAPII phosphorylation and transcriptional elongation mimic loss of LDL3 function.

(A) Averaged profiles of H3K4me2 changes around LDL3 target genes in the mutants compared to WT. The numbers of genes analyzed are 7,367 as in Fig. 2A. The ribbons indicate s.e.m. (B) A biological replicate of the experiment shown in Fig. 2B. The protein-coding genes were sorted in the same way as Fig. 2B. (C) Averaged profiles of H3K4me2 around LDL3 target genes identified by peak calling method in each genotype (WT, *ldl3, elf8, cdkf;1*). The ribbons indicate s.e.m. (D) Venn diagram of the genes with hyper-H3K4me2 in each genotype identified by peak calling. (E) Screenshots of H3K4me1/me2/me3 ChIP-seq signals in putative LDL3 target genes.

Appendix Figure S6

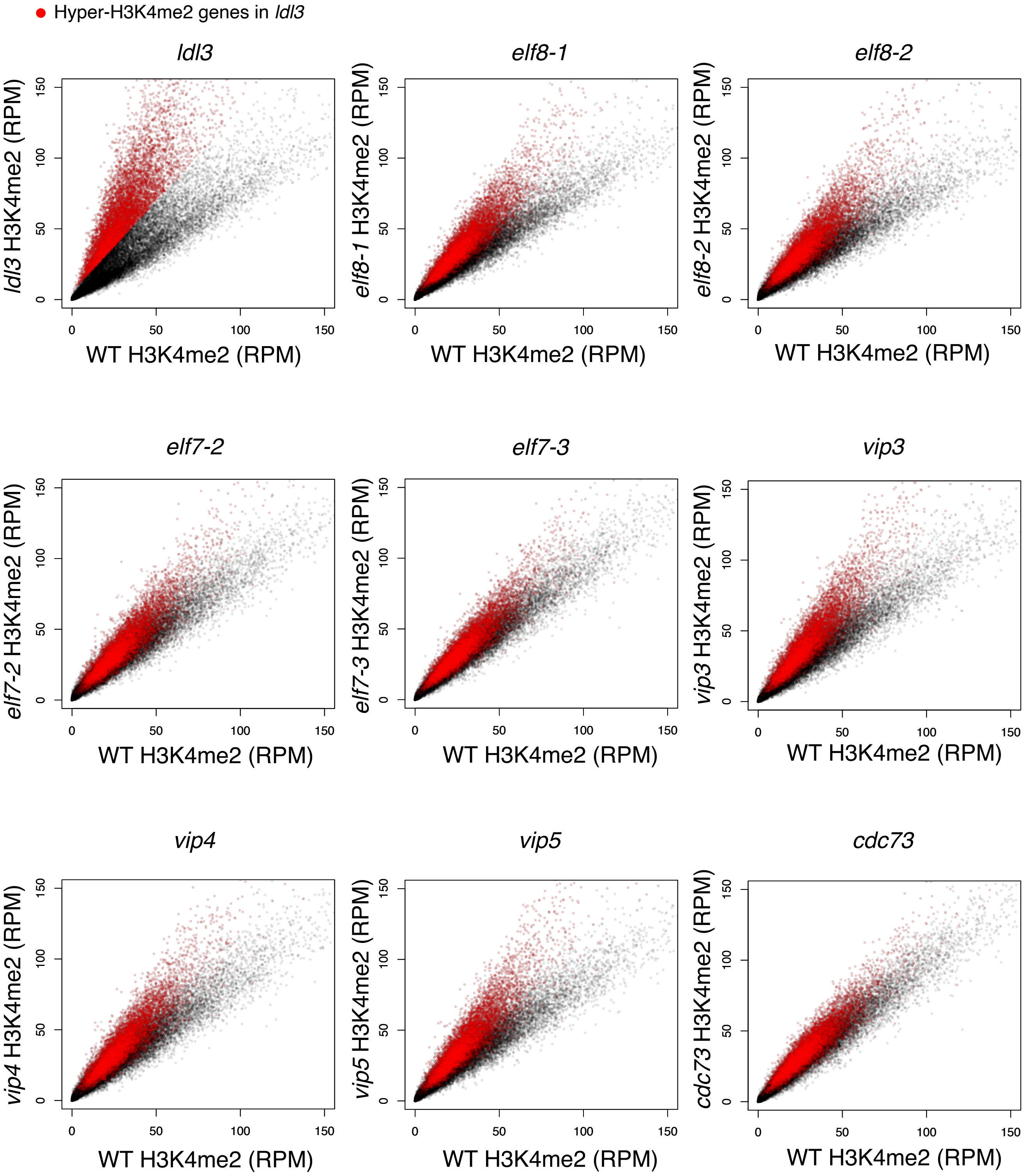

Appendix Figure S6. Paf1C component mutant plants showed increases of H3K4me2 in genes demethylated by LDL3.

H3K4me2 levels in each mutant compared with WT. Each dot represents the RPM within each protein-coding gene. Red dots, protein-coding genes with hyper-H3K4me2 in *ldl3* (H3K4me2 levels in *ldl3* (RPM) - H3K4me2 levels in WT (RPM) > 5; 8,967 genes). The effects of mutations in Paf1C components on the phenotype and H3K4me2 levels appeared to be correlated (Tamada et al, 2009; Yu & Michaels, 2010).

Appendix Figure S7

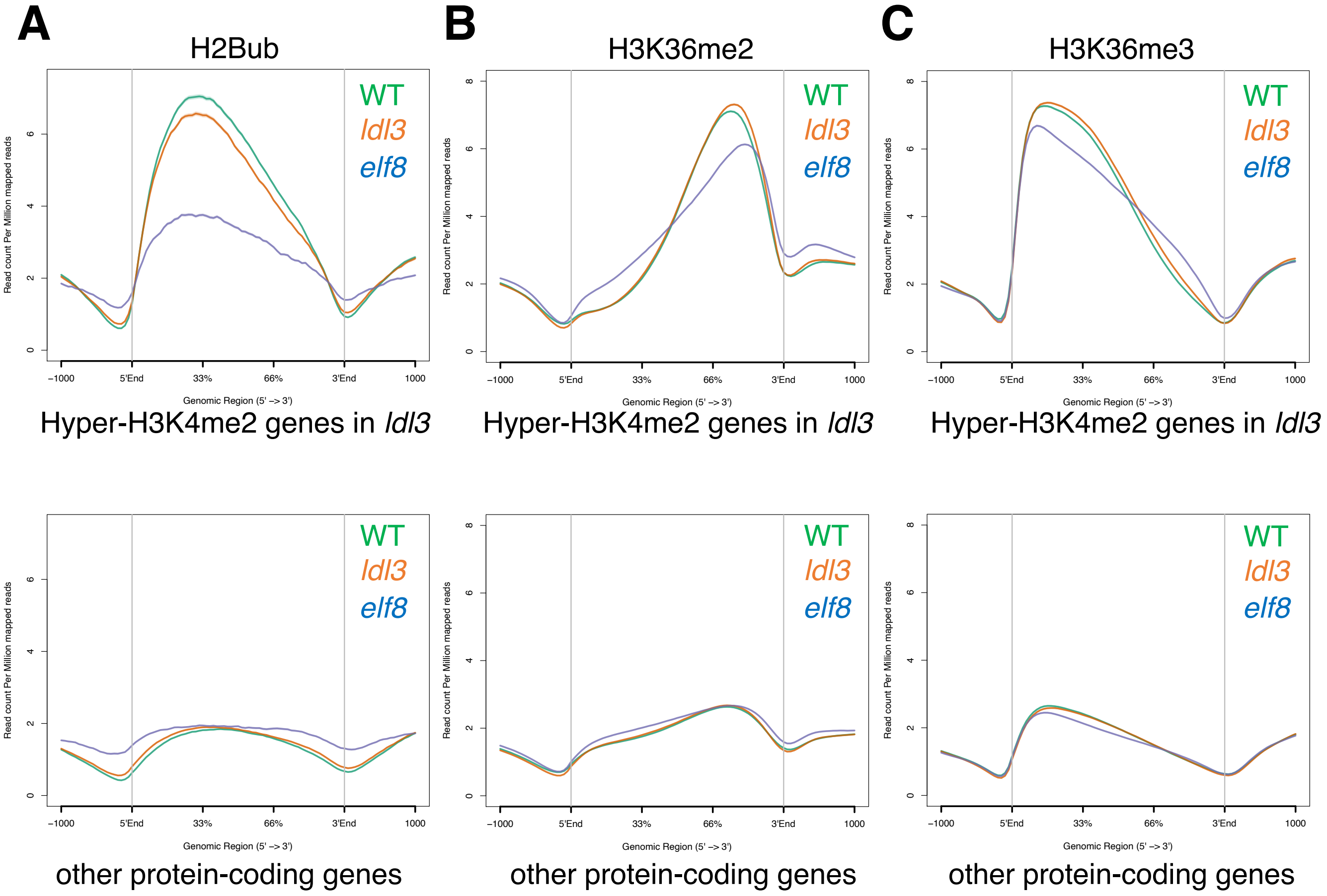

Appendix Figure S7. H2Bub and H3K26me2/me3 are controlled downstream of Paf1C.

(A-C) Averaged profiles of H2Bub (A), H3K36me2 (B) and H3K36me3 (C) around LDL3 target genes (n=7367; Fig. 2A) and the other protein-coding genes (n=19,839) in each genotype (WT, *ldl3*, *elf8*). The ribbons indicate s.e.m.

Appendix Figure S8

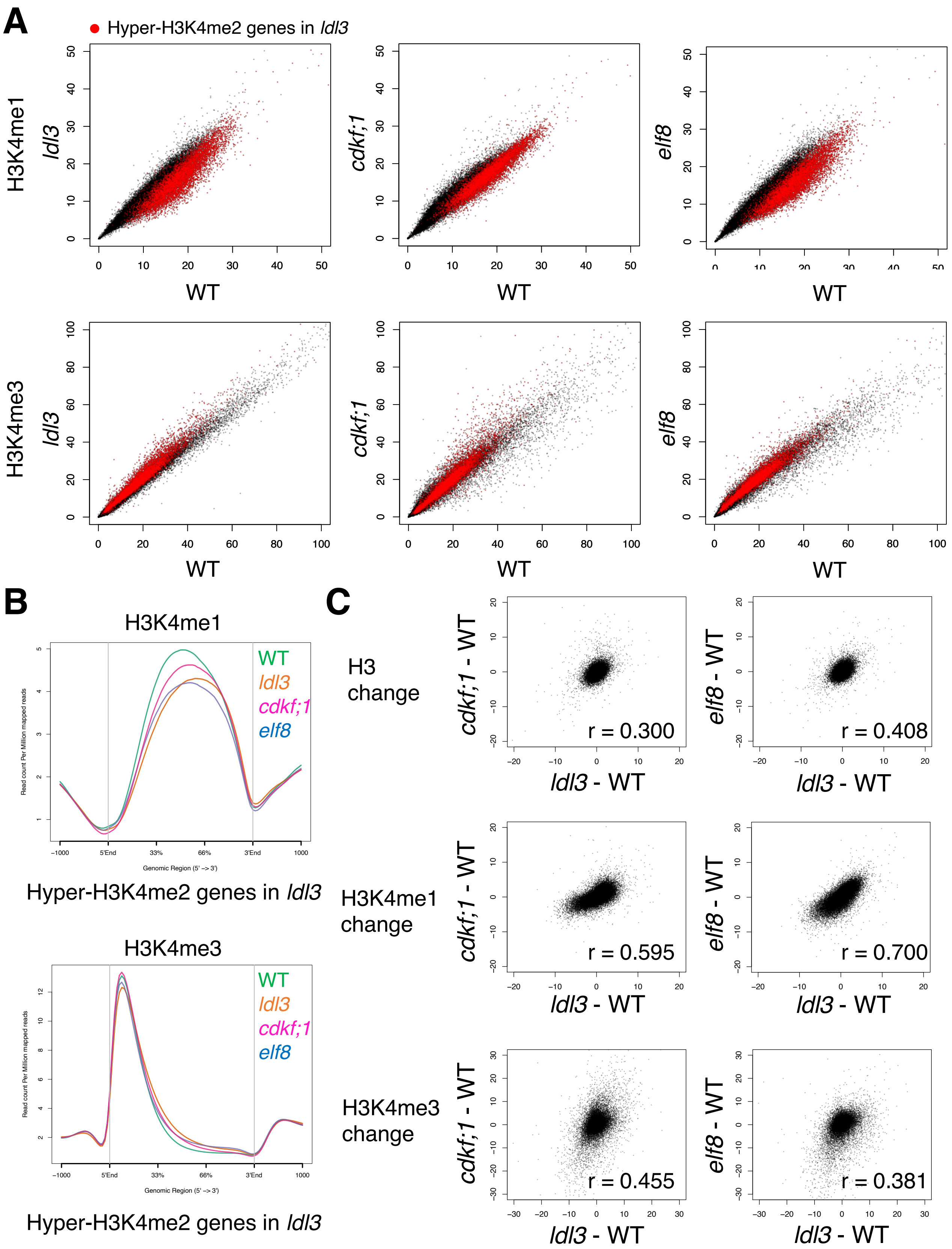

D

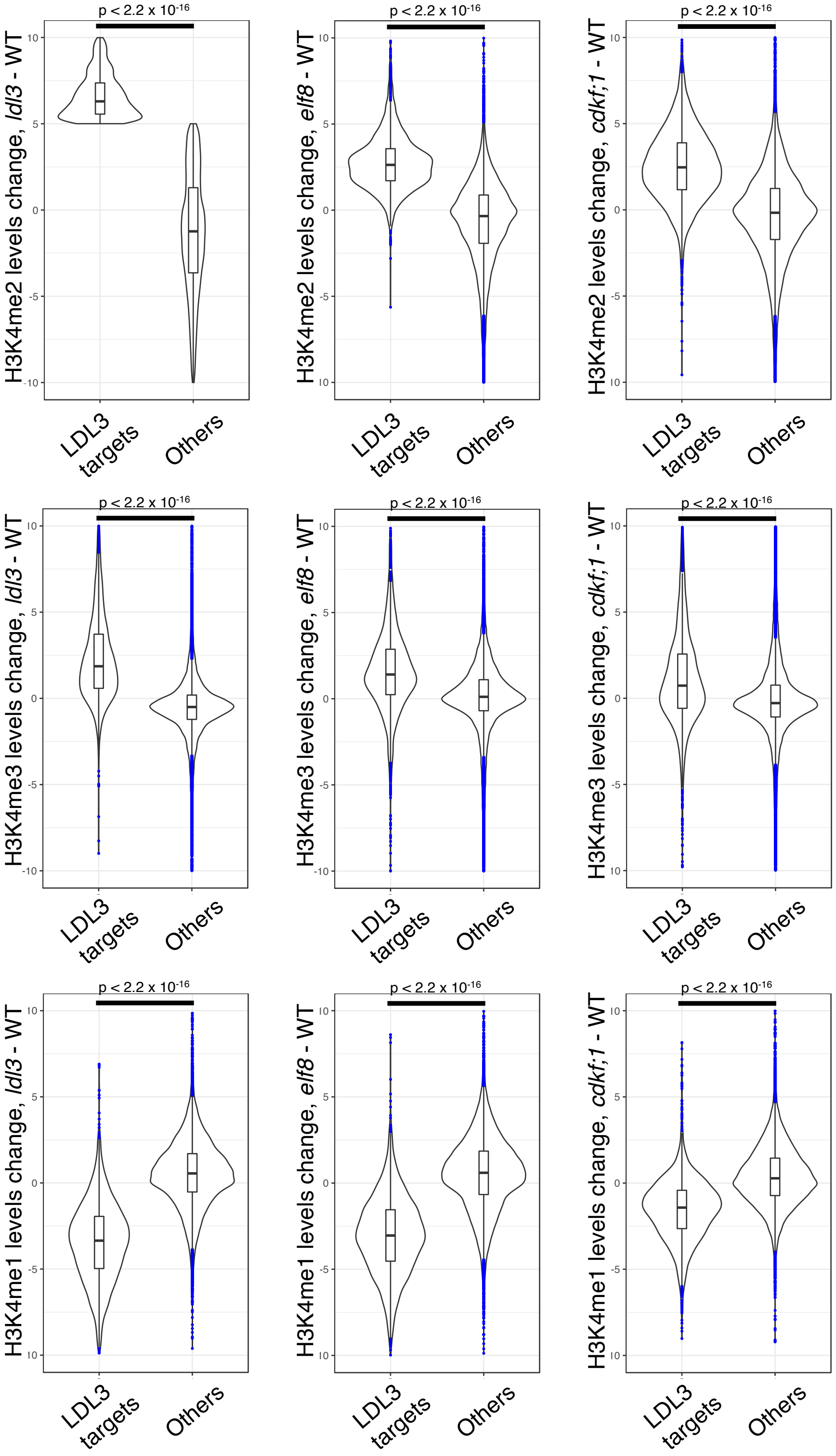
p < 2.2 x 10-16
H3K4me2 levels change, ldl3 - WT
H3K4me2 levels change, elf8 - WT
H3K4me2 levels change, cdkf;1 - WT
H3K4me3 levels change, ldl3 - WT
H3K4me3 levels change, elf8 - WT
H3K4me3 levels change, cdkf;1 - WT
H3K4me1 levels change, ldl3 - WT
H3K4me1 levels change, elf8 - WT
H3K4me1 levels change, cdkf;1 - WT
LDL3 targets
Others

E

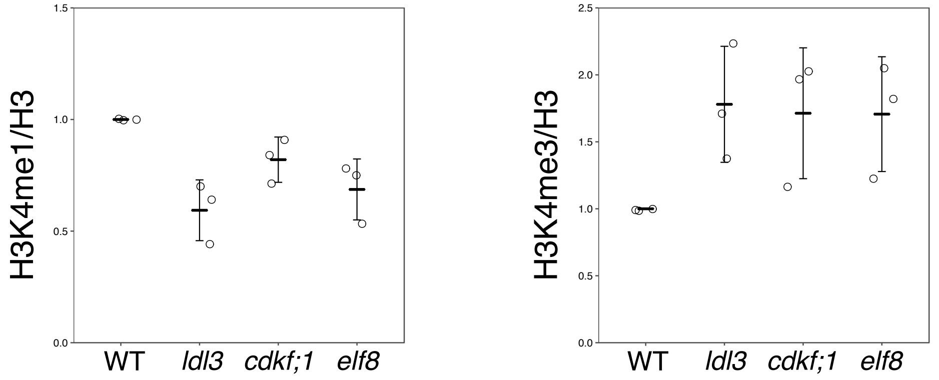

F

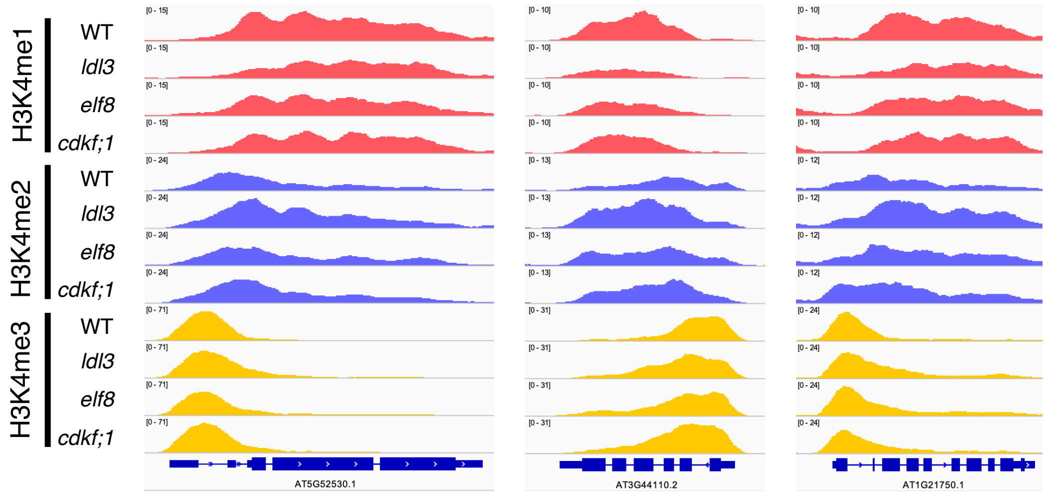

Appendix Figure S8. H3K4me1/me3 in *ldl3*, *elf8*, and *cdkf;1* mutants.

(A) H3K4me1/me3 levels in each of the mutants compared with WT. Each dot represents the RPKM within each protein-coding gene. Red dots, protein-coding genes with hyper-H3K4me2 in *ldl3* (7,367 genes; Fig. 2A). (B) Averaged profiles of H3K4me1/me3 around LDL3 target genes (n = 7367; Fig. 2A) in each genotype (WT, *ldl3*, *elf8*, *cdkf;1*). The ribbons indicate s.e.m. (C) Relationships between changes in H3, H3K4me1, and H3K4me3 levels (RPKM) in *elf8* and *cdkf;1*, and in *ldl3* within each protein-coding gene compared to WT. (D) Violin plots and box plots comparing the H3K4me1/2/3 RPKM changes between LDL3 target genes (n=2,809) and the other genes (n=23,874). Genes are divided according to the H3K4me2 levels changes. The central line corresponds to the median; the lower and upper bounds of the box correspond to the first and third quartiles, respectively; the whiskers indicate the data range within the 1.5 × interquartile range (IQR); and the blue dots indicate the outliers. The P value is based on a Mann-whitney-wilcoxon test. (E) Western blotting of H3K4me1 (left) and H3K4me3 (right) on bulk histone extracted from WT and the mutants. The ratios (H3K4me1/H3 or H3K4me3/H3) of quantified western blotting signals are shown as the relative value to WT. Data represents the mean and standard deviation (n=3). (F) Screenshots of H3K4me1/me2/me3 ChIP-seq signals in putative LDL3 target genes.

Appendix Figure S9

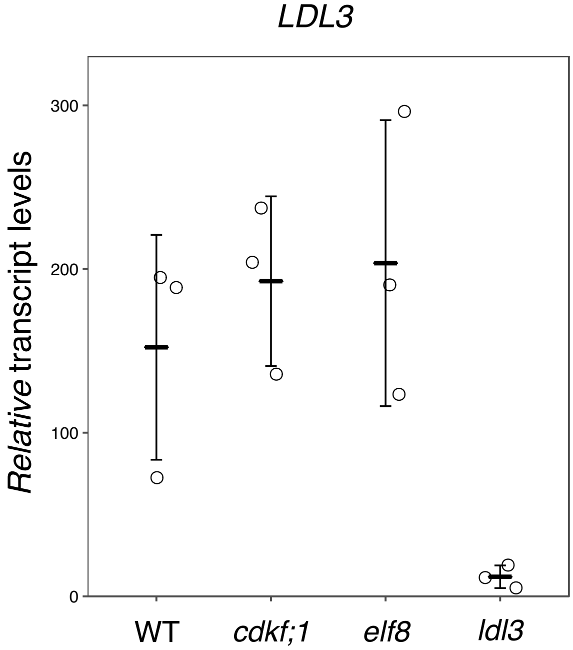

Appendix Figure S9. Paf1C and CDKF;1 do not largely affect the transcript level of LDL3.

mRNA level of *LDL3* gene in WT, *ldl3*, *cdkf;1*, and *elf8*. Relative transcript level determined with RT-qPCR was normalized to the internal control (60 S ribosomal protein coding gene). Means and SD for three biological replicates are shown.

Appendix Figure S10

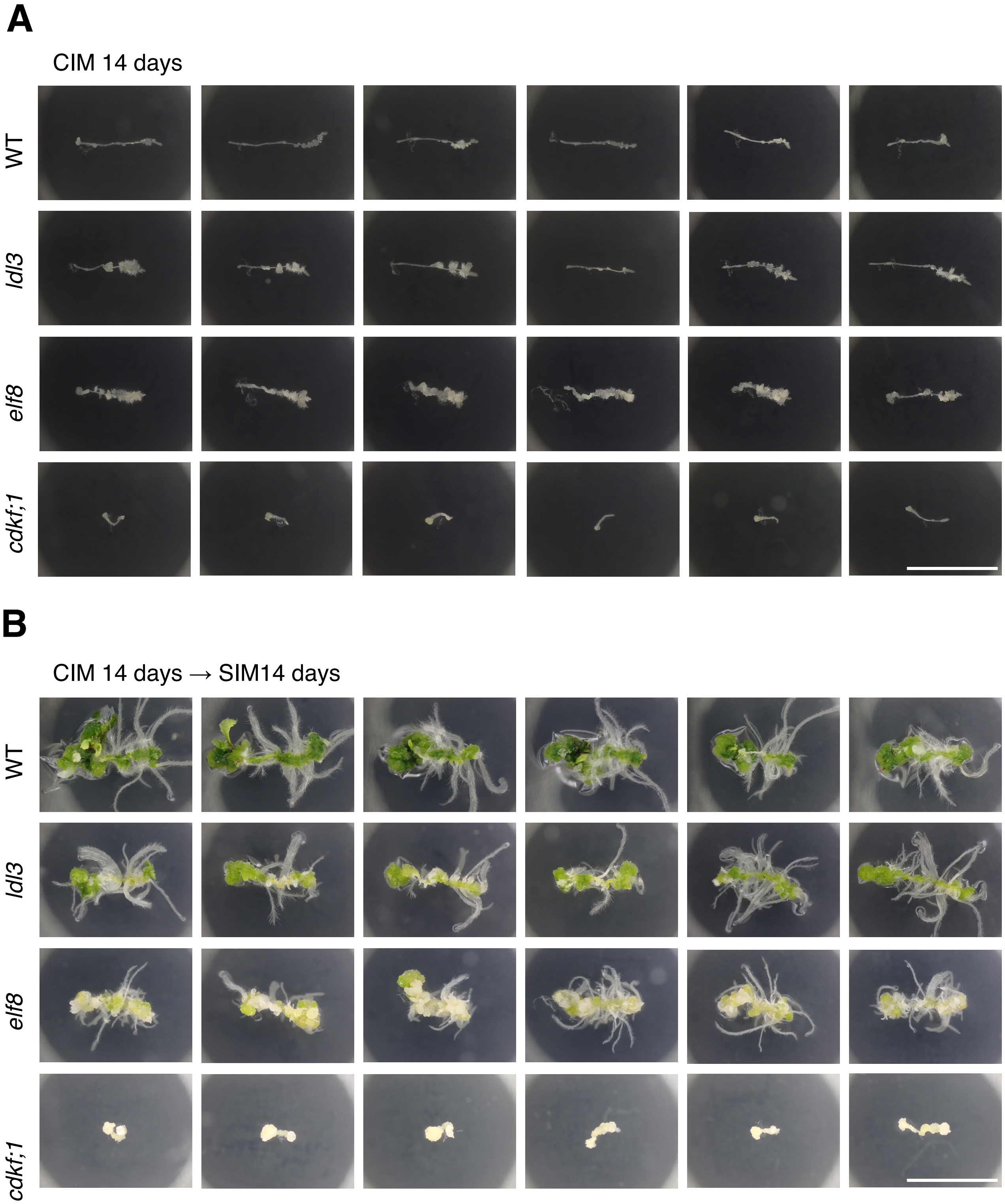

Appendix Figure S10. The *elf8* and *cdkf;1* mutants compromise shoot regeneration from callus similar to the *ldl3* mutant.

(A, B) Shoot regeneration phenotype in each genotype (WT, *ldl3*, *elf8*, *cdkf;1*). The shoot regeneration rate was reduced in *ldl3*, *elf8*, *cdkf;1*. Root tip explants were incubated on CIM 14 days (A), and on SIM 14 days (B). Scale bar: 10 mm

Appendix Figure S11

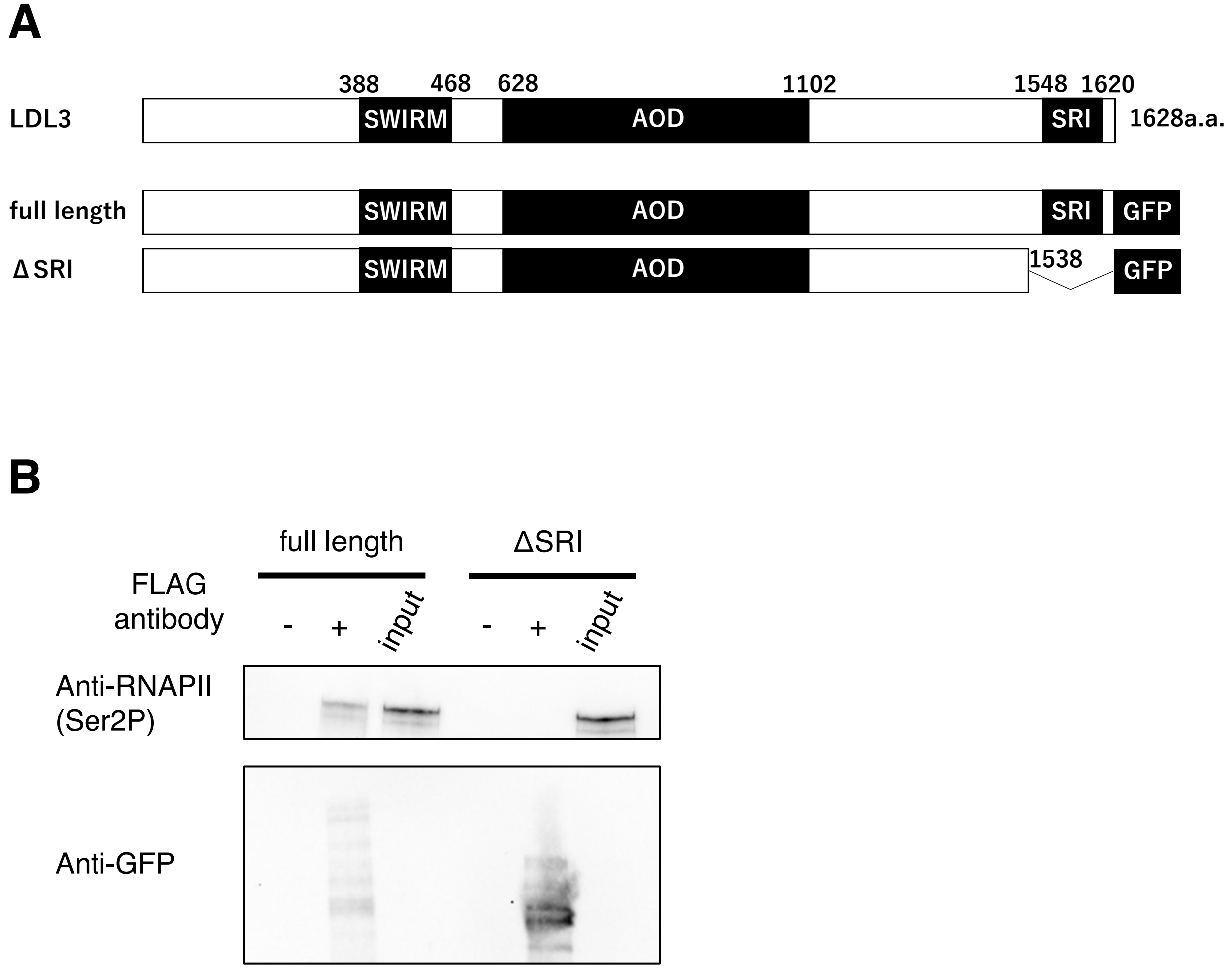

Appendix Figure S11. The SRI domain of LDL3 is responsible for the binding to phosphorylated RNAPII.

(A) Structure of the LDL3 protein and the LDL3 constructs used for Co-IP analysis. SWIRM, predicted chromatin binding domain; AOD, amine oxidase domain including demethylase catalytic center; SRI, the SRI domain are shown. All constructs contained a C-terminal GFP epitope. (B) Co-IP experiments testing the binding between RNAPII (Ser2P), and LDL3 full length and LDL3ΔSRI.

Appendix Figure S12

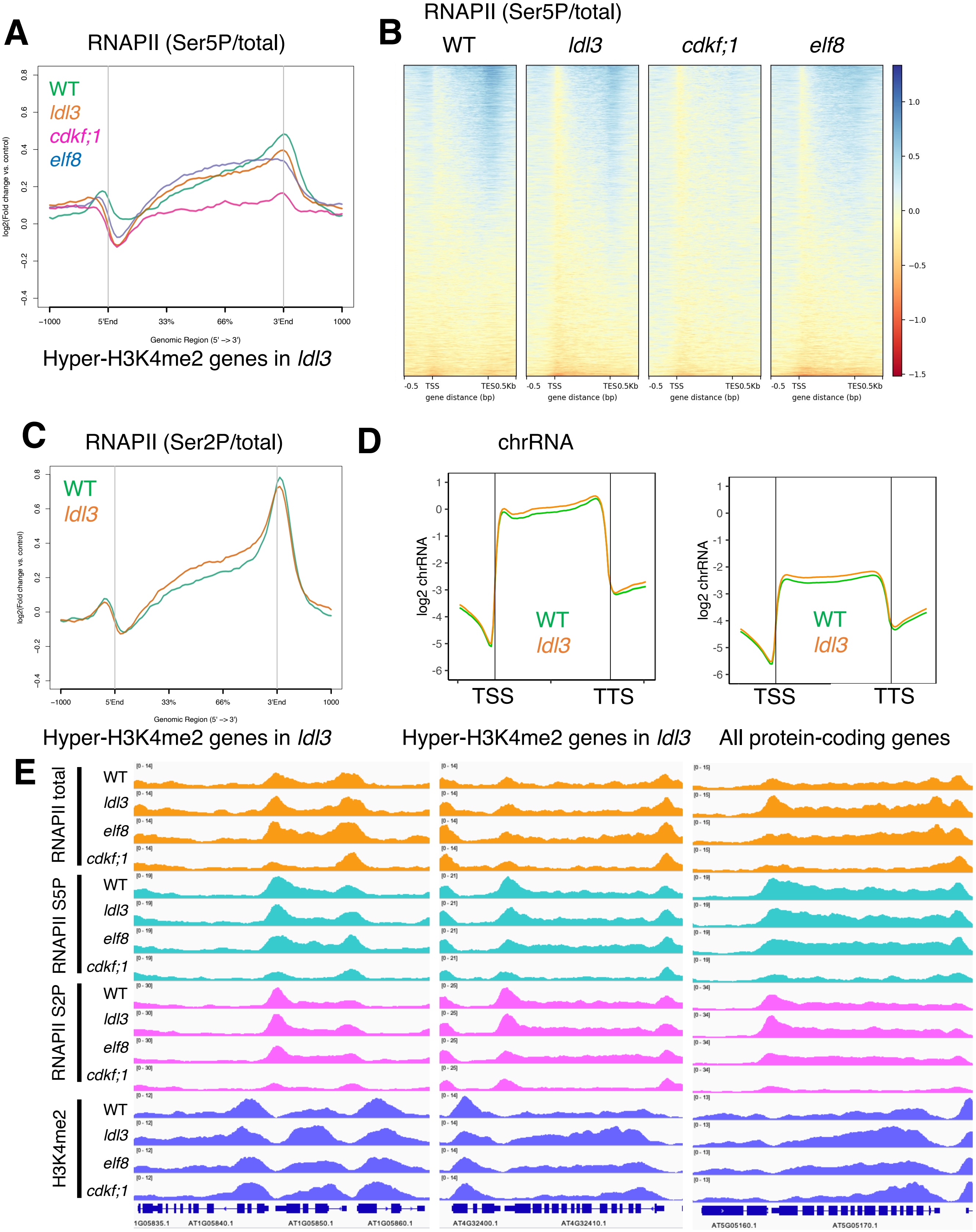

Appendix Figure S12. RNAPII dynamics was not largely affected in the *ldl3* mutant.

(A) Averaged profiles of RNAPII (Ser5P/total CTD ratio) in the gene body around LDL3 target genes (n = 7367; Fig. 2A) for WT, *ldl3*, *elf8*, and *cdkf;1*. The ribbons indicate s.e.m. (B) Heatmaps of RNAPII Ser5P levels (Ser5P/total CTD ratio) are shown in WT and the three mutants. The protein-coding genes were sorted based on increases in gene-body H3K4me2 levels in *ldl3*. (C) Averaged profiles of RNAPII (Ser5P/total CTD ratio) in the gene body around LDL3 target genes for WT and *ldl3*. The ribbons indicate s.e.m. (D) Averaged profiles of chrRNA around LDL3 target genes (n=7367; left) and all protein-coding genes (n=27,206; right) for WT and *ldl3*. The ribbons indicate s.e.m. (E) Screenshots of RNAPII (total, S5P, S2P), H3K4me2 ChIP-seq signals in putative LDL3 target genes.

Appendix Figure S13

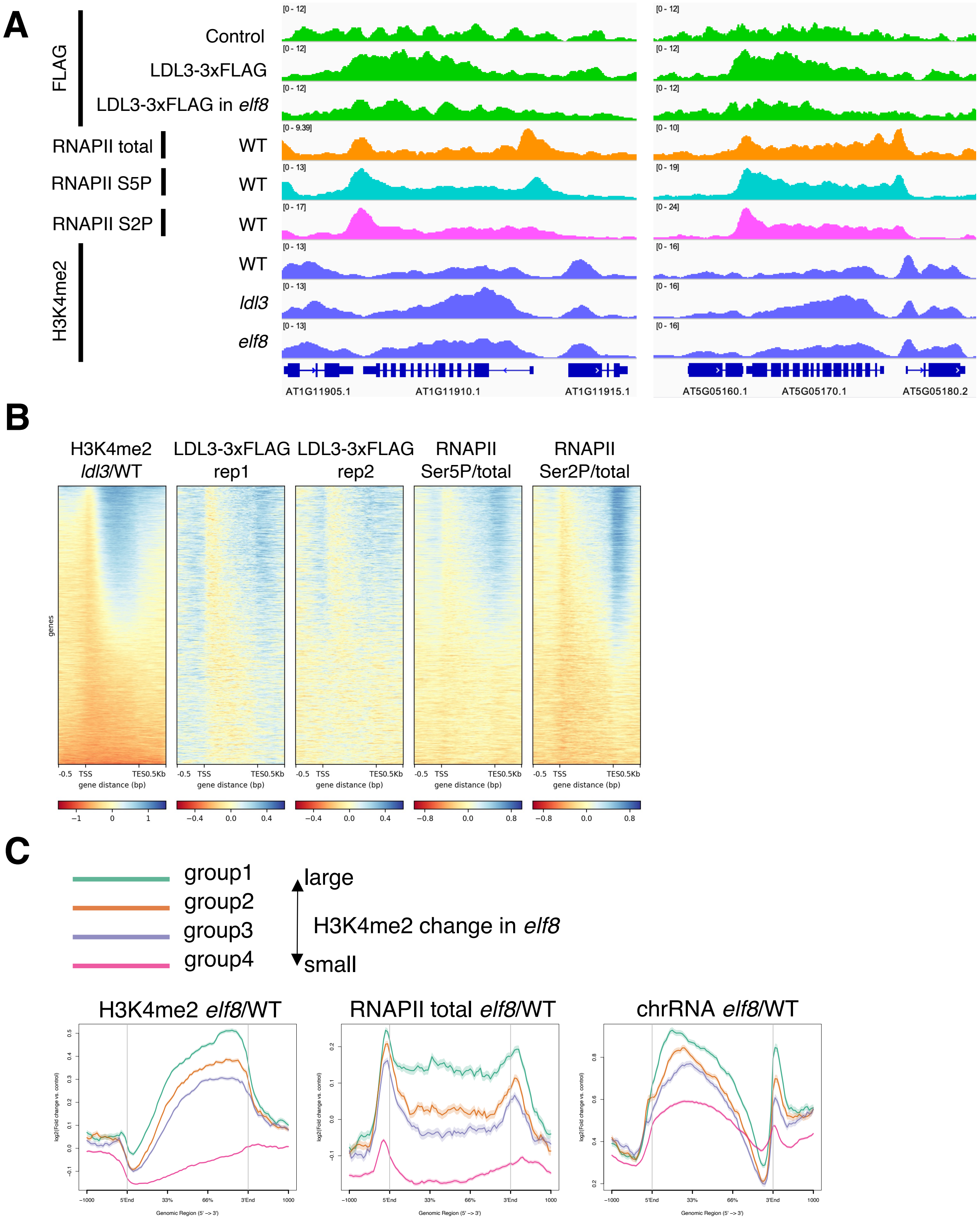

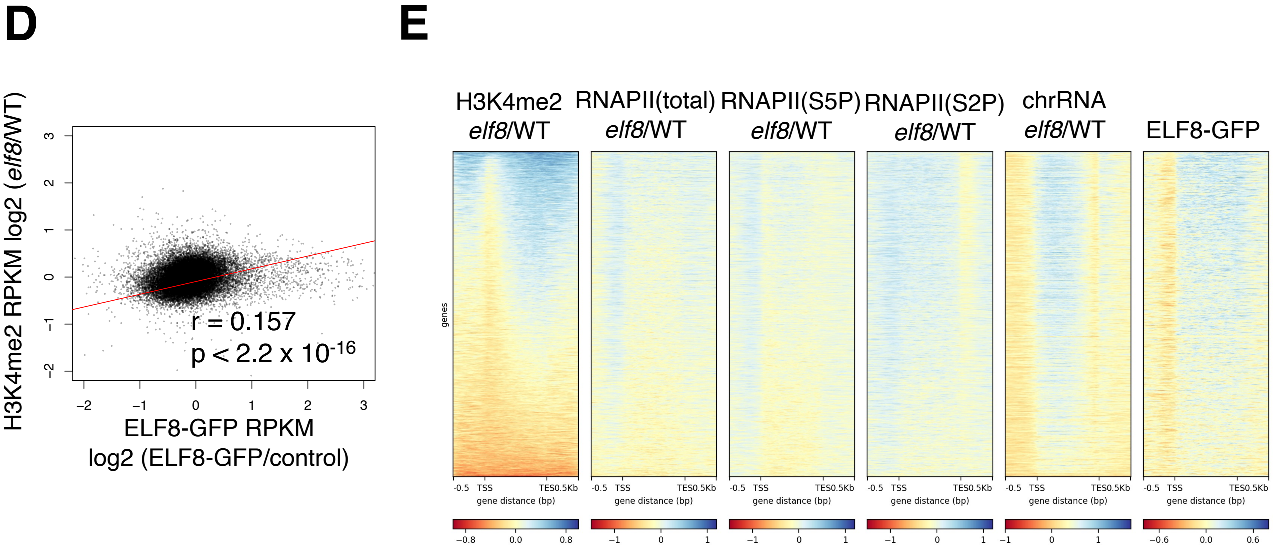

Appendix Figure S13. The accumulation of H3K4me2 in the *elf8* mutant is mostly mediated by *LDL3*.

(A) Screenshots of LDL3-3xFLAG and H3K4me2 ChIP-seq signals in putative LDL3 target genes (B) Heatmaps depicting changes in H3K4me2 levels in *ldl3*, the LDL3 localization, and RNAPII phosphorylation levels. LDL3 localization is corrected with a nontransgenic control. The protein-coding genes were sorted based on increases in gene-body H3K4me2 levels in *ldl3*. (C) Averaged profiles of H3K4me2 changes, chrRNA changes, and RNAPII changes between WT and *elf8*. All protein-coding genes were grouped based on levels of change in H3K4me2. Group1: 5 < *elf8*-WT (RPKM) (green, 2,809 genes); Group2: 3 < *elf8*-WT (RPKM) < 5 (orange, 2,919 genes); Group3: 1 < *elf8*-WT (RPKM) < 3 (blue, 3234 genes); and Group4: *elf8*-WT (RPKM) < 1 (pink, 17,721 genes). (D) Relationships between the ELF8 localization corrected with a non-transgenic control and changes in H3K4me2 levels (RPKM) in *elf8*. (E) Heatmaps depicting changes in H3K4me2 levels, RNAPII levels (total or phosphorylated), and chrRNA levels between WT and *elf8*, and the ELF8 localization. ELF8 localization is corrected with a nontransgenic control. The protein-coding genes were sorted based on increases in gene-body H3K4me2 levels in *elf8*.

Appendix Figure S14

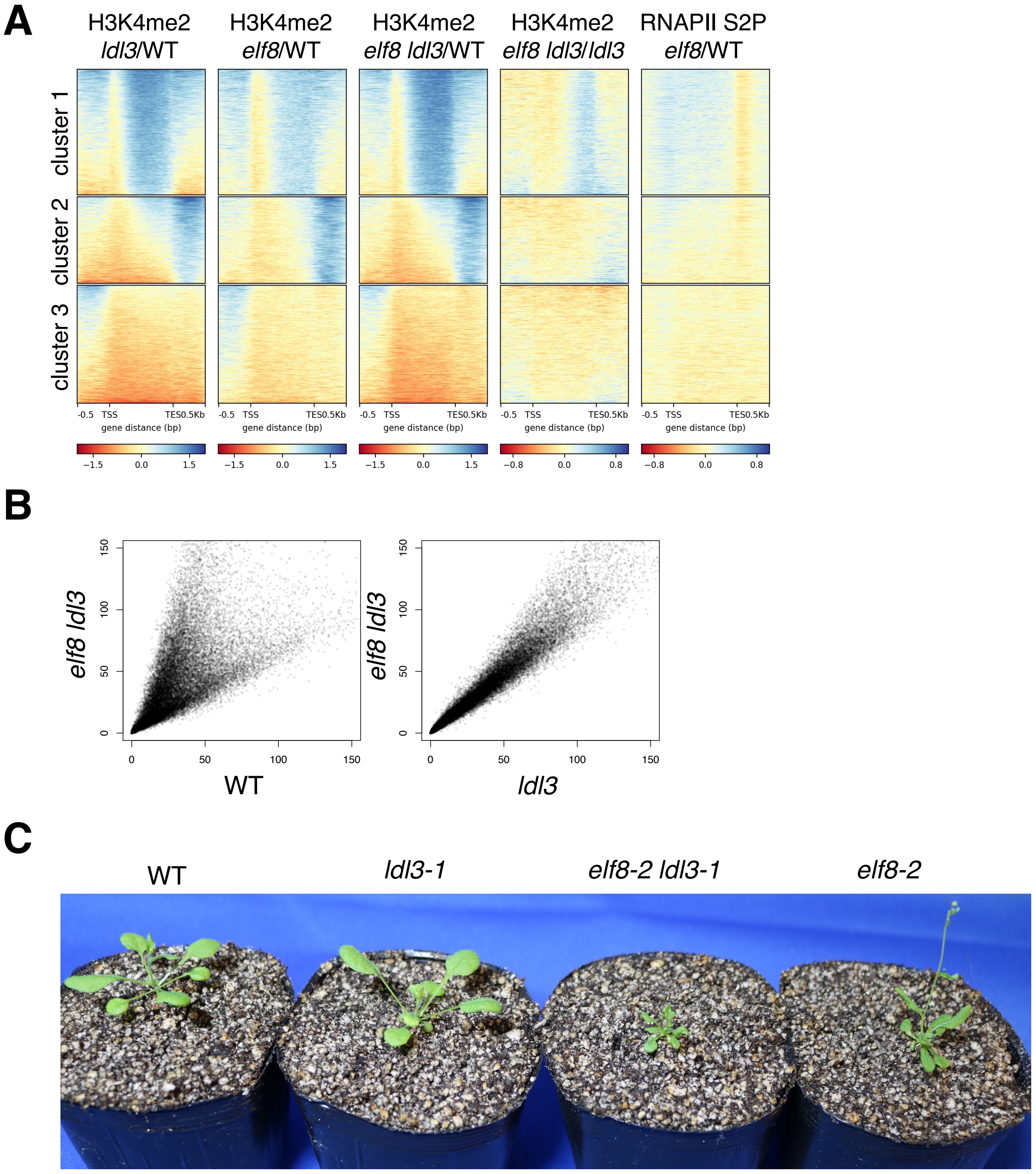

Appendix Figure S14. Analysis of the *elf8 ldl3* double mutants.

(A) Heatmaps depicting changes in H3K4me2 levels in each genotype and changes in RNAPII S2P levels in the *elf8* mutants over three clusters. cluster 1: 10329 genes, cluster 2: 7103 genes, cluster 3: 9610 genes. (B) H3K4me2 levels in *elf8 ldl3* double mutant compared with WT (left) or *ldl3* (right). Each dot represents the RPM within each protein-coding gene. (C) The *ldl3* mutant has wild type appearance, but the *ldl3* mutation enhances the *elf8* mutant phenotype.

Appendix Figure S15

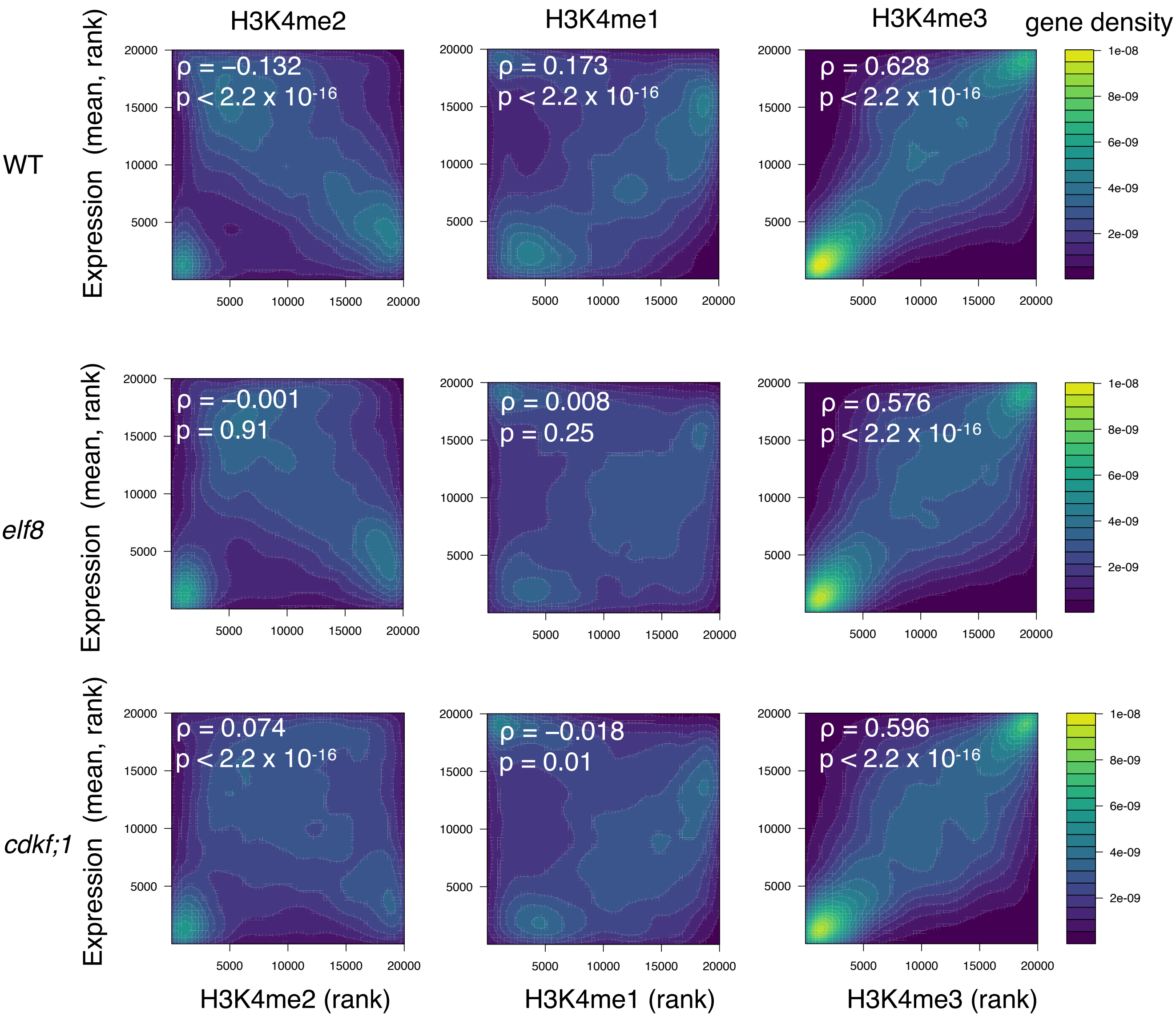

Appendix Figure S15. Correlations between H3K4me and transcription of protein-coding genes in WT, *elf8*, and *cdkf;1*.

Protein-coding genes are ranked by H3K4me1, me2 or me3 (x-axis, RPKM) and expression levels (y-axis, chrRNA-seq RPKM), respectively. The data of WT is the same as Fig. 6A. The densities of genes are visualized as heat maps. Genes for which no transcription was detected were excluded. ρ: Spearman's correlation coefficient.

Appendix Figure S16

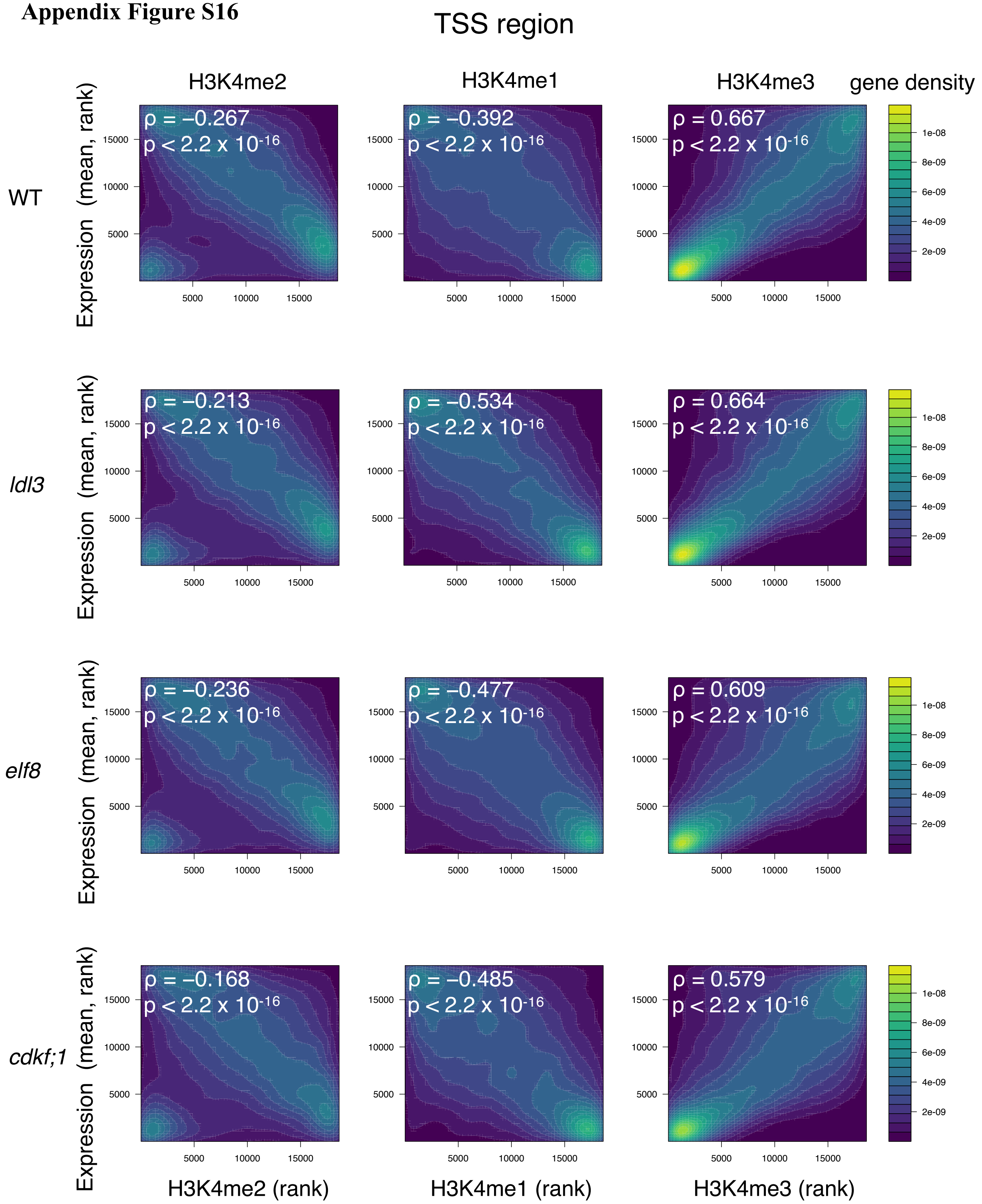

Appendix Figure S16. Correlations between H3K4me in TSS region and transcription of protein-coding genes in WT, *ldl3*, *elf8*, and *cdkf;1*.

Protein-coding genes are ranked by H3K4me1, me2 or me3 (x-axis, RPKM) in the TSS region (TSS to 500 bp downstream of TSS) and expression levels (y-axis, chrRNA-seq RPKM), respectively. The densities of genes are visualized as heat maps. ρ: Spearman's correlation coefficient. Genes shorter than 1.0 kb are excluded.

Appendix Figure S17

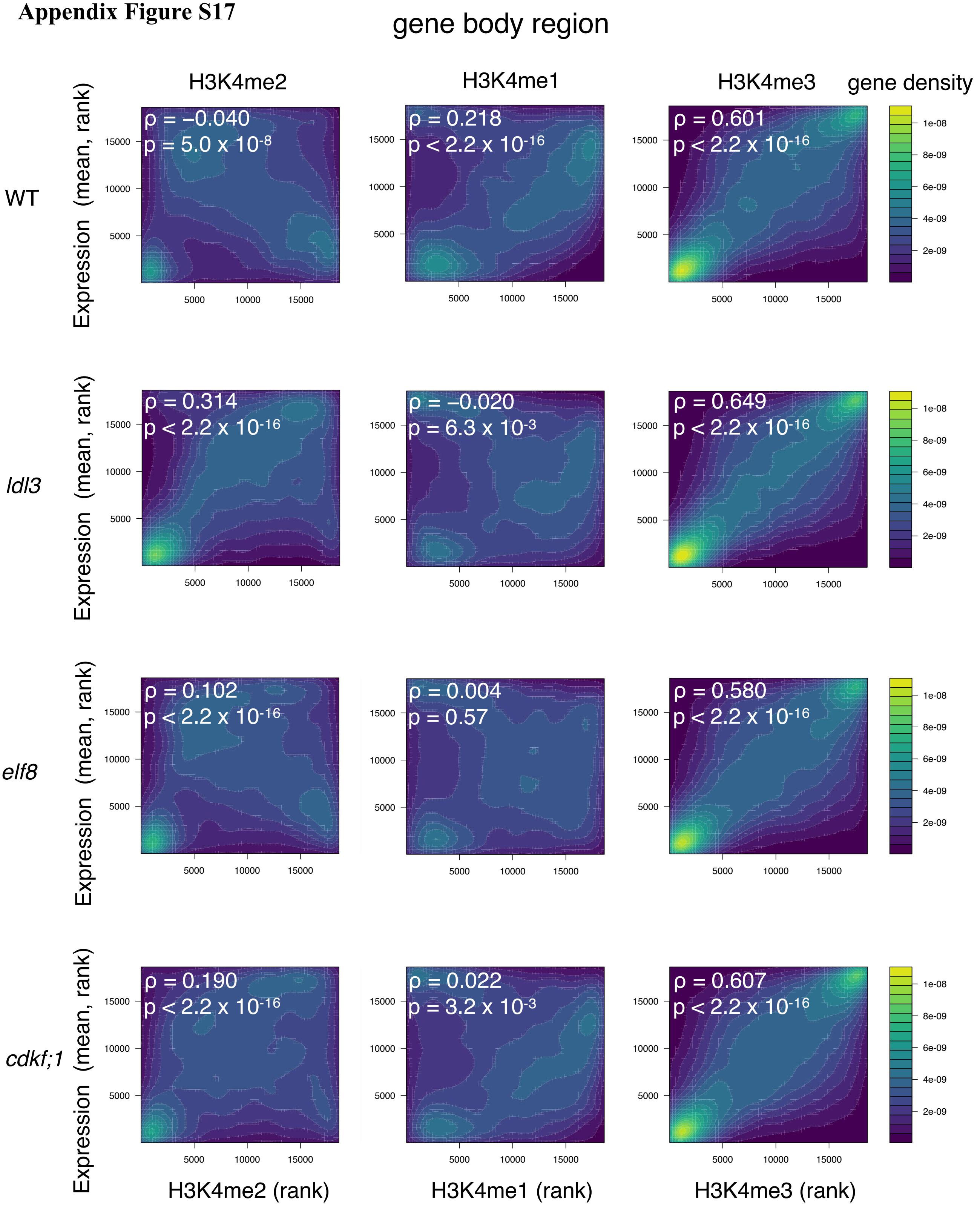

Appendix Figure S17. Correlations between H3K4me in gene body region and transcription of protein-coding genes in WT, *ldl3*, *elf8*, and *cdkf;1*.

Protein-coding genes are ranked by H3K4me1, me2 or me3 (x-axis, RPKM) in the gene body region (500 bp downstream of TSS to TTS) and expression levels (y-axis, chrRNA-seq RPKM), respectively. The densities of genes are visualized as heat maps. ρ: Spearman's correlation coefficient. Genes shorter than 1.0 kb are excluded.

Appendix Figure S18

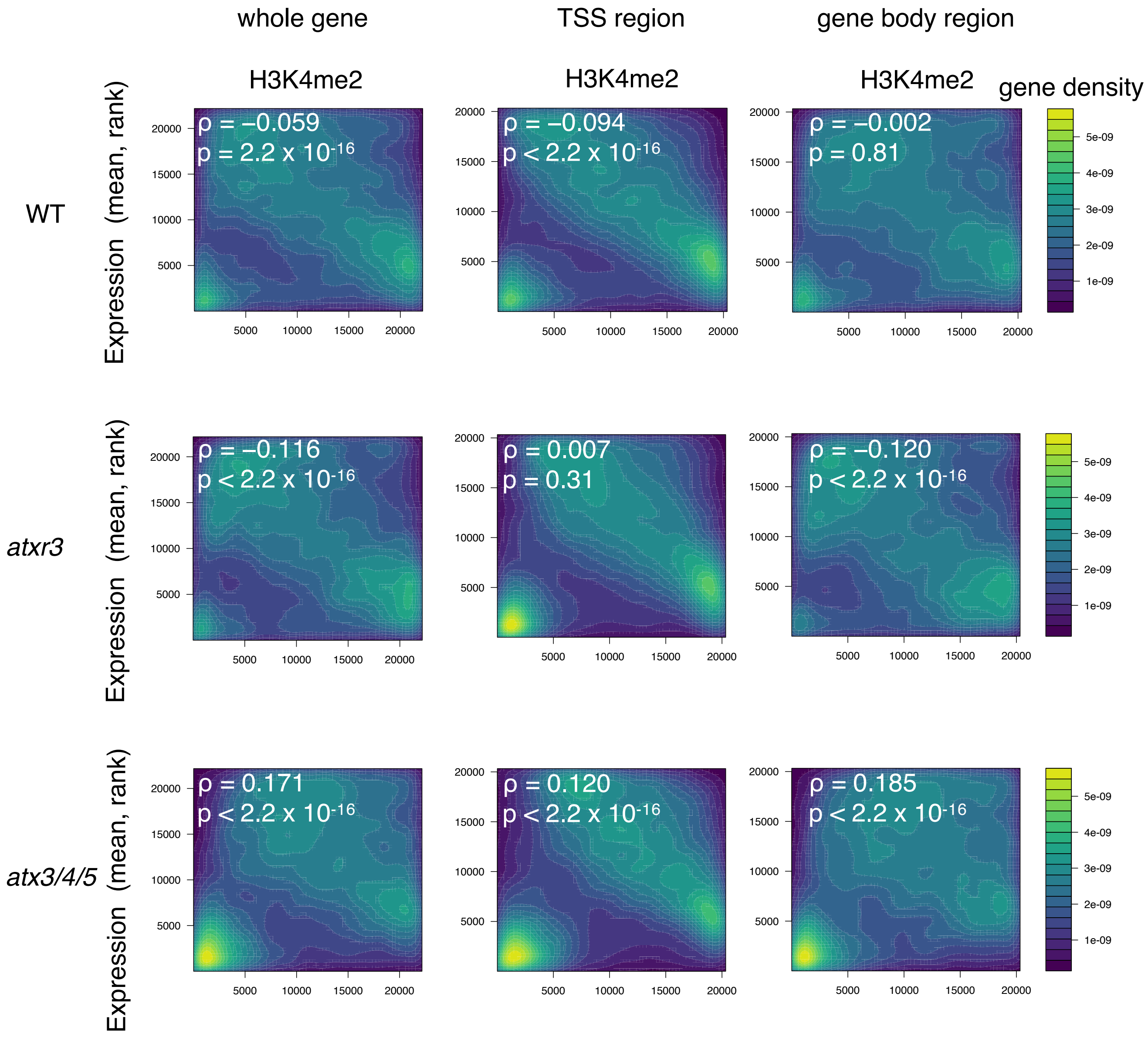

Appendix Figure S18. Correlations between H3K4me2 in each region and transcription of protein-coding genes in WT, *atxr3*, and *atx3/4/5*.

Protein-coding genes are ranked by H3K4me2 (x-axis, RPKM) and expression levels (y-axis, chrRNA-seq RPKM), respectively. H3K4me2 levels were calculated in the whole gene (left), the TSS region (center) and the gene body region (right). The densities of genes are visualized as heat maps. ρ: Spearman's correlation coefficient.

Appendix Figure S19

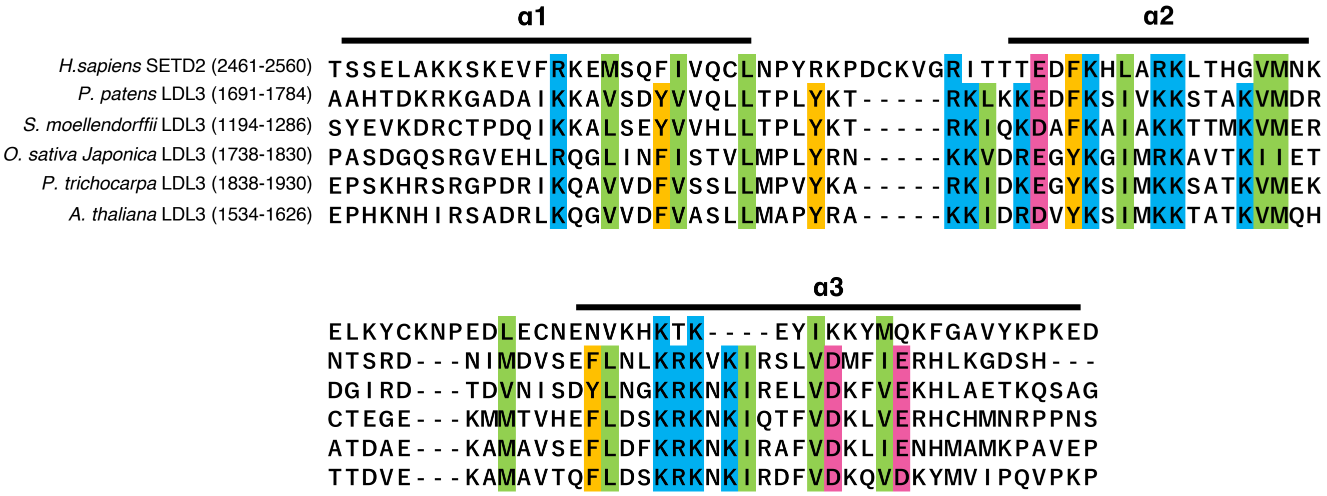

Appendix Figure S19. Amino acid alignment of the SRI domain in LDL3 and its homologs.

Regular secondary structure of the SRI domains in *H. sapiens* SETD2 is reported above the alignment. The SRI domain forms a closed three-helix bundle (α1, α2, α3). In the alignment, positions conserved or highly similar among the entire SRI family are colored in green for hydrophobic amino acids (VILM), orange for aromatic amino acids (FYW), blue for basic (KRH) amino acids, and pink for acidic (DE) amino acids. Host species, protein names and domain limits (in parentheses) are indicated at the start of the sequences. NCBI accession numbers are as follows : *Homo sapiens* SETD2 : Q9BYW2, *Physcomitrella patens* LDL3 : XP_024400419, *Selaginella moellendorffii* LDL3 : XP_024522564, *Oryza sativa japonica* LDL3 : XP_015614418, *Popilus trichocarpa* LDL3 : XP_002300727, *Arabidopsis thaliana* LDL3 : OAO99627
