# Supplementary material for: Cotranscriptional demethylation induces global loss of H3K4me2 from active genes in Arabidopsis
Source: EMBO J. 2023 Oct 18;42(23):e113798. doi: 10.15252/embj.2023113798 (PMC10690457; doi:10.15252/embj.2023113798)
Supplement: Supplementary file 2 — Source Data for Figure 2 [file EMBJ-42-e113798-s002.zip › Figure_2/2E/2E_S7D.pdf]

H3K4me2
(for K4me2 rep_1)

H3K4me3
(for K4me3 rep_1)

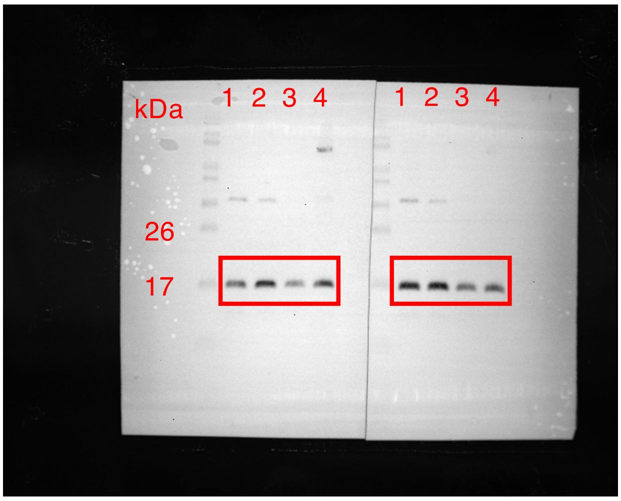

H3
(for K4me2 rep_1)

H3
(for K4me3 rep_1)

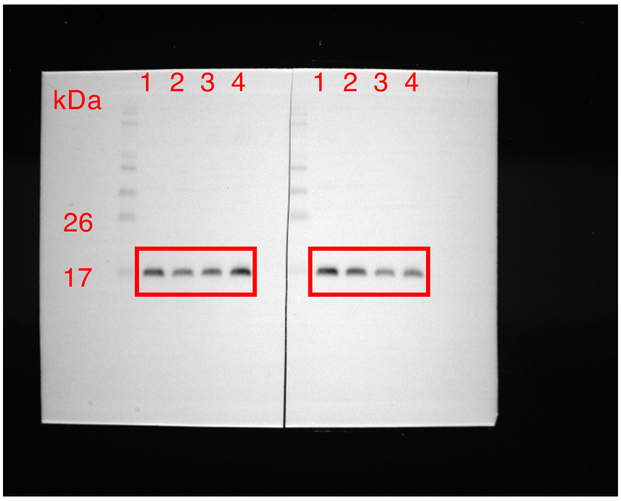

H3K4me2
(for K4me2 rep_2)

H3K4me3
(for K4me3 rep_2)

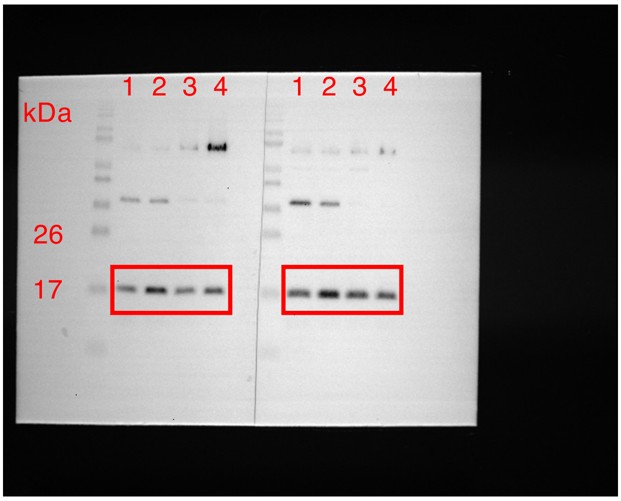

H3
(for K4me2 rep_2)

H3
(for K4me3 rep_2)

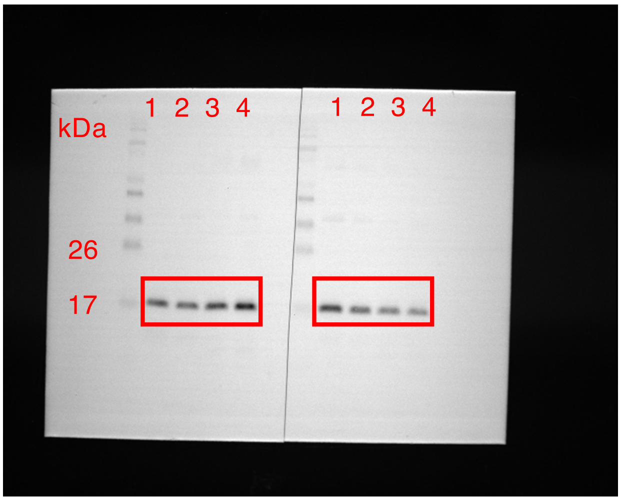

H3K4me2
(for K4me2 rep_3)

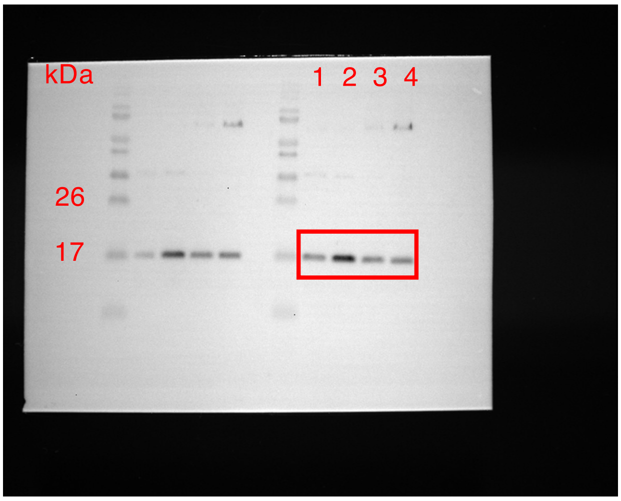

H3
(for K4me2 rep_3)

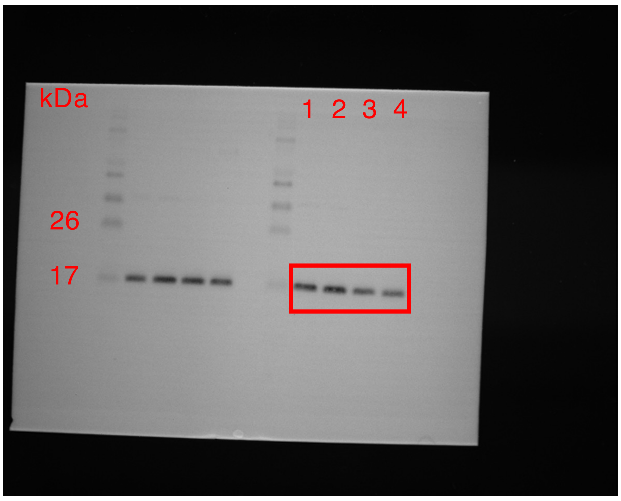

H3K4me1
(for K4me1 rep_1)

H3K4me1
(for K4me1 rep_2)

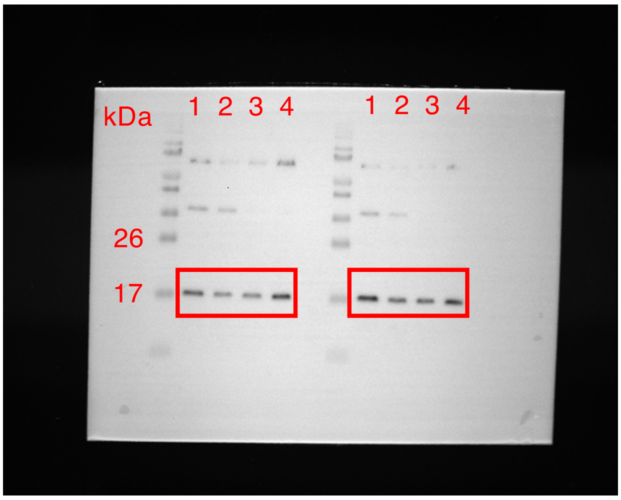

H3
(for K4me1 rep_1)

H3
(for K4me1 rep_2)

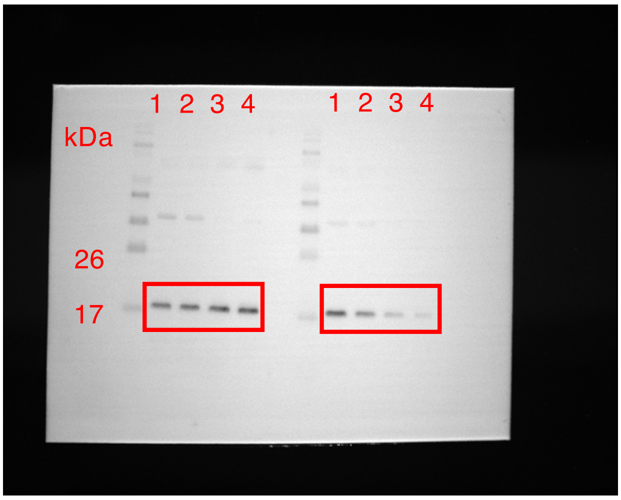

H3K4me3
(for K4me3 rep_3)

H3K4me1
(for K4me1 rep_3)

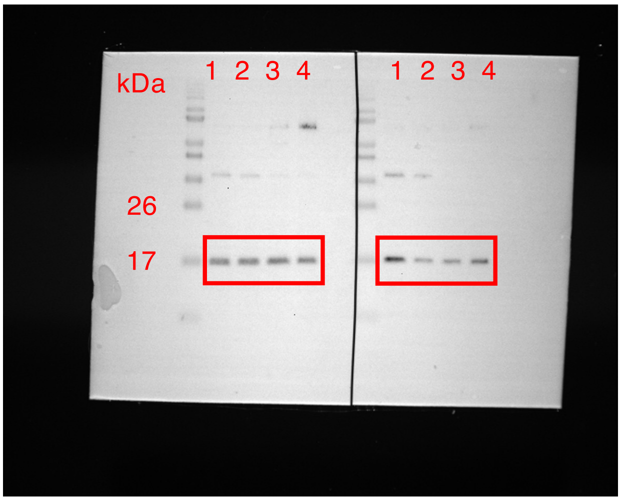

H3
(for K4me3 rep_3)

H3
(for K4me1 rep_3)

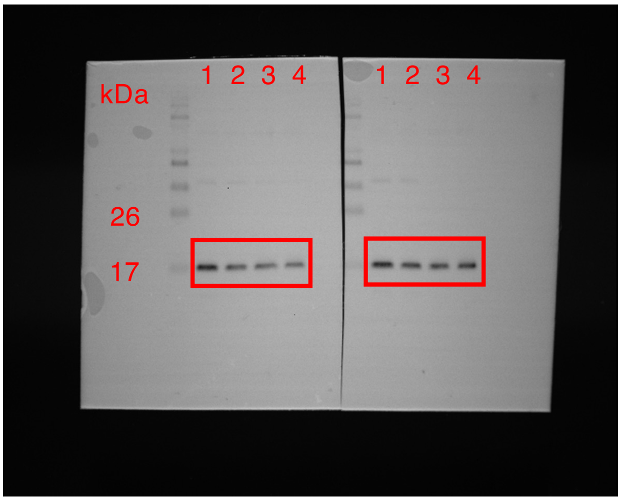

<lane>

1. WT, 2. *ldl3*, 3. *elf8*, 4. *cdkf;1*
