# Supplementary material for: Cotranscriptional demethylation induces global loss of H3K4me2 from active genes in Arabidopsis
Source: EMBO J. 2023 Oct 18;42(23):e113798. doi: 10.15252/embj.2023113798 (PMC10690457; doi:10.15252/embj.2023113798)
Supplement: Supplementary file 3 — Source Data for Figure 3 [file EMBJ-42-e113798-s004.zip › Figure_3/3B/3B.pdf]

RNAPII(total)

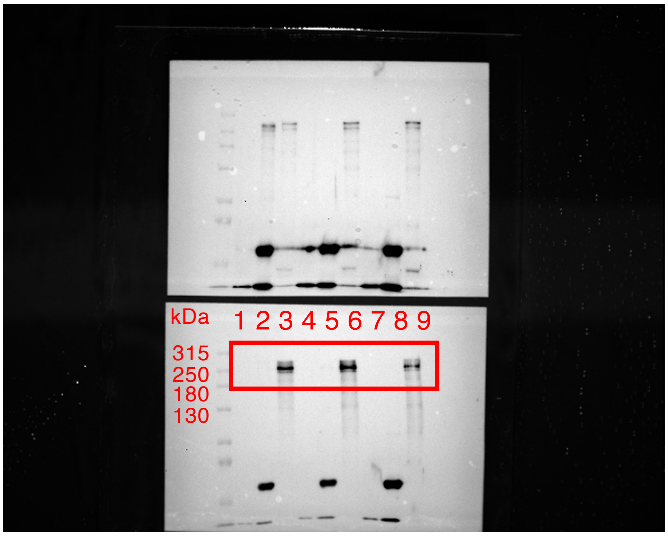

RNAPII (Ser5P)

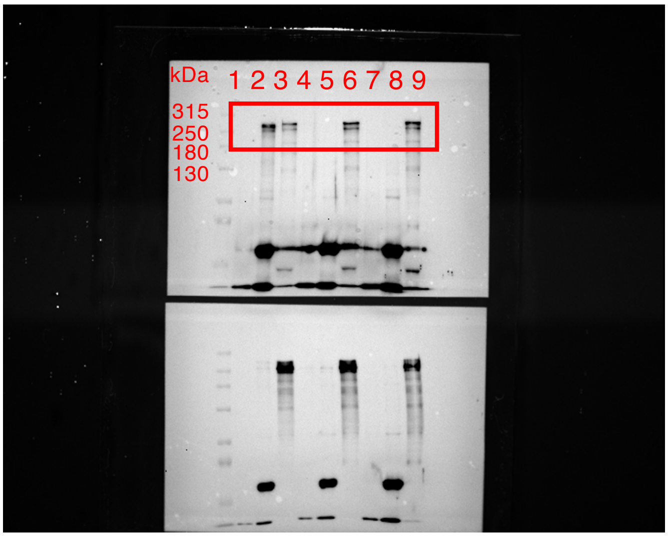

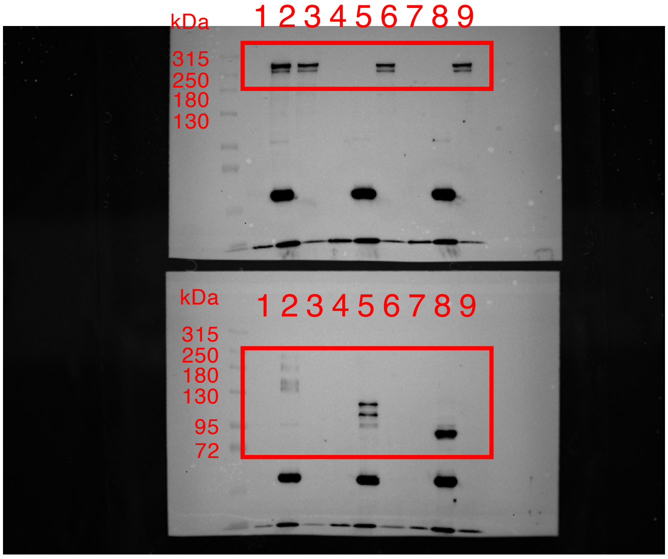

RNAPII (Ser2P)

FLAG

<lane>

1. LDL3 IP-, 2. LDL3 IP+, 3.LDL3 input

4. FLD IP-, 5. FLD IP+, 6.FLD input

7. LDL2 IP-, 8. LDL2 IP+, 9.LDL2 input
