# Supplementary figures and images for: Cotranscriptional demethylation induces global loss of H3K4me2 from active genes in Arabidopsis
Source: EMBO J. 2023 Oct 18;42(23):e113798. doi: 10.15252/embj.2023113798 (PMC10690457; doi:10.15252/embj.2023113798)

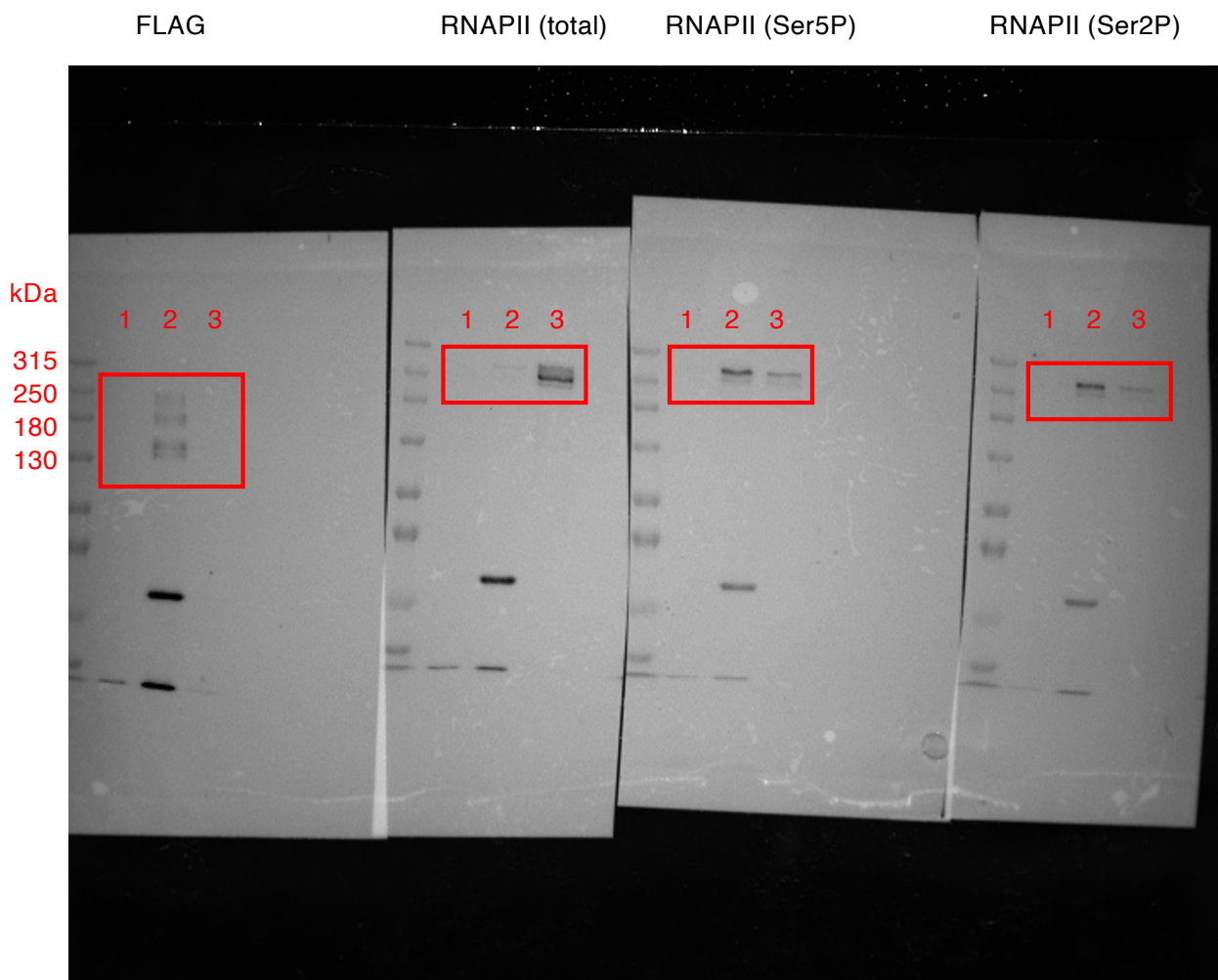

<lane>

1. IP-, 2. IP+, 3. input

Supplement: Supplementary file 3 — Source Data for Figure 3 [file EMBJ-42-e113798-s004.zip › Figure_3/3A/3A.pdf]

RNAPII(total)

RNAPII (Ser5P)

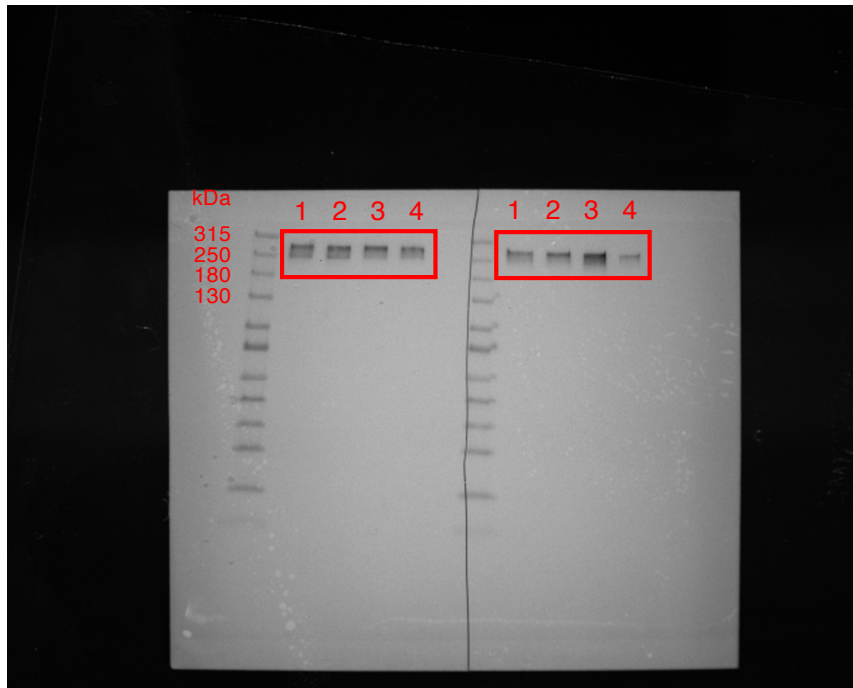

RNAPII (Ser2P)

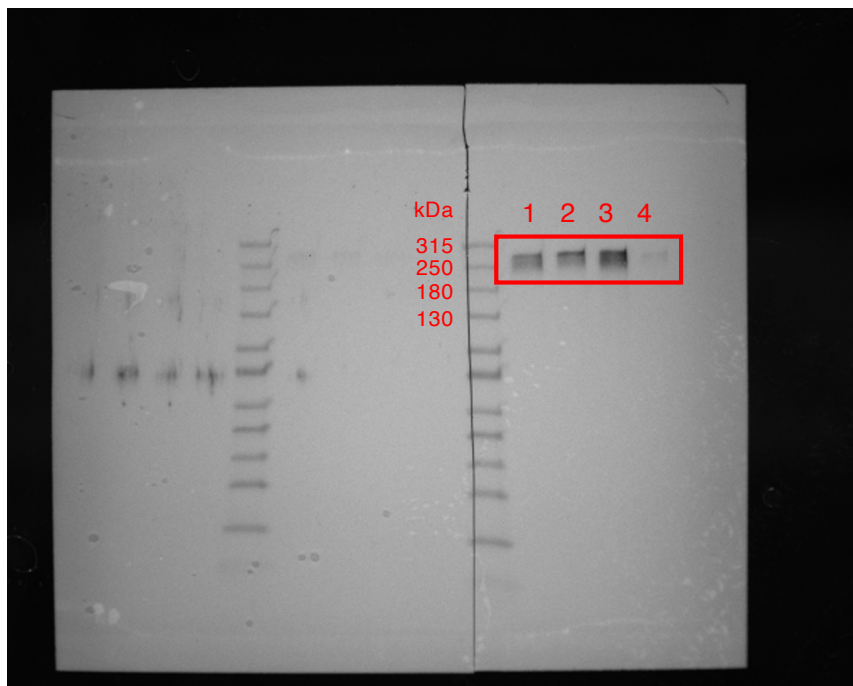

<lane>

1. WT, 2. *Idl3*, 3. *elf8*, 4. *cdkf;1*

Supplement: Supplementary file 4 — Source Data for Figure 4 [file EMBJ-42-e113798-s006.zip › Figure_4/4A/4A.pdf]
