# Supplementary material for: Cotranscriptional demethylation induces global loss of H3K4me2 from active genes in Arabidopsis
Source: EMBO J. 2023 Oct 18;42(23):e113798. doi: 10.15252/embj.2023113798 (PMC10690457; doi:10.15252/embj.2023113798)
Supplement: Supplementary file 7 [file EMBJ-42-e113798-s007.pdf]

THE EMBO JOURNAL

Cotranscriptional demethylation induces global loss of H3K4me2 from active genes in Arabidopsis

Shusei Mori, Satoyo Oya, Mayumi Takahashi, Kazuya Takashima, Soichi Inagaki, and Tetsuji Kakutani
DOI: 10.15252/embj.2023113798

Corresponding author(s): Shusei Mori (smori@bs.s.u-tokyo.ac.jp), Soichi Inagaki (soinagak@bs.s.u-tokyo.ac.jp), Tetsuji Kakutani (tkak@bs.s.u-tokyo.ac.jp)

Review Timeline:

Submission Date:	17th Feb 23
Editorial Decision:	30th Mar 23
Revision Received:	18th Jul 23
Editorial Decision:	23rd Aug 23
Revision Received:	7th Sep 23
Editorial Decision:	19th Sep 23
Revision Received:	20th Sep 23
Accepted:	25th Sep 23

Editor: William Teale

Transaction Report:

(Note: With the exception of the correction of typographical or spelling errors that could be a source of ambiguity, letters and reports are not edited. Depending on transfer agreements, referee reports obtained elsewhere may or may not be included in this compilation. Referee reports are anonymous unless the Referee chooses to sign their reports.)

1st Editorial Decision **30th Mar 2023**

Dear Dr. Kakutani,

Thank you again for the submission of your manuscript entitled "Cotranscriptional demethylation induces global loss of H3K4me2 from active genes in Arabidopsis" (EMBOJ-2023-113798) and for your patience during the review process. We have now received reports from all three referees, which I copy below.

As you can see from their comments, while referees #1 and #2 had significant concerns over some aspects of your experimental design, and the interpretation of your data, all reviewers saw a set of experiments with the potential to be published in EMBO Journal. That said, concerns over your definition of genetic memory, comparisons among data sets, and assumptions you make about the relationship between LDL3 inactivation and H3K4 demethylation will require your attention before your manuscript can be published in The EMBO Journal.

Based on the overall interest expressed in the reports, therefore, I would like to invite you to address the comments of all referees in a revised version of the manuscript. I should add that it is The EMBO Journal policy to allow only a single major round of revision and that it is therefore important to resolve the main concerns at this stage. I believe the concerns of the referees are reasonable and addressable, but please contact me if you have any questions, need further input on the referee comments or if you anticipate any problems in addressing any of their points. I am always available, should you wish to discuss any of the referee reports with me over Zoom. Please, follow the instructions below when preparing your manuscript for resubmission.

I would also like to point out that as a matter of policy, competing manuscripts published during this period will not be taken into consideration in our assessment of the novelty presented by your study ("scooping" protection). We have extended this 'scooping protection policy' beyond the usual 3 month revision timeline to cover the period required for a full revision to address the essential experimental issues. Please contact me if you see a paper with related content published elsewhere to discuss the appropriate course of action.

When preparing your letter of response to the referees' comments, please bear in mind that this will form part of the Review Process File, and will therefore be available online to the community. For more details on our Transparent Editorial Process, please visit our website: https://www.embopress.org/page/journal/14602075/authorguide#transparentprocess

Again, please contact me at any time during revision if you need any help or have further questions.

Thank you very much again for the opportunity to consider your work for publication. I look forward to your revision.

Best regards,

William Teale

William Teale, Ph.D.
Editor
The EMBO Journal

When submitting your revised manuscript, please carefully review the instructions below and include the following items:

1) a .docx formatted version of the manuscript text (including legends for main figures, EV figures and tables). Please make sure that the changes are highlighted to be clearly visible.

2) individual production quality figure files as .eps, .tif, .jpg (one file per figure).

3) a .docx formatted letter INCLUDING the reviewers' reports and your detailed point-by-point response to their comments. As part of the EMBO Press transparent editorial process, the point-by-point response is part of the Review Process File (RPF), which will be published alongside your paper.

4) a complete author checklist, which you can download from our author guidelines (https://wol-prod-cdn.literatumonline.com/pb-assets/embo-site/Author Checklist%20-%20EMBO%20J-1561436015657.xlsx). Please insert information in the checklist that is also reflected in the manuscript. The completed author checklist will also be part of the RPF.

5) Please note that all corresponding authors are required to supply an ORCID ID for their name upon submission of a revised manuscript.

6) We require a 'Data Availability' section after the Materials and Methods. Before submitting your revision, primary datasets produced in this study need to be deposited in an appropriate public database, and the accession numbers and database listed under 'Data Availability'. Please remember to provide a reviewer password if the datasets are not yet public (see https://www.embopress.org/page/journal/14602075/authorguide#datadeposition). If no data deposition in external databases is needed for this paper, please then state in this section: This study includes no data deposited in external repositories. Note that the Data Availability Section is restricted to new primary data that are part of this study.

Note - All links should resolve to a page where the data can be accessed.

7) When assembling figures, please refer to our figure preparation guideline in order to ensure proper formatting and readability in print as well as on screen:
http://bit.ly/EMBOPressFigurePreparationGuideline

Please remember: Digital image enhancement is acceptable practice, as long as it accurately represents the original data and conforms to community standards. If a figure has been subjected to significant electronic manipulation, this must be noted in the figure legend or in the 'Materials and Methods' section. The editors reserve the right to request original versions of figures and the original images that were used to assemble the figure.

8) For data quantification: please specify the name of the statistical test used to generate error bars and P values, the number (n) of independent experiments (specify technical or biological replicates) underlying each data point and the test used to calculate p-values in each figure legend. The figure legends should contain a basic description of n, P and the test applied. Graphs must include a description of the bars and the error bars (s.d., s.e.m.).

9) We would also encourage you to include the source data for figure panels that show essential data. Numerical data can be provided as individual .xls or .csv files (including a tab describing the data). For 'blots' or microscopy, uncropped images should be submitted (using a zip archive or a single pdf per main figure if multiple images need to be supplied for one panel). Additional information on source data and instruction on how to label the files are available at .

10) We replaced Supplementary Information with Expanded View (EV) Figures and Tables that are collapsible/expandable online (see examples in https://www.embopress.org/doi/10.15252/embj.201695874). A maximum of 5 EV Figures can be typeset. EV Figures should be cited as 'Figure EV1, Figure EV2" etc. in the text and their respective legends should be included in the main text after the legends of regular figures.

- For the figures that you do NOT wish to display as Expanded View figures, they should be bundled together with their legends in a single PDF file called *Appendix*, which should start with a short Table of Content. Appendix figures should be referred to in the main text as: "Appendix Figure S1, Appendix Figure S2" etc. See detailed instructions regarding expanded view here: .

- Additional Tables/Datasets should be labelled and referred to as Table EV1, Dataset EV1, etc. Legends have to be provided in a separate tab in case of .xls files. Alternatively, the legend can be supplied as a separate text file (README) and zipped together with the Table/Dataset file.

11) At EMBO Press we ask authors to provide source data for the main manuscript figures. Our source data coordinator will contact you to discuss which figure panels we would need source data for and will also provide you with helpful tips on how to upload and organize the files.

12) Our journal encourages inclusion of *data citations in the reference list* to directly cite datasets that were re-used and obtained from public databases. Data citations in the article text are distinct from normal bibliographical citations and should directly link to the database records from which the data can be accessed. In the main text, data citations are formatted as follows: "Data ref: Smith et al, 2001" or "Data ref: NCBI Sequence Read Archive PRJNA342805, 2017". In the Reference list, data citations must be labeled with "[DATASET]". A data reference must provide the database name, accession number/identifiers and a resolvable link to the landing page from which the data can be accessed at the end of the reference. Further instructions are available at .

Further instructions for preparing your revised manuscript:

Please make sure you upload a letter of response to the referees' comments together with the revised manuscript.

Please also check that the title and abstract of the manuscript are brief, yet explicit, even to non-specialists.

When assembling figures, please refer to our figure preparation guideline in order to ensure proper formatting and readability in print as well as on screen:
https://bit.ly/EMBOPressFigurePreparationGuideline
See also guidelines for figure legends: https://www.embopress.org/page/journal/14602075/authorguide#figureformat

At EMBO Press we ask authors to provide source data for the main manuscript figures. Our source data coordinator will contact you to discuss which figure panels we would need source data for and will also provide you with helpful tips on how to upload and organize the files.

IMPORTANT: When you send the revision we will require
- a point-by-point response to the referees' comments, with a detailed description of the changes made (as a word file).
- a word file of the manuscript text.
- individual production quality figure files (one file per figure)
- a complete author checklist, which you can download from our author guidelines (https://www.embopress.org/page/journal/14602075/authorguide).
- Expanded View files (replacing Supplementary Information)
Please see out instructions to authors
https://www.embopress.org/page/journal/14602075/authorguide#expandedview

Please remember: Digital image enhancement is acceptable practice, as long as it accurately represents the original data and conforms to community standards. If a figure has been subjected to significant electronic manipulation, this must be noted in the figure legend or in the 'Materials and Methods' section. The editors reserve the right to request original versions of figures and the original images that were used to assemble the figure.

Further information is available in our Guide For Authors: https://www.embopress.org/page/journal/14602075/authorguide

We realize that it is difficult to revise to a specific deadline. In the interest of protecting the conceptual advance provided by the work, we recommend a revision within 3 months (28th Jun 2023). Please discuss the revision progress ahead of this time with the editor if you require more time to complete the revisions. Use the link below to submit your revision:

https://emboj.msubmit.net/cgi-bin/main.plex

Referee #1:

Previous papers showed that plants use LDL3 to co-transcriptionally remove H3K4me2 within transcribed regions, reversing the typically positive correlation between transcription and H3K4 methylation states. This paper attempts to correlate this effect with other features of transcription, concluding that both CTD Serine 2 phosphorylation and the PAF1 Complex contribute to LD3 function. The paper presents good evidence for LDL3 binding to the RNA pol II CTD during elongation. Overall, the story seems pretty consistent. However, some things don't quite make sense, perhaps due to the way the results are analyzed.

Major concerns:

1. Many of the mutations tested here are likely to have systemic, rather than gene-specific, effects. It is now well-recognized that any genomic technique (ChIP-seq, nascent RNA seq, etc) wil miss these effects without some type of spiked-in normalization method, typically a corresponding sample from some other organism that can go through the entire library preparation but be distinguished at the computational level (see Chen et al., Mol Cell Biol. 2016 36(5): 662-667; Loven et al. Cell 151:476-482. doi: 10.1016/j.cell.2012.10.012). Unfortunately, this paper only normalizes by "reads per million", which assumes that any changes are localized and occur against a background where most gene profiles do not change. That seems unlikely here. The proper thing would be to re-do the experiments here with proper spike-ins, but obviously that would be a huge amount of work. There are a few methods around for using other genomic features as internal normalizations. I suggest the authors talk to some more experienced genomics labs to see if they can find a method to substitute.

2. One very obvious place where this problem is occurring is shown in Fig S2. In other organisms, knocking out H2Bub completely blocks H3K4 methylation. The structure of Set1/COMPASS shows exactly how the ubiquitin allosterically activates the methyltransferase. Yet the ChIP here shows almost no effect. I would like to see the western for H3K4me2, since I find this very hard to believe. Either Arabidopsis K4 methyltransferase is completely different from both yeast and mammals in not sensing H2Bub, or improper normalization of the ChIP-seq is giving a misleading result. This same effect could be affecting the other genomic experiments as well.

3. Similarly, it's odd that Paf1C mutants would produce more H3K4me2, since overall transcription is probably down. What happens to H3K4me3? It may be more likely that me2 increases because there is less me3, not because of an effect on the demethylase. Or the normalization may be hiding effects.

4. It's interesting that some of the mutants seem to shift the distribution of K4me2 along genes. However, the assignment of

LDL3 "target" genes and other mutation effects is based on total H3K4me2 read counts (as in Fig 1A). Isn't it possible that may target genes could be missed if the overall amount of H3K4me2 remains the same but the peaks shift considerably? In other words, could there be genes where changes are qualitative, but not quantitative?

5. Since the authors have a FLAG-tagged LDL3 construct and show they can co-IP RNA pol II, I was surprised they did not directly look at LDL3 recruitment in the various mutants using ChIP-seq. For example, by their model, mutating the CTD kinase should lead to loss of LDL3 signal. This would be an important test of the model.

6. I don't understand the assertion that H3K4me2 acts as a "memory"? In what sense? "Transcriptional memory" is typically defined in terms of gene induction, which in some cases produces a different response between the first and second inductions. But nothing like that is tested here. Please define what you mean by "memory" and how you would assay it. Otherwise, if this is just a vague idea, the term "memory" should be dropped because it will be confused with the existing literature.

Minor issues:

1. Line 163. Several papers in yeast have shown that H3K36 methylation is dependent on Paf1C function (for example, Krogan et al. MCB 2003).

2. Fig6. Given that K4 methylation is transcription-directed, the correlation coefficients are surprisingly low (all below 0.2) for K4me2 and me1 (especially compared to K36me3). Any idea why?

3. The introduction mentions that it seems paradoxical that H3K4me2 could be repressive for transcription. In fact, there are several papers showing K4me2 in yeast recruits the Set3 HDAC, which can suppress elongation and internal cryptic initiation sites.

Referee #2:

This study provides interesting data regarding H3K4 methylation and transcription, including genome-wide distribution of H3K4me2, Pol II and chrRNA in an important set of mutants. It also demonstrates the direct interaction of LDL3 with phosphorylated Pol II. However, there are some concerns with interpretation of the data and emphasis of the conclusions that might confuse readers.

Major concerns:

The authors emphasise the fact that H3K4me2 correlates negatively with transcription. Any correlation of H3K4me2 enrichment with repression could just be a consequence of the transition to H3K4me3 being less likely in a repressed state - i.e. less H3K4me3 means more H3K4me2. Conversely, in an activated state, more H3K4me3 means less H3K4me2. The is in fact observed in some of the cited data (Wang et al., 2022). Essentially, the three modification states - me1, me2, me3 must show some degree of mutual exclusivity since they are on the same residue. And this would have nothing to do with a repressive role for H3K4me2.

Including the H3K4me3 methyltransferase mutant would benefit this study (H3K4me2 accumulation and genetic analyses with cdkf;1 and elf8). ldl3, cdkf1, elf8, show increased H3K4me2 associated with reduced H3K4me1, reinforcing the mutual exclusivity of methylated states. This clarifies work in Ishihara et al., 2019, where changes in H3K4me2 between WT and ldl3 showed no clear effect on either K4me1 or K4me3.

Different modification states of H3K4 can behave differently in different parts of the gene. This is also seen in the genome wide data presented in Ishihara et al., 2019. It is important to expand the presentation/discussion of this when drawing conclusions about different methylation levels from genome wide correlations.

The authors claim throughout the manuscript that demethylation by LDL3 has an important role in chromatin state memory (lines 36-38, 98-10 and 327-330). What they mean about the memory aspect of H3K4me2 is quite speculative. Is it about priming/poising of transcription? Or is it about mitotic inheritance? Mitotic inheritance (across DNA replication) would require a feedback mechanism that is not addressed. For example, a recent paper tries to demonstrate mitotic inheritance of H3K4me2 in S. cerevisiae through read-write feedback (https://doi.org/10.7554/eLife.77646). This provides short term memory of transcriptional activation (they see it as an activating mark). The authors should cite this paper when they discuss the memory aspect.

It is quite interesting that they see increased H3K4me2 in elf8, where elongation is retarded, and consequently RNAP II hyper-accumulates. This would be quite consistent with a model where more time spent by RNAP II in a region increases the probability of adding more methylation at H3K4 - i.e. faster elongation only allows a short time window sufficient to add me1, while slower elongation allows more time to transition to me2. A model along these lines is proposed by Fong et al., 2017 proposes for H3K36me3 in mammals (https://doi.org/10.1016/j.molcel.2017.04.016). In the current manuscript, the authors suggest that the H3K4me2 accumulation in elf8 is mainly a consequence of disrupted LDL3 function. However, there is a large subset of "LDL3 targets", where the ldl3 elf8 double mutant shows higher H3K4me2 than the ldl3 single mutant (Cluster 5, Figure S10 (A)). The behaviour of these targets is consistent with an "increased dwell-time" model for elf8 as described above. It would be useful if the authors address this aspect more explicitly, perhaps by comparing changes in RNAP II occupancy in elf8 between.

It is not clear what the overall model is - i.e. how the authors reconcile the insights from their last paper (Oya et al., 2022) on H3K4 methyltransferases. They should address more clearly how they think H3K4me2 is added. Is the addition co-transcriptional too or mainly independent of transcription as in (https://doi.org/10.7554/eLife.77646)?

Minor comments:

Showing snapshots of the sequencing coverage or aligned reads in different loci would benefit the manuscript. This would provide particular examples showing the quality of the data and reveal interesting features that engage the reader such as promoter-proximal pausing, links to splicing, termination/readthrough etc...

The authors too often refer to changes in H3K4me2 as LDL3 function (without strong functional support). For example, in lines 28-30 "Here, we show that LDL3-mediated H3K4me2 demethylation depends on (...)" or lines 208-210: "These results suggest that although phosphorylation of RNAPII is necessary for LDL3 function, elf8 mutation affects LDL3 function through a different pathway." It is recommended to remove the reference to LDL3 in these phrases, changing to for example "changes in H3K4me2".

Fig. 5E - The genetic analysis of ldl3 and elf8 is important to support the claims in the manuscript. It would help to add the heatmap for H3K4me2 in ldl3 elf8 / WT as in most of the other figures (sorted by changes in H3K4me2 in ldl3 single).

Fig. 5E - elf8 is missing in the figure legend: "(E) H3K4me2 levels in ldl3 elf8 double mutant compared with WT (top) or (bottom).".

Editing is required in Results section (lines 257-272) and Fig 6A, particularly (lines 263-264): "These results suggest that LDL3 disrupts the positive correlation of transcription and H3K4me2 by removing H3K4me2 from transcribed genes."

The differences between the two left upper and lower plots in Fig 6A may reflect changes in H3K4me2 levels and therefore not very informative. Above all, the plots show no correlation (positive or negative) between H3K4me2 and chrRNA (Spearman's correlation ρ very close to zero). In this regard, changes must be made to lines 33-34 from the Abstract: "Importantly, the negative correlation between H3K4me2 and transcription is disrupted in the ldl3 mutant".

Referee #3:

The manuscript "Cotranscriptional demethylation induces global loss of H3K4me2 from active genes in Arabidopsis" by Shusei Mori and co-workers describes the interaction between the histone H3 lysine 4 dimethyl (H3K4me2) demethylase LDL3 with RNA-polymerase II, which seems dependent on the phosphorylation state of the latter. Indeed, mutations in factors that are required for proper RNAPII phosphorylation, involved in transcriptional progression, increase H3K4me2 level over LDL3 target genes. In contrast to H3K4me1 and K4me3, H3K4me2 is negatively associated with transcriptional activity. As a consequence, interfering with RNAPII phosphorylation leads to an increase in chromatin-associated RNA-accumulation over the affected loci, but not in free mRNA transcripts.

This is an interesting work that uncovers novel regulatory functions of chromatin-mediated transcriptional control that is potentially of high general interest to a large scientific community. The experiments seem sound and the manuscript is well written. I have to admit that there's a certain complexity and some puzzling results that make this a demanding lecture at times. Several observations fall into place, but others are somewhat contradictory. For instance, contrary to the expectation, the elf8 mutant, affected in Paf1C-dependent RNAPII phosphorylation, shows higher RNAPII phosphorylation, both at Ser2 and Ser5 in western blots. On the other side, RNAPII-Ser2P-ChIP shows a redistribution from 3' to 5', mainly over LDL3-dependent loci in the elf8 mutant. To explain this contradictory result, the authors come up with the explanation that there is an increase in nascent RNA, which they observe using RNA-chromatin IP. Their interpretation is that there is a delay in transcriptional elongation. However, this does not result in changes in mRNA level, which ultimately questions the significance of these results.

Another point is that LDL3 function is inferred mainly indirectly through H3K4me2 ChIP-seq data, assuming that the increase in H3K4me2 are caused by a loss in LDL3 activity, which in turn might reflect RNAPII activity due to direct, phosphorylation-dependent interaction. It would be important to show LDL3 binding in a RNAIIP-dependent manner directly. There might be technical difficulties for this, but it's a missing piece to the overall picture. This would also be important to shed light on the somewhat contradictory result with the elf8 mutant that leads to elevated RNAIIP and should therefore show increased LDL3 binding. Yet, H3K4me2 is rather increased in the elf8 mutant. This contrasts with the cdkf;1 mutant, showing both decrease in RNAIIP and increase in H3K4me2 as one would expect. It should be noted however, that global mRNA transcript levels do not change in cdkf;1 mutants, but rather affect sRNA biogenesis (Hajheidari et al, Plant Cell 2012). Moreover, cdkf;1 mutants have strong developmental phenotypes, which makes it difficult to directly compare it with wild type or other mutants as the observed changes in chromatin modifications might be indirect. Taken together, LDL3-ChIP in wild type and the different mutants would provide direct evidence for the observed changes in the chromatin landscape.

On a more general note, it should be kept in mind that H3K4 cannot exist in different methylation forms (me0, me1, me2, me3) at the same histone, though the authors observe unchanged levels of H3K4me3 in ldl3 mutants and the patterns overlaps to quite some extent with that of H3K4me2 (skewed towards the TSS). This indicates that H3K4me2 and me3 are deposited at different sites and are largely independent of each other, which is a bit puzzling given that these histone modifications are associated with gene silencing and activation, respectively.

Further points are listed below:

Line 105ff: What is the overlap of tissue-specific H3K4me2 (shoot, root, callus)?

Line 260ff: "we observed a positive correlation between H3K4me2 and transcription levels in the ldl3 mutant". I am not sure if this statement can be made based on a coefficient value of R=0.215, which is rather low.

Line 264ff: "LDL3 demethylates H3K4me2 cotranscriptionally to establish the negative correlation between transcription and H3K4me2." This is an awkward formulation. The activity of the LDL3 H3K4me2 demethylase is correlated with transcriptional activity and in turn H3K4me2 is present (and correlates with) transcriptionally silent genes.

Line 312ff: "LDL3 establishes the plant-specific negative correlation between H3K4me2 and transcription by the transcription-driven demethylation of H3K4." Again, this formulation is a bit awkward as LDL3 is removing H3K4me2 and it is not explored how the H3K4me2 modification is brought about.

Line 328ff: "active demethylation by LDL3, driven by transcriptional elongation, functions as a memory to control developmental plasticity and robust gene control in plants." Not sure if one might infer a memory function for H3K4me2 demethylation as this takes place together with transcription and is regulated by RNAII-Polymerase phosphorylation. To substantiate the assumption of an epigenetic memory function one would need to show a priming function by H3K4me1 to regain transcriptional activity more efficiently than without H3K4me1.

Figure 6A: The last numbers in the graphs are cut.

Point-by-point response to the referees' comments.

(Referees' comments are shown by black and our responses are shown by green.)

We thank the Editor and Referees for the constructive comments. We incorporated most of the comments, which improved the manuscript very much.

Referee #1:

Previous papers showed that plants use LDL3 to co-transcriptionally remove H3K4me2 within transcribed regions, reversing the typically positive correlation between transcription and H3K4 methylation states. This paper attempts to correlate this effect with other features of transcription, concluding that both CTD Serine 2 phosphorylation and the PAF1 Complex contribute to LD3 function. The paper presents good evidence for LDL3 binding to the RNA pol II CTD during elongation. Overall, the story seems pretty consistent.

We thank the Referee #1 for the positive evaluation of the manuscript.

However, some things don't quite make sense, perhaps due to the way the results are analyzed.

Major concerns:

1. Many of the mutations tested here are likely to have systemic, rather than gene-specific, effects. It is now well-recognized that any genomic technique (ChIP-seq, nascent RNA seq, etc) wil miss these effects without some type of spiked-in normalization method, typically a corresponding sample from some other organism that can go through the entire library preparation but be distinguished at the computational level (see Chen et al., Mol Cell Biol. 2016 36(5): 662-667; Loven et al. Cell 151:476-482. doi: 10.1016/j.cell.2012.10.012). Unfortunately, this paper only normalizes by "reads per million", which assumes that any changes are localized and occur against a background where most gene profiles do not change. That seems unlikely here. The proper thing would be to re-do the experiments here with proper spike-ins, but obviously that would be a huge amount of work. There are a few methods around for using other genomic features as internal normalizations. I suggest the authors talk to some

more experienced genomics labs to see if they can find a method to substitute.

We thank the Referee #1 for constructive suggestions. As the Referee pointed out, ChIP-seq analyses reveal relative, rather than absolute levels of the epigenetic marks. In order to access the absolute level of the epigenetic marks, spike-in or Western blotting analyses would complement the ChIP-seq results. We therefore estimated the global levels of the H3K4me1/me2/me3 by Western blotting (n=3; Fig. 2E, Appendix Fig. S7D).

2. One very obvious place where this problem is occurring is shown in Fig S2. In other organisms, knocking out H2Bub completely blocks H3K4 methylation. The structure of Set1/COMPASS shows exactly how the ubiquitin allosterically activates the methyltransferase. Yet the ChIP here shows almost no effect. I would like to see the western for H3K4me2, since I find this very hard to believe. Either Arabidopsis K4 methyltransferase is completely different from both yeast and mammals in not sensing H2Bub, or improper normalization of the ChIP-seq is giving a misleading result. This same effect could be affecting the other genomic experiments as well.

Actually, Western blotting results of Arabidopsis H2Bub mutants has been shown in multiple previous publications (Fig. 6a of Zhao et al. 2019; Fig. 5A of Dhawan et al. 2009; Fiorucci et al. 2019). In these reports, no significant changes in H3K4me were detected in the H2Bub mutant. As the Referee #1 pointed out, it is reported in yeast and mammals that knocking out of H2Bub blocks H3K4 methylation. Very interestingly, however, the control seems different in *Arabidopsis* (Zhao et al. 2019; Dhawan et al. 2009; Fiorucci et al. 2019) and our ChIP-seq results (Fig. S3) are consistent with these results. We added citations of these previous publications.

3. Similarly, it's odd that Paf1C mutants would produce more H3K4me2, since overall transcription is probably down. What happens to H3K4me3? It may be more likely that me2 increases because there is less me3, not because of an effect on the demethylase. Or the normalization may be hiding effects.

Our ChIP-seq results of the *elf8* mutant show that the increase of H3K4me2 in the LDL3 targets is not associated with decrease in H3K4me3 (Appendix Fig. S7). Instead, that is associated with decrease in H3K4me1, suggesting that increases of H3K4me2 in the *elf8* mutant as a compromised function of

H3K4me2 demethylase LDL3. In addition, the hyper-H3K4me2 region in *elf8* is the 3’ half of the genes, not in the 5‘ half of the genes where H3K4me3 localizes. Our western blotting results also show that global H3K4me3 level is unaffected by the *elf8* mutation, which is consistent with a previous publication (Oh et al 2004).

4. It's interesting that some of the mutants seem to shift the distribution of K4me2 along genes. However, the assignment of LDL3 "target" genes and other mutation effects is based on total H3K4me2 read counts (as in Fig 1A). Isn't it possible that may target genes could be missed if the overall amount of H3K4me2 remains the same but the peaks shift considerably? In other words, could there be genes where changes are qualitative, but not quantitative?

As the Referee #1 pointed out, our screening of the LDL3 target genes is based on the total level of H3K4me2. We agree with the Referee that the spectrum of the target genes could be different when the analyses include genes with shift of the peaks without changes in the total levels of H3K4me2. We therefore screened LDL3 target genes using differential peak calling of H3K4me2 between the WT and the *ldl3* mutants (Appendix Fig. S2). The shifts of the peaks are also shown in Appendix Fig. S4C. The spectra of the LDL3-target genes identified in these two approaches are surprisingly similar (Appendix Fig. S2A), and results of random forest analyses are also similar (Appendix Fig. S2C). These additional analyses confirmed the robustness of our approach. We thank the Referee for the suggestion.

5. Since the authors have a FLAG-tagged LDL3 construct and show they can co-IP RNA pol II, I was surprised they did not directly look at LDL3 recruitment in the various mutants using ChIP-seq. For example, by their model, mutating the CTD kinase should lead to loss of LDL3 signal. This would be an important test of the model.

As suggested by the Referee #1 and #3, we examined LDL3 localization in the *elf8* mutant by ChIP-seq. Indeed, we could detect decrease in the localization of LDL3 in the *elf8* mutant, and the affected regions correspond to the regions with increase in H3K4me2 (Fig. 5A, B). These new results suggest the possibility that Paf1C regulates H3K4me2 by controlling localization of LDL3. We thank the Referees, as the results strengthened the manuscript very much.

6. I don't understand the assertion that H3K4me2 acts as a "memory"? In what sense? "Transcriptional memory" is typically defined in terms of gene induction, which in some cases produces a different response between the first and second inductions. But nothing like that is tested here. Please define what you mean by "memory" and how you would assay it. Otherwise, if this is just a vague idea, the term "memory" should be dropped because it will be confused with the existing literature.

Previous work suggests that LDL3 function contributes to gene induction during regeneration (Ishihara et al. 2019). However, as the Referee pointed out, those types of analyses are not included in this manuscript. We also agree that the term “memory” could be confusing. We therefore changed the expression throughout the manuscript.

Minor issues:

1. Line 163. Several papers in yeast have shown that H3K36 methylation is dependent on Paf1C function (for example, Krogan et al. MCB 2003).

As suggested, we added a citation of Krogan et al., 2003.

2. Fig6. Given that K4 methylation is transcription-directed, the correlation coefficients are surprisingly low (all below 0.2) for K4me2 and me1 (especially compared to K36me3). Any idea why?

We have previously shown that H3K4 methyltransferases can be classified into transcription-dependent and transcription-independent types (Oya et al. 2022). It is likely that transcription-independent methyltransferases as well as demethylases weaken the correlation. We agree that the correlation levels are low, but most of them are highly significant. We added p-values to Fig. 6A. We think it important to show that the effect of the H3K4me2 demethylation pathway by LDL3 very significantly alters the correlation between transcription and H3K4me2.

3. The introduction mentions that it seems paradoxical that H3K4me2 could be repressive for transcription. In fact, there are several papers showing K4me2 in yeast recruits the Set3 HDAC, which can suppress elongation and internal cryptic initiation sites.

We added a citation of Kim et al. 2012. We thank the Referee #1 for the suggestion.

Referee #2:

This study provides interesting data regarding H3K4 methylation and transcription, including genome-wide distribution of H3K4me2, Pol II and chrRNA in an important set of mutants. It also demonstrates the direct interaction of LDL3 with phosphorylated Pol II.

We thank the Referee #2 for the positive evaluation of the manuscript.

However, there are some concerns with interpretation of the data and emphasis of the conclusions that might confuse readers.

Major concerns:

The authors emphasise the fact that H3K4me2 correlates negatively with transcription. Any correlation of H3K4me2 enrichment with repression could just be a consequence of the transition to H3K4me3 being less likely in a repressed state - i.e. less H3K4me3 means more H3K4me2. Conversely, in an activated state, more H3K4me3 means less H3K4me2. The is in fact observed in some of the cited data (Wang et al., 2022). Essentially, the three modification states - me1, me2, me3 must show some degree of mutual exclusivity since they are on the same residue. And this would have nothing to do with a repressive role for H3K4me2.

Including the H3K4me3 methyltransferase mutant would benefit this study (H3K4me2 accumulation and genetic analyses with cdkf;1 and elf8). ldl3, cdkf1, elf8, show increased H3K4me2 associated with reduced H3K4me1, reinforcing the mutual exclusivity of methylated states. This clarifies work in Ishihara et al., 2019, where changes in H3K4me2 between WT and ldl3 showed no clear effect on either K4me1 or K4me3.

As the Referee #2 pointed out, we show here that the increases in H3K4me2 by *ldl3*, *cdkf1*, and *elf8* mutations are associated with decreases in H3K4me1,

rather than H3K4me3. In addition, the hyper-H3K4me2 region in *elf8* is the 3' half of the genes, not in the 5' half of the genes where H3K4me3 localizes. Thus, while H3K4me0/1/2/3 are mutually exclusive, the H3K4me2 dynamics we are examining here does not reflect the indirect effect of H3K4me3.

Different modification states of H3K4 can behave differently in different parts of the gene. This is also seen in the genome wide data presented in Ishihara et al., 2019. It is important to expand the presentation/discussion of this when drawing conclusions about different methylation levels from genome wide correlations.

We examined the correlation between H3K4me and transcription after dividing the transcription start site (TSS) region and the gene body region (Appendix Fig. S14, 15). While H3K4me3 and H3K4me2/me1 are indeed exclusive near the TSS, the correlation remains unchanged in this region in *ldl3*. On the other hand, in the gene body region, the negative correlation between H3K4me2 and transcription was altered in *ldl3*. Based on these and other unpublished results we consider that H3K4me2 in the gene body, rather than near TSS, plays a repressive role. Inclusion of the analyses after separating TSS and other region certainly has strengthened the manuscript and we thank the Referee #2 for this constructive suggestion.

The authors claim throughout the manuscript that demethylation by LDL3 has an important role in chromatin state memory (lines 36-38, 98-10 and 327-330). What they mean about the memory aspect of H3K4me2 is quite speculative. Is it about priming/poising of transcription? Or is it about mitotic inheritance? Mitotic inheritance (across DNA replication) would require a feedback mechanism that is not addressed. For example, a recent paper tries to demonstrate mitotic inheritance of H3K4me2 in S. cerevisiae through read-write feedback (https://doi.org/10.7554/eLife.77646). This provides short term memory of transcriptional activation (they see it as an activating mark). The authors should cite this paper when they discuss the memory aspect.

As the Referee #2 pointed out, the memory aspect of H3K4me2 is still at a speculative stage. Previous work suggests that LDL3 function contributes to gene induction during regeneration (Ishihara et al. 2019), but we did not include the related data here. Considering the potential confusion caused by the term "memory," we changed the expression throughout the manuscript.

It is quite interesting that they see increased H3K4me2 in elf8, where elongation is retarded, and consequently RNAP II hyper-accumulates. This would be quite consistent with a model where more time spent by RNAP II in a region increases the probability of adding more methylation at H3K4 - i.e. faster elongation only allows a short time window sufficient to add me1, while slower elongation allows more time to transition to me2. A model along these lines is proposed by Fong et al., 2017 proposes for H3K36me3 in mammals (https://doi.org/10.1016/j.molcel.2017.04.016). In the current manuscript, the authors suggest that the H3K4me2 accumulation in elf8 is mainly a consequence of disrupted LDL3 function. However, there is a large subset of "LDL3 targets", where the ldl3 elf8 double mutant shows higher H3K4me2 than the ldl3 single mutant (Cluster 5, Figure S10 (A)). The behaviour of these targets is consistent with an "increased dwell-time" model for elf8 as described above. It would be useful if the authors address this aspect more explicitly, perhaps by comparing changes in RNAP II occupancy in elf8 between.

We thank the Referee #2 for the suggestion. We added the heatmap for H3K4me2 changes in *ldl3 elf8* and compared the changes to that in RNAPII Ser2P by the *elf8* mutation (Fig. 5A). In the genes with further elevation of K4me2 in *ldl3 elf8* than in *ldl3*, the *elf8* mutant shows elevation of RNAPII Ser2P, which we believe reflects transcriptional retardation. We retried clustering with this heatmap (Appendix Fig. S12A). In the cluster 1, transcription is retarded by the *elf8* mutation, which is associated with further H3K4me2 elevation. This observation is consistent with the model of previous studies where more time spent by RNAP II in a region increases the probability of adding more methylation at H3K4 (Soares et al. 2017; Fong et al. 2017). We added these discussions to the manuscript.

It is not clear what the overall model is - i.e. how the authors reconcile the insights from their last paper (Oya et al., 2022) on H3K4 methyltransferases. They should address more clearly how they think H3K4me2 is added. Is the addition co-transcriptional too or mainly independent of transcription as in (https://doi.org/10.7554/eLife.77646)?

We have previously identified ATXR3 and ATX3/4/5 as H3K4 methyltransferases which contribute to H3K4me2/me3 (Oya et al. 2022). The *atxr3* mutants showed a weaker positive transcriptional correlation with H3K4me2 in the gene body (Appendix Fig. S16), so we consider that ATXR3 is

depositing H3K4me2 cotranscriptionally.

Minor comments:

Showing snapshots of the sequencing coverage or aligned reads in different loci would benefit the manuscript. This would provide particular examples showing the quality of the data and reveal interesting features that engage the reader such as promoter-proximal pausing, links to splicing, termination/readthrough etc...

As suggested, we added screenshots of ChIP-seq signals to the figure (Appendix Fig. S4, S7, S10, S11).

The authors too often refer to changes in H3K4me2 as LDL3 function (without strong functional support). For example, in lines 28-30 "Here, we show that LDL3-mediated H3K4me2 demethylation depends on (...)" or lines 208-210: "These results suggest that although phosphorylation of RNAPII is necessary for LDL3 function, elf8 mutation affects LDL3 function through a different pathway." It is recommended to remove the reference to LDL3 in these phrases, changing to for example "changes in H3K4me2".

As we detected changes in H3K4me2 in the mutant with compromised LDL3 gene function, we regard it reasonable to refer to those changes in H3K4me2 as LDL3-mediated. The *elf8* and *cdkf;1* mutants mimic this change in H3K4me2. The connections were further substantiated by the results to show binding of LDL3 to phosphorylated RNAP2 (Fig. 3) and new results showing effects of Pa1C to localization of LDL3 protein (Fig. 5, Appendix Fig. S11). We would like to keep those expressions, as that would make the text easier to read.

Fig. 5E - The genetic analysis of ldl3 and elf8 is important to support the claims in the manuscript. It would help to add the heatmap for H3K4me2 in ldl3 elf8 / WT as in most of the other figures (sorted by changes in H3K4me2 in ldl3 single).

As suggested, we added the heatmap for H3K4me2 changes in *ldl3 elf8* (Fig. 5D, Appendix Fig. S12). Thank you for your suggestion.

Fig. 5E - elf8 is missing in the figure legend: "(E) H3K4me2 levels in ldl3 elf8 double mutant compared with WT (top) or (bottom).".

We corrected the error. We thank the Referee for pointing that out.

Editing is required in Results section (lines 257-272) and Fig 6A, particularly (lines 263-264): "These results suggest that LDL3 disrupts the positive correlation of transcription and H3K4me2 by removing H3K4me2 from transcribed genes."
The differences between the two left upper and lower plots in Fig 6A may reflect changes in H3K4me2 levels and therefore not very informative. Above all, the plots show no correlation (positive or negative) between H3K4me2 and chrRNA (Spearman's correlation p very close to zero). In this regard, changes must be made to lines 33-34 from the Abstract: "Importantly, the negative correlation between H3K4me2 and transcription is disrupted in the ldl3 mutant".

We agree with the Referee that the correlation levels are low, but most of them are highly significant. We added p-values to Fig. 6. We also agree that the expression “disrupt” could be too strong and modified the expression. Nonetheless, we believe that the impact of LDL3 to the genome-wide patterning of H3K4me1/2 is very significant, especially in the gene body (Appendix Fig. S14, 15).

Referee #3:

The manuscript "Cotranscriptional demethylation induces global loss of H3K4me2 from active genes in Arabidopsis" by Shusei Mori and co-workers describes the interaction between the histone H3 lysine 4 dimethyl (H3K4me2) demethylase LDL3 with RNA-polymerase II, which seems dependent on the phosphorylation state of the latter. Indeed, mutations in factors that are required for proper RNAPII phosphorylation, involved in transcriptional progression, increase H3K4me2 level over LDL3 target genes. In contrast to H3K4me1 and K4me3, H3K4me2 is negatively associated with transcriptional activity. As a consequence, interfering with RNAPII phosphorylation leads to an increase in chromatin-associated RNA-accumulation over the affected loci, but not in free mRNA transcripts.

This is an interesting work that uncovers novel regulatory functions of chromatin-mediated transcriptional control that is potentially of high general interest to a large scientific community. The experiments seem sound and the manuscript is well written.

We thank the Referee #3 for the very positive evaluation.

I have to admit that there's a certain complexity and some puzzling results that make this a demanding lecture at times. Several observations fall into place, but others are somewhat contradictory. For instance, contrary to the expectation, the elf8 mutant, affected in Paf1C-dependent RNAPII phosphorylation, shows higher RNAPII phosphorylation, both at Ser2 and Ser5 in western blots.

We agree that some of the results are different from those expected from the simplest model. As the Referee #3 pointed out, our results suggest that *cdkf;1* and *elf8* mutations affect the *LDL3*-mediated H3K4me2 demethylation through distinct pathways, which is unexpected and makes the story very intriguing. Although unexpected, all these results are consistent and convincing, we believe, as we explain below.

On the other side, RNAPII-Ser2P-ChIP shows a redistribution from 3' to 5', mainly over LDL3-dependent loci in the elf8 mutant. To explain this contradictory result, the authors come up with the explanation that there is an increase in nascent RNA, which they observe using RNA-chromatin IP. Their interpretation is that there is a delay in transcriptional elongation.

As the Referee #3 pointed out, our chromatin-bound RNA sequencing results suggest that *elf8* mutation results in delay in transcriptional elongation, which is consistent with the function of ELF8 as a component of Paf1C, transcription elongation complex.

However, this does not result in changes in mRNA level, which ultimately questions the significance of these results.

We identified Differentially expressed genes (DEGs) in *elf8* using mRNA-seq data (Fig. R1A). The metaplot displays averaged results and does not provide individual results. However, in fact, there are significant alterations in the expression of numerous genes. Furthermore, there is a positive correlation

between the changes in chrRNA and mRNA in the *elf8* mutant (Fig. R1B), supporting the quality of our chrRNA and mRNA data. Our conclusion from these analyses is that the detected effect of elf8 on transcription dynamics in gene body using chrRNA is not due to the difference of total amount of steady-state mature mRNA. However, mRNA levels of a small number of genes could be affected due to the transcription retardation, which could ultimately lead to a global transcriptional change.

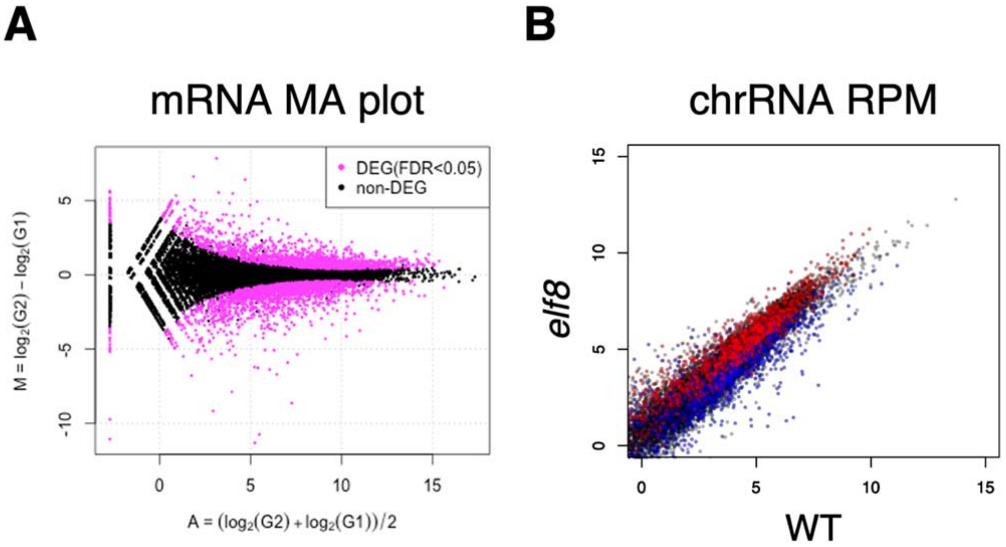

Fig. R1 (A) Differentially expressed genes (DEGs) are identified in the *elf8* mutant. (B) Scatter plot showing the comparisons of chrRNA level between WT and *elf8.* Red dots represent up-regulated DEGs and blue dots represent down-regulated DEGs.

We believe that all these results are consistent and convincing.

Another point is that LDL3 function is inferred mainly indirectly through H3K4me2 ChIP-seq data, assuming that the increase in H3K4me2 are caused by a loss in LDL3 activity, which in turn might reflect RNAPII activity due to direct, phosphorylation-dependent interaction. It would be important to show LDL3 binding in a RNAIIP-dependent manner directly. There might be technical difficulties for this, but it's a missing piece to the overall picture. This would also be important to shed light on the somewhat contradictory result with the elf8 mutant that leads to elevated RNAIIP and should therefore show increased

LDL3 binding. Yet, H3K4me2 is rather increased in the elf8 mutant. This contrasts with the cdkf;1 mutant, showing both decrease in RNAIIP and increase in H3K4me2 as one would expect. It should be noted however, that global mRNA transcript levels do not change in cdkf;1 mutants, but rather affect sRNA biogenesis (Hajheidari et al, Plant Cell 2012). Moreover, cdkf;1 mutants have strong developmental phenotypes, which makes it difficult to directly compare it with wild type or other mutants as the observed changes in chromatin modifications might be indirect. Taken together, LDL3-ChIP in wild type and the different mutants would provide direct evidence for the observed changes in the chromatin landscape.

We agree with the Referee #3 and #1 that a key experiment would be to examine localization of LDL3 protein in the *elf8* mutant. As suggested by the Referees, we examined LDL3 localization in the *elf8* mutant by ChIP-seq. Indeed, we could detect decrease in the localization of LDL3 in the *elf8* mutant, and the affected regions correspond to the regions with increase in H3K4me2 (Fig. 5A, B). These new results suggest the possibility that Paf1C regulates H3K4me2 by controlling localization of LDL3. We thank the Referees, as the results strengthened the manuscript very much.

On a more general note, it should be kept in mind that H3K4 cannot exist in different methylation forms (me0, me1, me2, me3) at the same histone, though the authors observe unchanged levels of H3K4me3 in ldl3 mutants and the patterns overlaps to quite some extent with that of H3K4me2 (skewed towards the TSS). This indicates that H3K4me2 and me3 are deposited at different sites and are largely independent of each other, which is a bit puzzling given that these histone modifications are associated with gene silencing and activation, respectively.

According to the ChIP-seq results, hyper-H3K4me2 region in *ldl3* is the 3' half of the genes, not in the 5' half of the genes where H3K4me3 accumulates (Appendix Fig. S7). Therefore, we propose that LDL3 demethylates H3K4me2 at the gene body where RNAPII is undergoing transcriptional elongation, independent of H3K4me3 near the TSS. We also added characterization of the relationship between transcription and H3K4me1/2/3 after separation of TSS and gene body (Appendix Figs S14, S15).

Further points are listed below:

Line 105ff: What is the overlap of tissue-specific H3K4me2 (shoot, root, callus)?

We added the heatmap for H3K4me2 changes in *ldl3* in shoot, root, and callus (Appendix Figs S1C). LDL3 targets are common among those tissues, but H3K4me2 changes are greater in callus, suggesting that LDL3 activity is higher in callus. This is consistent with the previous report that LDL3 transcription was up-regulated during callus formation (Ishihara et al. 2019).

Line 260ff: "we observed a positive correlation between H3K4me2 and transcription levels in the ldl3 mutant". I am not sure if this statement can be made based on a coefficient value of R=0.215, which is rather low.

We agree that the coefficient value is low, but the correlation is highly significant. We added the p-values to Fig. 6. The effect of the H3K4me2 demethylation pathway by LDL3 alters the correlation between transcription and H3K4me2 in a highly significant manner.

Line 264ff: "LDL3 demethylates H3K4me2 cotranscriptionally to establish the negative correlation between transcription and H3K4me2." This is an awkward formulation. The activity of the LDL3 H3K4me2 demethylase is correlated with transcriptional activity and in turn H3K4me2 is present (and correlates with) transcriptionally silent genes.

We changed the expression to "LDL3 demethylates H3K4me2 cotranscriptionally in gene bodies, resulting in the establishment of negative correlation between H3K4me2 and transcription level"

Line 312ff: "LDL3 establishes the plant-specific negative correlation between H3K4me2 and transcription by the transcription-driven demethylation of H3K4." Again, this formulation is a bit awkward as LDL3 is removing H3K4me2 and it is not explored how the H3K4me2 modification is brought about.

We modified the expression to "LDL3 contributes to the plant-specific negative correlation between H3K4me2 and transcription by the transcription-driven demethylation of H3K4." In regard to the machinery to bring about H3K4me2, we have previously identified ATXR3 and ATX3/4/5 as H3K4 methyltransferases which contribute to H3K4me2/me3 (Oya et al. 2022). The

atxr3 mutants showed a weaker positive transcriptional correlation with H3K4me2 in the gene body (Appendix Fig. S16), so we consider that ATXR3 deposits H3K4me2 cotranscriptionally. That is, we think that the positive correlation between H3K4me2 and transcription observed in the *ldl3* mutant is likely generated by ATXR3. We added this explanation to the manuscript.

Line 328ff: "active demethylation by LDL3, driven by transcriptional elongation, functions as a memory to control developmental plasticity and robust gene control in plants." Not sure if one might infer a memory function for H3K4me2 demethylation as this takes place together with transcription and is regulated by RNAII-Polymerase phosphorylation. To substantiate the assumption of an epigenetic memory function one would need to show a priming function by H3K4me1 to regain transcriptional activity more efficiently than without H3K4me1.

As the Referee #3 pointed out, we have not been able to demonstrate this hypothesis in this paper. Considering the potential confusion caused by the term "memory," we excluded it.

Figure 6A: The last numbers in the graphs are cut.

We corrected the error. We thank the Referee for pointing that out.

Dear Tetsuji,

Thank you for submitting your revised manuscript for consideration by the EMBO Journal. It has now been seen again by the three referees who originally looked at the work; their comments are attached below. As you will see, all three referees remain in favour of publication; however, as you will see, Referees 2 and 3 raise some concerns over your interpretation of the data.

I would like to invite you to address these concerns in a second round of minor revisions. If you would find it helpful to discuss these reports over Zoom, I will be available next week (I am currently on holiday).

When preparing your letter of response to the referees' comments, please bear in mind that this will form part of the Review Process File, and will therefore be available online to the community. For more details on our Transparent Editorial Process, please visit our website: https://www.embopress.org/page/journal/14602075/authorguide#transparentprocess

Thank you for the opportunity to consider your work for publication. I look forward to your revision.

Best wishes,

William,

William Teale, PhD
Editor
The EMBO Journal
w.teale@embojournal.org

Instructions for preparing your revised manuscript:

Please check that the title and abstract of the manuscript are brief, yet explicit, even to non-specialists.

When assembling figures, please refer to our figure preparation guideline in order to ensure proper formatting and readability in print as well as on screen:
https://bit.ly/EMBOPressFigurePreparationGuideline
See also figure legend guidelines: https://www.embopress.org/page/journal/14602075/authorguide#figureformat

IMPORTANT: When you send the revision we will require
- a point-by-point response to the referees' comments, with a detailed description of the changes made (as a word file).
- a word file of the manuscript text.
- individual production quality figure files (one file per figure)
- a complete author checklist, which you can download from our author guidelines (https://www.embopress.org/page/journal/14602075/authorguide).
- Expanded View files (replacing Supplementary Information)
Please see out instructions to authors
https://www.embopress.org/page/journal/14602075/authorguide#expandedview

Please remember: Digital image enhancement is acceptable practice, as long as it accurately represents the original data and conforms to community standards. If a figure has been subjected to significant electronic manipulation, this must be noted in the figure legend or in the 'Materials and Methods' section. The editors reserve the right to request original versions of figures and the original images that were used to assemble the figure.

Further information is available in our Guide For Authors: https://www.embopress.org/page/journal/14602075/authorguide

We realize that it is difficult to revise to a specific deadline. In the interest of protecting the conceptual advance provided by the work, we recommend a revision within 3 months (21st Nov 2023). Please discuss the revision progress ahead of this time with the editor if you require more time to complete the revisions. Use the link below to submit your revision:

https://emboj.msubmit.net/cgi-bin/main.plex

Referee #1:

The authors have addressed my comments. I'm surprised that the plant system for modulating H3K4 methylation seems to be quite different than in mammals or yeast, but that makes the paper interesting and not simply confirmatory of other systems.

Referee #2:

The finding that a demethylase travels along with Pol II linking transcription and chromatin changes is very interesting. However, the different activities of the LDL family and difficulty of simple conclusions when all the feedbacks are not understood means some of the statement in the manuscripts will lead to confusion in the field. For example:
-H3K4me2 in plants functions as an epigenetic mark of the inactive chromatin state
-the overall conclusion that H3K4me2 is a repressive mark
These won't be helpful. H3K4me2 is likely to be an intermediate state (eg. reduced H3K4me3) so not really directing repression. Overall, the discussion of switching of negative/positive correlations is still confusing.

A faint band in the anti-total pol II WB suggests that possibly other LDLs might associate with PolII but loosely.

mRNA-seq section in Methods duplicated

Referee #3:

The revised version of the manuscript has improved and my comments have been properly addressed. The LDL3 ChIP brings further evidence that this demethylase is indeed responsible for the reduction of H3K4me2. Yet, the new data are also adding some more confusion:

1) The overall level of H3K4me2 as shown in the Western blots is elevated in ldl3 mutants but not in the cdkf;1 or the elf8 mutants. This contrasts with the increased level of H3K4me3 in all three of these mutants compared to wild type, which is, however, not detected in the ChIP-seq experiments. How do the authors explain these seemingly contradictory results?

2) Moreover, although it fits the model that Paf1C is responsible for LDL3-dependent demethylation from H3K4m2 to me1, it is still puzzling in the context of the elevated RNAPII phosphorylation in the elf8 mutant. The authors state that the comparable effect on H3K4me2 in cdkf;1 and elf8 mutant is brought about by different mechanisms, one dependent and the other independent of RNAPII phosphorylation, though they do not provide a mechanistic explanation. If the interaction between LDL3 and RNAPII seems to be enhanced by the phosphorylation of the latter, why does this not result in increased LDL3 binding and hence removal of H3K4me2 but instead leads to H3K4me2 increase? Is it possible that the LDL3 RNAPII interaction is mediated by Paf1C and not by direct contact between LDL3 and the RNA-PolII complex? The pull-down experiments using whole plant protein extracts presented here do not allow to distinguish these two possibilities.

3) Last, I am still not very persuaded that "LDL3 alters the positive correlation between H3K4me2 and transcription to negative correlation", but rather that H3K4me2 is simply not (functionally or directly) associated with transcription.

Minor points are listed below:

Line 227: "Ser2/total RNAPII" - it should say Ser2P/total RNAPII I guess.

Line 263: "[] a subset of genes showed higher H3K4me2 levels []". It would be helpful to give numbers or the percentage(s) of the affected genes, respectively.

Line 271: "LDL3 alters the positive correlation between H3K4me2 and transcription to negative correlation". Although this conclusion might be formally correct, this interpretation holds on a rather weak correlation of H3K4me2 level and expression strength. The results of the ldl3 mutant actually reveals that H3K4me2 function is not associated with transcription instead of changing a reversion of its correlation with expression (see also the general comments above).

Line 466f: The description of the RNA-seq experiments appears twice.

Point-by-point response to the Referees' comments

Referees' comments are shown by black and our responses are shown by blue.

Referee #1:

The authors have addressed my comments. I'm surprised that the plant system for modulating H3K4 methylation seems to be quite different than in mammals or yeast, but that makes the paper interesting and not simply confirmatory of other systems.

Referee #2:

The finding that a demethylase travels along with Pol II linking transcription and chromatin changes is very interesting. However, the different activities of the LDL family and difficulty of simple conclusions when all the feedbacks are not understood means some of the statement in the manuscripts will lead to confusion in the field. For example:
-H3K4me2 in plants functions as an epigenetic mark of the inactive chromatin state
-the overall conclusion that H3K4me2 is a repressive mark
These won't be helpful. H3K4me2 is likely to be an intermediate state (eg. reduced H3K4me3) so not really directing repression. Overall, the discussion of switching of negative/positive correlations is still confusing.

We agree that the expression “the inactive chromatin state” or “a repressive mark” could be too strong or potentially confusing. We changed the expression to “H3K4me2 negatively affects transcription”. In fact, hyper-H3K4me2 genes in *ldl3* tend to decrease the expression (Appendix Fig. S2). We added this new figure. This observation is also consistent with other reports (Line 110-114). We also toned down the other expressions to make them less confusing.

A faint band in the anti-total pol II WB suggests that possibly other LDLs might associate with PolII but loosely.

As suggested, the difference between LDL3 and the other LDLs is not so obvious for anti-total pol II than for anti-phosphorylated pol II. We modified that part. Actually, the weak signal is consistent with our previous results (Inagaki et al, 2021) suggesting that FLD colocalizes with pol II. FLD may also bind to pol II, even though the binding is much weaker than that of LDL3.

mRNA-seq section in Methods duplicated

We corrected the error. We thank the Referee for pointing that out.

Referee #3:

The revised version of the manuscript has improved and my comments have been properly addressed. The LDL3 ChIP brings further evidence that this demethylase is indeed responsible for the reduction of H3K4me2. Yet, the new data are also adding some more confusion:

1) The overall level of H3K4me2 as shown in the Western blots is elevated in ldl3 mutants but not in the cdkf;1 or the elf8 mutants. This contrasts with the increased level of H3K4me3 in all three of these mutants compared to wild type, which is, however, not detected in the ChIP-seq experiments. How do the authors explain these seemingly contradictory results?

From the ChIP-seq results, the degree of H3K4me2 elevation in *elf8* or *cdkf;*1 is small compared to that in *ldl3*; we consider that is why the elevation in *elf8* or *cdkf;1* could not be detected by Western blots. Looking at violin plots summarising the ChIP-seq results (Appendix Fig. S8D), H3K4me3 levels were increased and H3K4me1 levels were decreased in the LDL3 target genes in the mutants, and this trend is consistent with the WB results. More importantly, an increase in H3K4me2 in the *ldl3* mutant is associated with a decrease in H3K4me1, not H3K4me3. To make it clearer, we added this figure and a brief explanation.

2) Moreover, although it fits the model that Paf1C is responsible for LDL3-dependent demethylation from H3K4m2 to me1, it is still puzzling in the

context of the elevated RNAPII phosphorylation in the elf8 mutant. The authors state that the comparable effect on H3K4me2 in cdkf;1 and elf8 mutant is brought about by different mechanisms, one dependent and the other independent of RNAPII phosphorylation, though they do not provide a mechanistic explanation. If the interaction between LDL3 and RNAPII seems to be enhanced by the phosphorylation of the latter, why does this not result in increased LDL3 binding and hence removal of H3K4me2 but instead leads to H3K4me2 increase? Is it possible that the LDL3 RNAPII interaction is mediated by Paf1C and not by direct contact between LDL3 and the RNA-PolII complex? The pull-down experiments using whole plant protein extracts presented here do not allow to distinguish these two possibilities.

We completely agree that the in vivo binding of LDL3 RNAPII can be mediated by other protein(s). It would be very interesting if that is mediated by Paf1C. Our new results of the LDL3ΔSRI Co-IP results showed that the SRI domain is required for binding to RNAPII (Appendix Fig. S11A, B). The SRI domain is known to bind to phosphorylated CTD of RNAPII (Kizer et al. 2005). This result supports the idea that LDL3 recognizes and binds to phosphorylated RNAPII. Nonetheless, it would be very interesting if the binding depends on Paf1C. We added a suggestion for this possibility (Line 333-335).

3) Last, I am still not very persuaded that "LDL3 alters the positive correlation between H3K4me2 and transcription to negative correlation", but rather that H3K4me2 is simply not (functionally or directly) associated with transcription.

We added the following explanation in the Result section to make the logic clearer (Line 284-288). “Considering that the LDL3 target genes are highly expressed (Fig. 1D) and that LDL3 interacts with RNAPII (Fig. 3A, B), we speculated that LDL3 contributes to the negative correlation between H3K4me2 and transcription (Liu et al. 2019) by removing H3K4me2 from highly transcribed genes. Indeed, the negative correlation was attenuated and even became positive in the ldl3 mutant (Fig. 6A).).“

Minor points are listed below:

Line 227: "Ser2/total RNAPII" - it should say Ser2P/total RNAPII I guess.

We revised this part as suggested.

Line 263: "[] a subset of genes showed higher H3K4me2 levels []". It would be helpful to give numbers or the percentage(s) of the affected genes, respectively.

As suggested, we added the numbers of the genes to the manuscript and the legend.

Line 271: "LDL3 alters the positive correlation between H3K4me2 and transcription to negative correlation". Although this conclusion might be formally correct, this interpretation holds on a rather weak correlation of H3K4me2 level and expression strength. The results of the ldl3 mutant actually reveals that H3K4me2 function is not associated with transcription instead of changing a reversion of its correlation with expression (see also the general comments above).

We changed the description as described above.

Line 466f: The description of the RNA-seq experiments appears twice.

We corrected the error. We thank the Referee for pointing that out.

2nd Revision - Editorial Decision 19th Sep 2023

Dear Tetsuji,

Thank you for addressing the reviewers' comments. In my opinion, you have addressed all concerns satisfactorily; therefore, unless any unexpected issues arise, I will not seek any additional input from the reviewers. Before I can finally accept the manuscript, there are some remaining editorial points which need to be addressed. In this regard, would you please:

acknowledge grants 21H04977, 23H00365 on our online submission system,
rename the Conflict of Interest section the "Disclosure Statement and Competing Interests" statement,
remove the author credit section from the manuscript file,
complete the BLANK SD checklist (which has been uploaded for you to eJP), and zip the SD files, saved in a scheme one figure/folder and then uploaded as .zip files. For example, all the source data files for figure 1 need to be saved in a single folder and this needs to be zipped and then uploaded as "SD figure 1.zip" file,
we can use up to five of the 17 Appendix figures as EV figures; these should be uploaded individually as Figure files, with their legends provided in the manuscript file below the main figure legends. The other Appendix legends need to be part of the Appendix file and removed from ms file , and
move the reference section to before the figure legends.

I look forward to receiving these changes. EMBO Press is an editorially independent publishing platform for the development of EMBO scientific publications.

Best wishes,

William

William Teale, PhD
Editor
The EMBO Journal
w.teale@embojournal.org

Instructions for preparing your revised manuscript:

Please check that the title and abstract of the manuscript are brief, yet explicit, even to non-specialists.

When assembling figures, please refer to our figure preparation guideline in order to ensure proper formatting and readability in print as well as on screen:
https://bit.ly/EMBOPressFigurePreparationGuideline
See also figure legend guidelines: https://www.embopress.org/page/journal/14602075/authorguide#figureformat

IMPORTANT: When you send the revision we will require
- a point-by-point response to the referees' comments, with a detailed description of the changes made (as a word file).
- a word file of the manuscript text.
- individual production quality figure files (one file per figure)
- a complete author checklist, which you can download from our author guidelines (https://www.embopress.org/page/journal/14602075/authorguide).
- Expanded View files (replacing Supplementary Information)
Please see out instructions to authors
https://www.embopress.org/page/journal/14602075/authorguide#expandedview

Please remember: Digital image enhancement is acceptable practice, as long as it accurately represents the original data and conforms to community standards. If a figure has been subjected to significant electronic manipulation, this must be noted in the figure legend or in the 'Materials and Methods' section. The editors reserve the right to request original versions of figures and the original images that were used to assemble the figure.

Further information is available in our Guide For Authors: https://www.embopress.org/page/journal/14602075/authorguide

We realize that it is difficult to revise to a specific deadline. In the interest of protecting the conceptual advance provided by the work, we recommend a revision within 3 months (18th Dec 2023). Please discuss the revision progress ahead of this time with the editor if you require more time to complete the revisions. Use the link below to submit your revision:

All editorial and formatting issues were resolved by the authors.

Dear Tetsuji,

I am pleased to inform you that your manuscript has been accepted for publication in the EMBO Journal.

Congratulations on a thorough and insightful piece of work!

Please note that it is EMBO Journal policy for the transcript of the editorial process (containing referee reports and your response letter) to be published as an online supplement to each paper. If you do NOT want this, you will need to inform the Editorial Office via email immediately. More information is available here: https://www.embopress.org/page/journal/14602075/authorguide#transparentprocess

Your manuscript will be processed for publication in the journal by EMBO Press. Manuscripts in the PDF and electronic editions of The EMBO Journal will be copy edited, and you will be provided with page proofs prior to publication. Please note that supplementary information is not included in the proofs.

You will be contacted by Wiley Author Services to complete licensing and payment information. The required 'Page Charges Authorization Form' is available here: https://www.embopress.org/pb-assets/embo-site/tej_apc.pdf - please download and complete the form and return to embopressproduction@wiley.com

EMBO Press participates in many Publish and Read agreements that allow authors to publish Open Access with reduced/no publication charges. Check your eligibility: https://authorservices.wiley.com/author-resources/Journal-Authors/open-access/affiliation-policies-payments/index.html

Should you be planning a Press Release on your article, please get in contact with embojournal@wiley.com as early as possible, in order to coordinate publication and release dates.

If you have any questions, please do not hesitate to call or email the Editorial Office. Thank you for your contribution to The EMBO Journal.

Best wishes,

William

William Teale, PhD
Editor
The EMBO Journal
w.teale@embojournal.org

** Click here to be directed to your login page: https://emboj.msubmit.net

EMBO Press Author Checklist

Corresponding Author Name: Shusei Mori, Soichi Inagaki, Tetsuji Kakutani
Journal Submitted to: The EMBO Journal
Manuscript Number: EMBOJ-2023-113798

USEFUL LINKS FOR COMPLETING THIS FORM
The EMBO Journal - Author Guidelines
EMBO Reports - Author Guidelines
Molecular Systems Biology - Author Guidelines
EMBO Molecular Medicine - Author Guidelines

Reporting Checklist for Life Science Articles (updated January
This checklist is adapted from Materials Design Analysis Reporting (MDAR) Checklist for Authors. MDAR establishes a minimum set of requirements in transparent reporting in the life sciences (see Statement of Task: 10.31222/osf.io/9sm4x). Please follow the journal's guidelines in preparing your
Please note that a copy of this checklist will be published alongside your article.

Abridged guidelines for figures
1. Data
The data shown in figures should satisfy the following conditions:
- the data were obtained and processed according to the field's best practice and are presented to reflect the results of the experiments in an accurate and unbiased manner.
- ideally, figure panels should include only measurements that are directly comparable to each other and obtained with the same assay.
- plots include clearly labeled error bars for independent experiments and sample sizes. Unless justified, error bars should not be shown for technical
- if n<5, the individual data points from each experiment should be plotted. Any statistical test employed should be justified.
- Source Data should be included to report the data underlying figures according to the guidelines set out in the authorship guidelines on Data

2. Captions
Each figure caption should contain the following information, for each panel where they are relevant:
- a specification of the experimental system investigated (eg cell line, species name).
- the assay(s) and method(s) used to carry out the reported observations and measurements.
- an explicit mention of the biological and chemical entity(ies) that are being measured.
- an explicit mention of the biological and chemical entity(ies) that are altered/varied/perturbed in a controlled manner.
- the exact sample size (n) for each experimental group/condition, given as a number, not a range;
- a description of the sample collection allowing the reader to understand whether the samples represent technical or biological replicates (including how many animals, litters, cultures, etc.).
- a statement of how many times the experiment shown was independently replicated in the laboratory.
- definitions of statistical methods and measures:
  - common tests, such as t-test (please specify whether paired vs. unpaired), simple χ2 tests, Wilcoxon and Mann-Whitney tests, can be unambiguously identified by name only, but more complex techniques should be described in the methods section;
  - are tests one-sided or two-sided?
  - are there adjustments for multiple comparisons?
  - exact statistical test results, e.g., P values = x but not P values < x;
  - definition of 'center values' as median or average;
  - definition of error bars as s.d. or s.e.m.

Please complete ALL of the questions below.
Select "Not Applicable" only when the requested information is not relevant for your study.

Materials

Newly Created Materials	Information included in the manuscript?	In which section is the information available? (Reagents and Tools Table, Materials and Methods, Figures, Data Availability Section)
New materials and reagents need to be available; do any restrictions apply?	Not Applicable	

Antibodies	Information included in the manuscript?	In which section is the information available? (Reagents and Tools Table, Materials and Methods, Figures, Data Availability Section)
For **antibodies** provide the following information:<br>- Commercial antibodies: RRID (if possible) or supplier name, catalogue number and or/clone number<br>- Non-commercial: RRID or citation	Yes	Materials and Methods

DNA and RNA sequences	Information included in the manuscript?	In which section is the information available? (Reagents and Tools Table, Materials and Methods, Figures, Data Availability Section)
**Short novel DNA or RNA including primers, probes**: provide the sequences.	Yes	Materials and Methods

Cell materials	Information included in the manuscript?	In which section is the information available? (Reagents and Tools Table, Materials and Methods, Figures, Data Availability Section)
**Cell lines:** Provide species information, strain. Provide accession number in repository **OR** supplier name, catalog number, clone number, and/**OR** RRID.	Not Applicable	
**Primary cultures:** Provide species, strain, sex of origin, genetic modification status.	Not Applicable	
Report if the cell lines were recently **authenticated** (e.g., by STR profiling) and tested for mycoplasma contamination.	Not Applicable	

Experimental animals	Information included in the manuscript?	In which section is the information available? (Reagents and Tools Table, Materials and Methods, Figures, Data Availability Section)
**Laboratory animals or Model organisms:** Provide species, strain, sex, age, genetic modification status. Provide accession number in repository **OR** supplier name, catalog number, clone number, **OR** RRID.	Not Applicable	
**Animal observed in or captured from the field:** Provide species, sex, and age where possible**.**	Not Applicable	
Please detail **housing and husbandry conditions**.	Not Applicable	

Plants and microbes	Information included in the manuscript?	In which section is the information available? (Reagents and Tools Table, Materials and Methods, Figures, Data Availability Section)
**Plants:** provide species and strain, ecotype and cultivar where relevant, unique accession number if available, and source (including location for collected wild specimens).	Yes	Materials and Methods
**Microbes:** provide species and strain, unique accession number if available, and source.	Not Applicable	

Human research participants	Information included in the manuscript?	In which section is the information available? (Reagents and Tools Table, Materials and Methods, Figures, Data Availability Section)
If collected and within the bounds of privacy constraints report on age, sex and gender or ethnicity for all study participants.	Not Applicable	

Core facilities	Information included in the manuscript?	In which section is the information available? (Reagents and Tools Table, Materials and Methods, Figures, Data Availability Section)
If your work benefited from core facilities, was their service mentioned in the acknowledgments section?	Yes	Acknowledgements

Design

Study protocol	Information included in the manuscript?	In which section is the information available? (Reagents and Tools Table, Materials and Methods, Figures, Data Availability Section)
If study protocol has been **pre-registered, provide DOI in the manuscript**. For clinical trials, provide the trial registration number **OR** cite DOI.	Not Applicable	
Report the **clinical trial registration number** (at ClinicalTrials.gov or equivalent), where applicable.	Not Applicable	

Laboratory protocol	Information included in the manuscript?	In which section is the information available? (Reagents and Tools Table, Materials and Methods, Figures, Data Availability Section)
Provide DOI OR other citation details if **external detailed step-by-step protocols** are available.	Not Applicable	

Experimental study design and statistics	Information included in the manuscript?	In which section is the information available? (Reagents and Tools Table, Materials and Methods, Figures, Data Availability Section)
Include a statement about **sample size** estimate even if no statistical methods were used.	Yes	Materials and Methods
Were any steps taken to minimize the effects of subjective bias when allocating animals/samples to treatment (e.g. **randomization procedure**)? If yes, have they been described?	Yes	Materials and Methods
Include a statement about **blinding** even if no blinding was done.	Not Applicable	
Describe **inclusion/exclusion criteria** if samples or animals were excluded from the analysis. Were the criteria pre-established?<br><br>If sample or data points were omitted from analysis, report if this was due to attrition or intentional exclusion and provide justification.	Yes	Materials and Methods
For every figure, are **statistical tests** justified as appropriate? Do the data meet the assumptions of the tests (e.g., normal distribution)? Describe any methods used to assess it. Is there an estimate of variation within each group of data? Is the variance similar between the groups that are being statistically compared?	Yes	Materials and Methods

Sample definition and in-laboratory replication	Information included in the manuscript?	In which section is the information available? (Reagents and Tools Table, Materials and Methods, Figures, Data Availability Section)
In the figure legends: state number of times the experiment was **replicated** in laboratory.	Yes	Results
In the figure legends: define whether data describe **technical or biological replicates**.	Yes	Results

Ethics

Ethics	Information included in the manuscript?	In which section is the information available? (Reagents and Tools Table, Materials and Methods, Figures, Data Availability Section)
Studies involving **human participants**: State details of **authority granting ethics approval** (IRB or equivalent committee(s), provide reference number for approval.	Not Applicable	
Studies involving **human participants**: Include a statement confirming that **informed consent** was obtained from all subjects and that the experiments conformed to the principles set out in the WMA Declaration of Helsinki and the Department of Health and Human Services Belmont Report.	Not Applicable	
Studies involving **human participants:** For publication of **patient photos**, include a statement confirming that consent to publish was obtained.	Not Applicable	
Studies involving experimental **animals**: State details of **authority granting ethics approval** (IRB or equivalent committee(s), provide reference number for approval. Include a statement of compliance with ethical regulations.	Not Applicable	
Studies involving **specimen and field samples:** State if relevant **permits** obtained, provide details of authority approving study; if none were required, explain why.	Not Applicable	

Dual Use Research of Concern (DURC)	Information included in the manuscript?	In which section is the information available? (Reagents and Tools Table, Materials and Methods, Figures, Data Availability Section)
Could your study fall under dual use research restrictions? Please check biosecurity documents and list of **select agents and toxins** (CDC): https://www.selectagents.gov/sat/list.htm	Not Applicable	
If you used a select agent, is the security level of the lab appropriate and reported in the manuscript?	Not Applicable	
If a study is subject to dual use research of concern regulations, is the name of the **authority granting approval and reference number** for the regulatory approval provided in the manuscript?	Not Applicable	

Reporting

The MDAR framework recommends adoption of discipline-specific guidelines, established and endorsed through community initiatives. Journals have their own policy about requiring specific guidelines and recommendations to complement MDAR.

Adherence to community standards	Information included in the manuscript?	In which section is the information available? (Reagents and Tools Table, Materials and Methods, Figures, Data Availability Section)
State if relevant guidelines or checklists (e.g., **ICMJE, MIBBI, ARRIVE, PRISMA**) have been followed or provided.	Not Applicable	
For **tumor marker prognostic studies,** we recommend that you follow the **REMARK** reporting guidelines (see link list at top right). See author guidelines, under 'Reporting Guidelines'. Please confirm you have followed these guidelines.	Not Applicable	
For **phase II and III randomized controlled trials**, please refer to the **CONSORT** flow diagram (see link list at top right) and submit the CONSORT checklist (see link list at top right) with your submission. See author guidelines, under 'Reporting Guidelines'. Please confirm you have submitted this list.	Not Applicable	

Data Availability

Data availability	Information included in the manuscript?	In which section is the information available? (Reagents and Tools Table, Materials and Methods, Figures, Data Availability Section)
Have **primary datasets** been deposited according to the journal's guidelines (see 'Data Deposition' section) and the respective accession numbers provided in the Data Availability Section?	Yes	Data Availability Section
Were **human clinical and genomic datasets** deposited in a public access-controlled repository in accordance to ethical obligations to the patients and to the applicable consent agreement?	Not Applicable	
Are **computational models** that are central and integral to a study available without restrictions in a machine-readable form? Were the relevant accession numbers or links provided?	Yes	Material and Methods
If publicly available data were reused, provide the respective **data citations in the reference list**.	Yes	Reference
